# The small molecule C-6 is selectively cytotoxic against breast cancer cells and its biological action is characterized by mitochondrial defects and endoplasmic reticulum stress

**DOI:** 10.1186/s13058-014-0472-0

**Published:** 2014-11-26

**Authors:** Rachel M Vaden, Keith M Gligorich, Ranjan Jana, Matthew S Sigman, Bryan E Welm

**Affiliations:** 1 Department of Chemistry, University of Utah, 315 South 1400 East, Salt Lake City, 84112 Utah USA; 2 Department of Oncological Sciences, Huntsman Cancer Institute, University of Utah, 2000 Circle of Hope Drive, Salt Lake City, 84112 Utah USA; 3 Immunobiology and Cancer Program, Oklahoma Medical Research Foundation, 825 Northeast 13th Street, Oklahoma City, 73104 OK USA

## Abstract

**Introduction:**

The establishment of drug resistance following treatment with chemotherapeutics is strongly associated with poor clinical outcome in patients, and drugs that target chemoresistant tumors have the potential to increase patient survival. In an effort to identify biological pathways of chemoresistant breast cancers that can be targeted therapeutically, a small molecule screen utilizing metastatic patient-derived breast cancer cells was conducted; from this previous report, the cytotoxic small molecule, C-6, was identified for its ability to selectively kill aggressive breast cancer cells in a caspase-independent manner. Here, we describe the cellular and molecular pathways induced following C-6 treatment in both normal and breast cancer cell lines.

**Methods:**

Transcriptome analyses and protein expression experiments were used to measure endoplasmic reticulum (ER) stress following C-6 treatment. Studies utilizing transmission electron microscopy and metabolomic profiling were conducted to characterize mitochondrial morphology and function in C-6-treated cells. Oxygen consumption rates and oxidative stress were also measured in breast cancer and normal mammary epithelial cells following treatment with the small molecule. Finally, structural modifications were made to the molecule and potency and cancer selectivity were evaluated.

**Results:**

Treatment with C-6 resulted in ER stress in both breast cancer cells and normal mammary epithelial cells. Gross morphological defects were observed in the mitochondria and these aberrations were associated with metabolic imbalances and a diminished capacity for respiration. Following treatment with C-6, oxidative stress was observed in three breast cancer cell lines but not in normal mammary epithelial cells. Finally, synthetic modifications made to the small molecule resulted in the identification of the structural components that contribute to C-6’s cancer-selective phenotype.

**Conclusions:**

The data reported here implicate mitochondrial and ER stress as a component of C-6’s biological activity and provide insight into non-apoptotic cell death mechanisms; targeting biological pathways that induce mitochondrial dysfunction and ER stress may offer new strategies for the development of therapeutics that are effective against chemoresistant breast cancers.

**Electronic supplementary material:**

The online version of this article (doi:10.1186/s13058-014-0472-0) contains supplementary material, which is available to authorized users.

## Introduction

The efficacy of widely used cancer chemotherapeutics is frequently limited by side effects resulting from narrow therapeutic windows between a drug’s beneficial anticancer activity and its general cytotoxicity. An additional limitation of widely used anticancer drugs is the development of multidrug chemoresistance [1]. It has therefore been an interest of our laboratories to identify novel small molecules that selectively kill cancer cells, and use these compounds as tools to define biological pathways necessary for the viability of breast cancer cells. We previously reported the identification of such a molecule, C-6, and established its ability to kill, in a caspase-independent manner, metastatic primary cells from patients with chemoresistant breast cancer [2]. Herein, we report the results of studies on the biological activity of C-6 and describe structure-activity relationships for the molecule’s cancer-selective cytotoxicity.

## Methods

### Cell lines

MCF-7, MCF-10A, and T47D cells were a generous gift from Andrea Bild at the University of Utah; MDA-MB-231 cells were obtained directly from the American Type Culture Collection (ATCC). All cell lines were authenticated within six months of manuscript preparation by the ATCC in conjunction with Promega (Madison, WI, USA) using short tandem repeat analysis.

### Tissue culture

MCF-10A and MCF-7 cells were cultured with Dulbecco’s modified Eagle’s medium (DMEM)/F12 media containing 2.5 mM L-glutamine and 15 mM HEPES buffer (Life Technologies, Grand Island, NY, USA). MDA-MB-231 and T47D cells were cultured with RPMI-1640 medium containing 2.5 mM L-glutamine and 25 mM HEPES buffer (Life Technologies). All cells were cultured at 37°C with 5% CO_2_. For standard culture conditions, medias were supplemented with 10% fetal bovine serum (FBS) (heat inactivated, HyClone, Logan, UT, USA) penicillin-streptomycin-glutamine, 5.0 μg/mL of insulin-transferrin-selenium-X (ITS-X) (Life Technologies), and 2.5 nM epidermal growth factor (EGF), recombinant human (BD Biosciences, San Jose, CA, USA). In the case of treatment with C-6 or its analogs, low serum medias were used (2% fetal bovine serum).

### Reagents and antibodies

Preparation procedures and characterization data for C-6 and its analogs can be found in a supplementary file (Additional file 1). The following primary antibodies were purchased from Cell Signaling (Danvers, MA, USA): CHOP, GRP78, p-EIF2α, pan-EIF2α, p-JNK, and pan-JNK. In addition, the vinculin antibody was obtained from Sigma-Aldrich (St. Louis, MO, USA). Actinomycin D (ACTD) and cycloheximide (CHX) were also obtained from Sigma-Aldrich.

### Dose response assays

All cells were seeded in clear 96-well plates (Costar, Tewksbury, MA, USA) in 100 μL of their respective media at densities necessary to achieve 90% confluency at the end of the 5-day assay. Dimethyl sulfoxide (DMSO) stocks of the compounds were diluted in their corresponding media containing 2% FBS and an EP Motion 5075 (Eppendorf North America, Hauppauge, NY, USA) liquid handler was utilized to prepare serial dilutions; a vehicle control corresponding to the highest DMSO concentration, which did not exceed 0.2% (v/v), was also prepared. Following an overnight incubation, the media was aspirated and the cells were treated with the serially diluted compounds and vehicle control. Every 48 hours, the drug-containing media was refreshed. Following 5 days of treatment, cell viability was measured using a CellTiter 96 AQueous One Solution Cell Proliferation assay per the manufacturer’s protocol (Promega). Blank-subtracted absorbance values were obtained at 490 nm and normalized to the DMSO vehicle control wells. Normalized values were plotted as an average ± standard deviation of three replicates then analyzed using the dose-response nonlinear curve fitting function with Prism 6.0 (GraphPad Software, San Diego, CA, USA) to establish the EC_50_.

### Transcriptome sequencing

In preparation for transcriptome sequencing, MCF-7 cells were seeded in triplicate in 10-cm tissue culture-treated plates such that the plate would be 80% confluent at the time of drug treatment. Cells were treated with 10 mL of low serum media containing either 30 μM C-6 or DMSO as a vehicle control for 3 hours. Following the completion of treatment, RNA was isolated using an RNeasy RNA isolation and purification kit (Qiagen, Hilden, Germany) per the manufacturer’s protocol.

Library construction was performed using the Illumina TruSeq Stranded mRNA Sample Preparation Kit (Illumina, San Diego, CA, USA) as described herein. Briefly, total RNA (100 ng to 4 ug) was poly-A selected using poly-T Oligo-attached magnetic beads. Poly-A RNA eluted from the magnetic beads was fragmented and primed with random hexamers in preparation for cDNA synthesis. First-strand reverse transcription was accomplished using Superscript II Reverse Transcriptase (Invitrogen, Waltham, MA, USA). Second-strand cDNA synthesis was accomplished using DNA polymerase I and Rnase H under conditions in which dUTP is substituted for dTTP, yielding blunt-ended cDNA fragments. An A-base was added to the blunt ends in preparation for adapter ligation and to prevent concatemer formation during the ligation step. Adapters containing a T-base overhang were ligated to the A-tailed DNA fragments. Ligated fragments were PCR-amplified (12 to 15 cycles) under conditions that enabled only amplification of the first-strand cDNA product. The PCR-amplified library was purified using Agencourt AMPure XP beads (Beckman Coulter Genomics, Danvers, MA, USA). The concentration of the amplified library was measured with a NanoDrop spectrophotometer and an aliquot of the library was resolved on an Agilent 2200 Tape Station using a D1K or a High Sensitivity D1K assay (Agilent Technologies, Santa Clara, CA, USA) to define the size distribution of the sequencing library. Libraries were adjusted to a concentration of approximately 10 nM and quantitative PCR was performed using the Kapa Library Quantification Kit (Kapa Biosystems, Boston, MA, USA) to calculate the molarity of adapter-ligated library molecules. The concentration was further adjusted following qPCR to prepare the library for sequence analysis on an Illumina HiSeq instrument. Following completion of sequencing, genome alignment was conducted using National Center for Biotechnology Information (NCBI) build GRch37 and differential expression analysis was performed using the RNAseq application [3], which wraps the DESeq Bioconductor package, and statistical significance was calculated as described by Anders and Huber [4].

### Western blot analyses

For Western blot analyses, cells were lysed in ice-cold radioimmunoprecipitation assay buffer (pH = 8.0, 50 mM 150 mM NaCl, Tris HCl, 0.1% sodium dodecyl sulfate (SDS), 0.5% sodium deoxycholate, 1% triton-X-100,) supplemented with protease inhibitor cocktail (Sigma-Aldrich), phosphatase inhibitor cocktail 2 (Sigma-Aldrich), and 1 mM dithiothreitol (DTT) (Sigma-Aldrich). The lysate was sonicated for 30 seconds with a 450 Sonifier (Branson Ultrasonics, Danbury, CT, USA) and then centrifuged at 14,000 RPM for 5 minutes at 4°C. A bicinchoninic acid (BCA) protein assay kit (Pierce, Rockford, IL, USA) was used to measure the protein concentration and the samples were subsequently boiled for 5 minutes in 4x SDS Laemmli buffer. Proteins were separated on SDS polyacrylamide gels and transferred to a Immobilon-FL PVDF membrane (EMD Millipore, Billerica. MA, USA). The blots were blocked in Odyssey Blocking Buffer (LI-COR, Lincoln, NE, USA) for 1 hour at room temperature, stained with the primary antibodies overnight at 4°C, then stained with IR800CW or IR680 anti-mouse or rabbit secondary antibodies (LI-COR) for 1 hour at room temperature. The blots were imaged with the Odyssey Infrared Imaging System (LI-COR).

### Cell imaging

In tissue culture-treated six-well plates, cells were seeded at 200,000 cells per well in 3 mL of their respective media. After allowing the cells to recover for 48 hours, the media was aspirated and replaced with 4 mL of low serum media containing 30 μM C-6 or DMSO as a vehicle control. For the duration of the time course imaging, cells were maintained at 37°C in a humidified chamber with 5% CO_2_. Images were acquired using an IX81 microscope (Olympus, Center Valley, PA, USA) running Slide Book 5.0 software (Intelligent Imaging Innovations, Denver, CO, USA). For experiments utilizing MitoTracker Deep Red FM (Life Technologies), cells were drug treated for the appropriate amount of time, then MitoTracker added directly to the well to achieve a final concentration of 100 nM. The plate was then incubated for 10 minutes at 37°C, the dye-containing media discarded and replaced with fresh drug-containing media, and images acquired.

### Transmission electron microscopy

MCF-10A and MCF-7 cells were cultured in 10-cm plates in preparation for transmission electron microscopy (TEM) analysis. Following the completion of treatment with 10 mL of either 30 μM C-6 or a matched DMSO vehicle control, the cells were fixed in a pH 7.4 0.1 M sodium cacodylate buffer containing 2.5% glutaraldehyde, 1% paraformaldehyde, 2.4% sucrose, and 8 mM calcium chloride. After fixation, the cells were rinsed in 0.1 M sodium cacodylate buffer and post-fixed in 2% osmium tetroxide also in the 0.1 M sodium cacodylate buffer. Cells were rinsed in type 1 water and *en* *bloc* stained in saturated aqueous uranyl acetate. Cells were then dehydrated in a graded ethanol series, transitioned through acetone, infiltrated with Embed 812 and acetone, embedded in fresh Embed 812, and allowed to cure overnight in a 60°C oven. Plastic sections were cut on a Leica (Wetzlar, Germany) ultramicrotome with a diamond knife and placed on copper grids at a thickness of 80 to 100 nm. Sections were contrasted with saturated aqueous uranyl acetate followed by Reynold’s lead citrate. They were then examined on an FEI Tecnai (Hillsboro, OR, USA) T-12 TEM with a LaB6 filament at 120KV. Images were acquired with a Gatan (Pleasanton, CA, USA) Ultrascan 1000 digital camera using Gatan’s digital micrograph.

### Metabolomic profiling

Cells were cultured in 10-cm plates and following the completion of the appropriate drug treatments, the cells were collected and pelleted, resuspended in 90% MeOH, and flash frozen in liquid nitrogen. Ten microliters of a 0.2 ug/uL solution of D4-succinate was added to each sample as an internal control. All GC-MS analysis was performed with a Waters (Milford, MA, USA) GCT Premier mass spectrometer fitted with an Agilent 6890 gas chromatograph and a Gerstel (Mülheim an der Ruhr, Germany) MPS2 autosampler. Dried samples were suspended in 40 uL of a 40 mg/mL O-methoxylamine hydrochloride (MOX) in pyridine and incubated for 1 hour at 30°C. To the autosampler vials was added 25 uL of this solution. Ten microliters of N-methyl-N-trimethylsilyltrifluoracetamide (MSTFA) was added automatically via the autosampler and incubated for 60 minutes at 37°C with shaking. After incubation, 3 uL of a fatty acid methyl ester standard solution was added via the autosampler then 1 uL of the prepared sample was injected to the gas chromatograph inlet in the split mode with the inlet temperature held at 250°C. Two GC-MS runs were performed, one at a 10:1 split ratio to detect low-level metabolites and a second at 50:1 split ratio to accurately measure high-concentration metabolites, which saturate the detector at the 10:1 split ratio. For the 10:1 split ratio analysis, the gas chromatograph had an initial temperature of 95°C for 1 minute followed by a 40°C/minute ramp to 110°C and a hold time of 2 minutes. This was followed by a second 5°C/minute ramp to 250°C, a third ramp to 350°C, then a final hold time of 3 minutes. For the 50:1 split ratio analysis, the gas chromatograph had an initial temperature of 95°C for 1 minute followed by a 40°C/minute ramp to 110°C and a hold time of 2 minutes. This was followed by a second 25°C/minute ramp to 330°C. A 30 m Phenomex (Torrance, CA, USA) ZB5-5 MSi column with a 5-m-long guard column was employed for chromatographic separation. Helium was used as the carrier gas at 1 mL/minute.

### Measurement of oxygen consumption and extracellular acidification rates

Cells were seeded in XF24 V7 culture plates (Seahorse Bioscience, North Billerica, MA, USA) in 300 μL of their respective media. After culturing overnight, the media was aspirated and the cells were treated with 600 μL of media containing 2% FBS and 30 μM C-6 for 3, 6, 12, and 24 hours as well as 0.03% (v/v) DMSO (four wells per condition). The media was aspirated and 600 μL of unbuffered assay media at pH = 7.4, which consists of DMEM supplemented with 2 mM GlutaMax-1 (Life Technologies), 1 mM sodium pyruvate (Sigma-Aldrich), 25 mM glucose (Sigma-Aldrich), and 31.7 mM NaCl was added and the cells were incubated at 37°C for 1 hour. Utilizing the XF24 Extracellular Flux Analyzer (Seahorse Biosciences), the baseline oxygen consumption rate (OCR) and extracellular acidification rate (ECAR) was measured. For the mitochondrial stress test, oligomycin A (Oligo, Sigma-Aldrich), which inhibits ATP synthase, was subsequently added to a final concentration of 1 μg/mL followed by addition of carbonyl cyanide 4-(trifluoromethoxy)phenylhydrazone (FCCP) (Sigma-Aldrich) to a concentration of 0.5 μM. Finally, a mixture of myxothiazol (Myx, Sigma-Aldrich) and rotenone (Rot, Sigma-Aldrich) were added to a final concentration of 0.5 μM and 1 μM respectively to inhibit complexes I and III of the electron transport chain.

### Measurement of oxidative stress

Oxidative stress was measured by the fluorescent indicator 2′,7′-dichlorodihydrofluorescein diacetate (H_2_DCFDA, Life Technologies). Briefly, cells were seeded in triplicate in tissue culture-treated 15-mm plates at 500,000 cells per plate in 3 mL of their respective media. After allowing the cells to recover for 48 hours, the media was aspirated and replaced with 3 mL of low serum media containing 30 μM C-6 or DMSO as a vehicle control for 3, 6, 12, 24, or 48 hours. Following the completion of treatment, the media was discarded and replaced with 1 mL of Hank’s balanced salt solution (HBSS) containing 30 μM H_2_DCFDA and the cells incubated for 30 minutes at 37°C. The staining media was then discarded and the cells trypsinized and analyzed by a FACscan flow cytometer (Becton Dickinson, Mountain View, CA, USA) for fluorescence.

### Real-time PCR (RT-PCR)

For experiments designed to measure gene expression, real-time PCR (RT-PCR) was conducted using a LightCycler 480 (Roche, Basel, Switzerland). Following drug treatment, RNA was isolated using an RNeasy RNA isolation and purification kit (Qiagen) per the manufacturer’s protocol. Genomic DNA was removed with a DNase digest and 1 μg of RNA was used to synthesize cDNA using a Superscript III Reverse Transcriptase kit from Invitrogen per the manufacturer’s protocol; after an RNase H digestion, RT-PCR was performed in a 5 μL reaction using KAPA SYBR FAST qPCR Master Mix (Kapa Biosystems, Boston, MA, USA). The following primer sets were used: CHOP 5′-*AGTCTCTCCTCGGCTTGC*-3′ and 5′-*ACATCTGGGAGAAAGGTTGTC*-3′ [5]. Data were normalized to an internal reference gene (GAPDH 5′-*AAATTCCATGGCACCGTC*-3′ and 5′-*GATGGTGATGGGATTTCCA*-3′) and relative gene expression was assessed using the comparative CT method [6].

### Measurement of caspase activity

Caspase activity was measured using the Caspase-Glo assay (Promega) for caspases 3/7, 8, and 9 according to the manufacturer’s protocol.

### Ethical considerations

No human or animal experiments were conducted; ethical approval from an independent review committee was not required for the studies conducted and presented herein.

### Statistics

For *in vitro* assays, an unpaired Student’s *t* test was performed using GraphPad Prism 6.0 or Microsoft Excel (Microsoft, Redmond, WA, USA) and *P* values <0.05 between groups were considered significant.

## Results

### The cytotoxic molecule C-6 is selective for cancer cells

**Figure 1:**
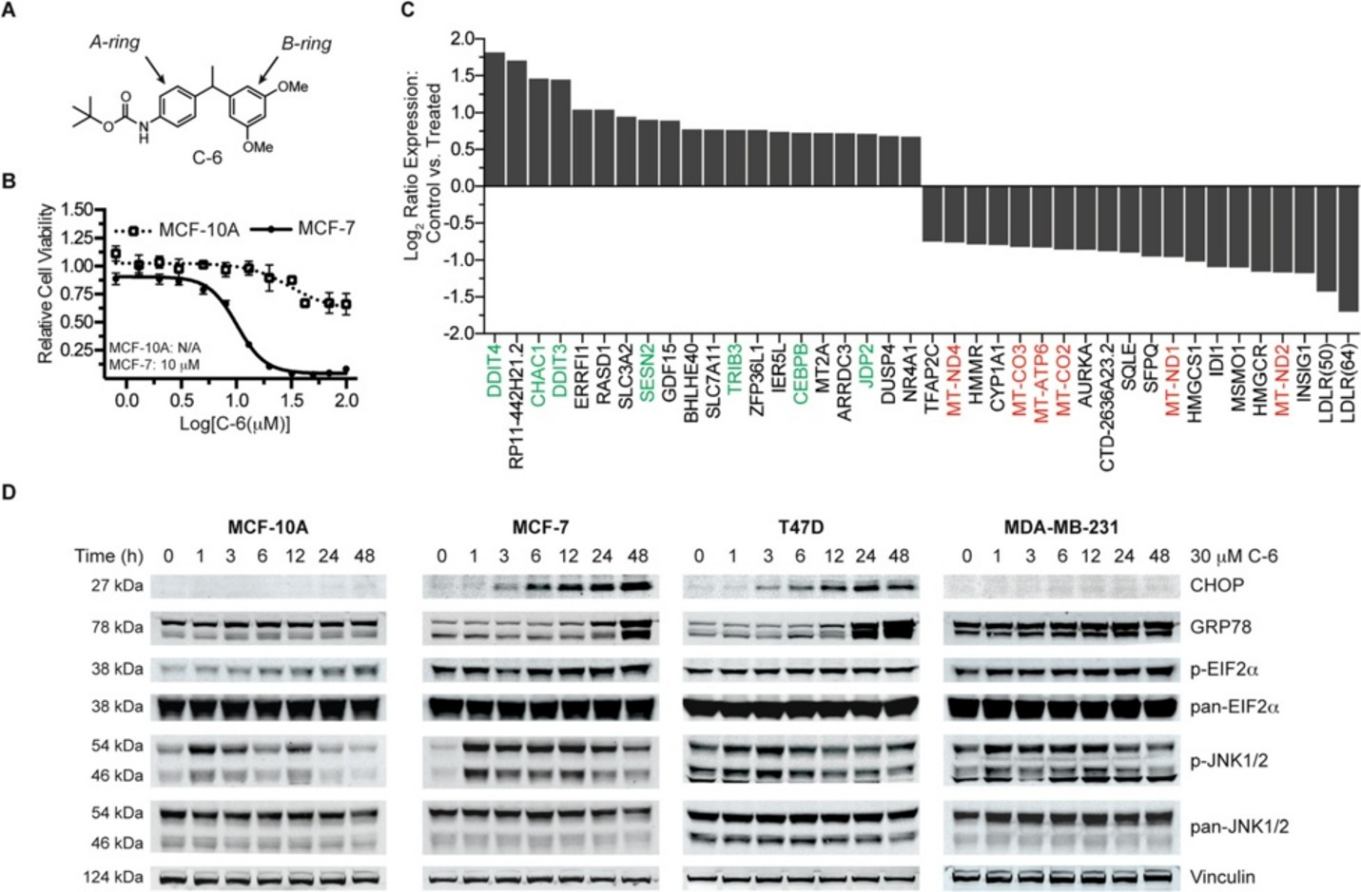
**Characterization of C-6-induced endoplasmic reticulum (ER) stress. (A)** The anticancer small molecule C-6. **(B)** A dose-response analysis of C6-treated MCF-7 breast cancer cells and MCF-10A mammary epithelial cells measuring cell viability. Error bars represent the ± standard deviation of three replicates. **(C)** Analysis of the 20 most differentially expressed (upregulated and downregulated) MCF-7 transcripts following a 3-hour treatment with 30 μM C-6 (in triplicate). Results are plotted as a log_2_ ratio of the treated versus control samples. **(D)** Protein expression analysis of proteins associated with the ER stress response pathway following a time course treatment with 30 μM C-6.

In a previous report, we identified a novel diarylmethine-containing small molecule, C-6, that selectively kills breast cancer cells (Figure 1A) [2]. Specifically, the small molecule was found to be cytotoxic against chemoresistant patient-derived primary cells in addition to multiple breast cancer cell lines at low micromolar concentrations. This cytotoxicity against malignant cells was found to be in contrast to the largely benign effect of C-6 against *in vitro* models of normal mammary epithelial tissue (Figure 1B).

### Expression profiling reveals C-6 induces mitochondrial and endoplasmic reticulum stress pathways

As a first approach toward further characterizing C-6’s mechanism of action and understanding the biological basis for its cancer selectivity, a next-generation RNA sequencing experiment was conducted to profile the transcriptome following C-6 treatment. MCF-7 breast cancer cells were treated in triplicate with either 30 μM C-6 or a matched DMSO vehicle control for 3 hours. Following RNA isolation and subsequent sequencing, a differential expression analysis was performed to identify transcriptional changes resulting from treatment with the small molecule (Figure 1C). Of the nearly 18,000 genes profiled in the experiment, 68 genes were found to have statistically significant differences in their expression levels compared to the control samples. Interestingly, seven of the top twenty genes whose expression was upregulated compared to controls were associated with endoplasmic reticulum (ER) stress response pathways [7]-[11]. Additionally, six of the twenty most downregulated genes were encoded by the mitochondrial genome, suggesting mitochondrial stress as a component in C-6’s phenotype.

In order to determine if ER stress response genes identified from the MCF-7 transcriptome profiling experiment were upregulated at the protein level, known ER stress proteins were assayed by Western blot in three breast cancer cell lines (MCF-7, T47D, and MDA-MB-231) and one normal mammary epithelial cell line (MCF-10A) (Figure 1D); the results of the Western blots were also quantified by densitometric analysis (Additional file 2). A time-course treatment study over 48 hours using 30 μM C-6 revealed that the expression of DDIT3 (CCAAT-enhancer-binding protein homologous protein, CHOP), a transcription factor whose expression during ER stress can promote cell death, was increased markedly in the MCF-7 and T47D cell lines but was not detected in the MCF-10A or MDA-MB-231 cell lines [12]. GRP78, a protein-folding chaperone in the ER whose expression is frequently increased during periods of protein misfolding, was increased in MCF-7, T47D, and MDA-MB-231 breast cancer cell lines but not in the C-6-insensitive MCF-10A normal mammary epithelial cell line [13]. The relative levels and phosphorylation status of EIF2α, which is phosphorylated upon induction of ER stress, were also analyzed by Western blot following treatment with C-6 [14]. We found that EIF2α was phosphorylated in all of the cell lines examined but to varying degrees. A relatively high basal level of EIF2α phosphorylation was also observed in the three cancer cell lines compared to the normal mammary epithelial cell line. Finally, the phosphorylation of c-Jun Kinase (JNK) was measured following a time-course treatment with C-6. The JNK pathway can be activated by the accumulation of unfolded proteins in the lumen of the ER and JNK signaling has been shown to potentiate pro-death pathways [15]-[18]. A striking increase in phosphorylation was observed in MCF-10A and MCF-7 cells after 1 hour of C-6 treatment; T47D and MDA-MB-231 cells also presented with an increase in JNK phosphorylation but this effect was attenuated compared with MCF-10A and MCF-7 cells. Collectively, differential gene expression profiling and protein expression measurements reveal ER stress to be a component of C-6’s biological activity although the magnitude of this response was found to vary between cell types. These data suggest a possible role for mitochondrial and ER stress in C-6-induced cell death.

### C-6 induces mitochondrial enlargement

**Figure 2:**
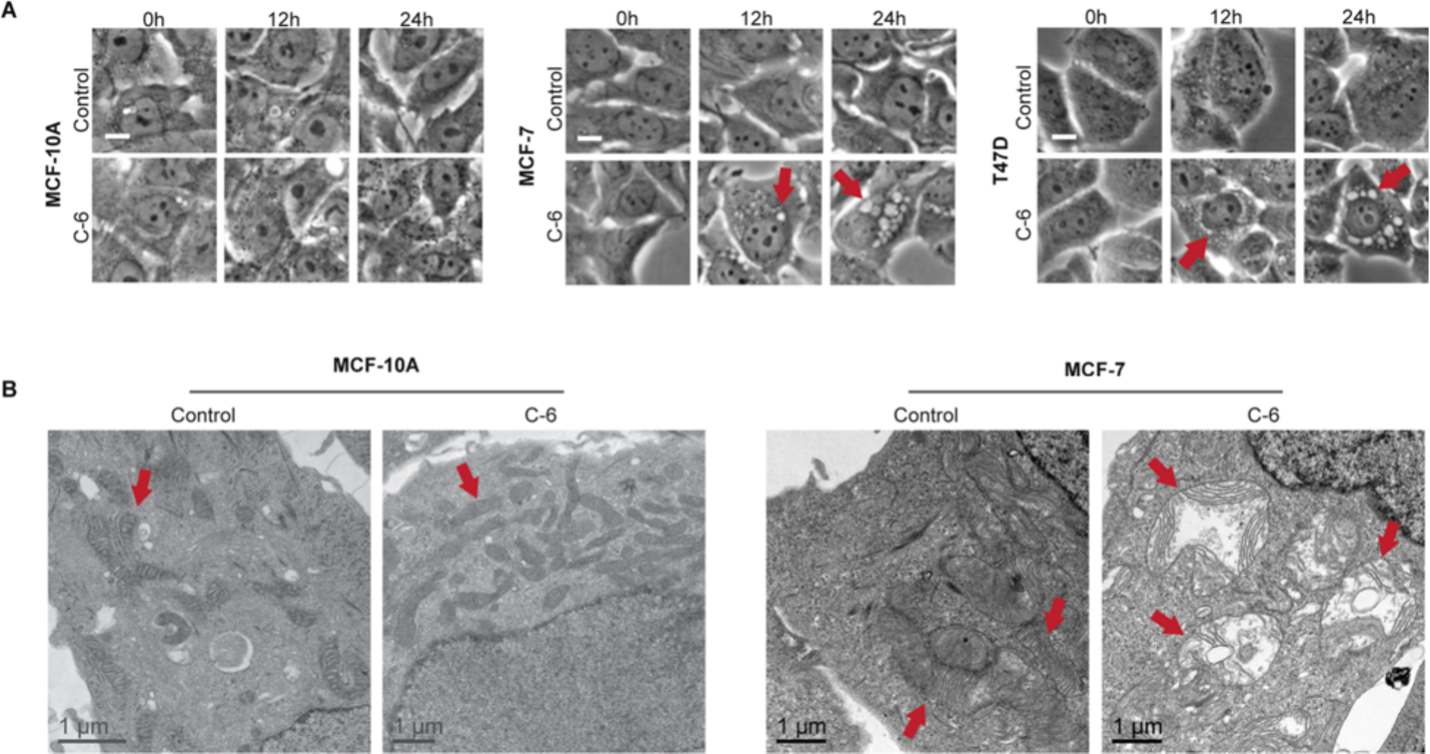
**C-6 affects mitochondrial morphology. (A)** Phase contrast images of MCF-10A, MCF-7, and T47D cells following treatment with either 30 μM C-6 or a matched dimethyl sulfoxide (DMSO) vehicle control. Scale bar represents 10 μm. **(B)** Transmission electron microscopy images MCF-10A and MCF-7 cells following treatment with either 30 μM C-6 or a matched DMSO vehicle control for 24 hours. Arrows denote mitochondria.

During our efforts to define the mechanism by which C-6 induces caspase-independent cell death in breast cancer cells, a unique cytoplasmic vacuole phenotype was observed in MCF-7, T47D, and MDA-MB-231 cells upon treatment with 30 μM C-6 but not in MCF-10A cells (Figure 2A and Additional file 3). The effect was apparent by phase microscopy beginning 3 hours after the start of treatment and became prominent after 24 hours. This cytoplasmic vacuole phenotype is consistent with reports of paraptosis, a form of caspase-independent programmed cell death first characterized by Sperandio and coworkers [19]. In agreement with this report, the C-6-induced vacuole phenotype was completely blocked when cells were co-treated with C-6 and inhibitors of transcription, ACTD, or translation, CHX (Additional file 3). The paraptotic form of programmed cell death has also been associated with defects in mitochondrial and ER morphology and function [19],[20]. As such, C-6-treated cells were analyzed by TEM to characterize mitochondrial and ER morphology. MCF-10A and MCF-7 cells were treated with 30 μM C-6 or a matched DMSO vehicle control for 24 hours then imaged by TEM. Interestingly, C-6-insensitive MCF-10A cells displayed no apparent differences between treated and control samples; however, significant ER and mitochondrial defects were observed in C-6-treated MCF-7 cells (Figure 2B). Consistent with published reports of paraptotic cell death, the defects observed when compared to control-treated MCF-7 cells included mitochondrial enlargement, substantial cristae disruption, increased mitochondrial matrix volume, and the presence of distended ER [21],[22]. These data further support the involvement of ER and mitochondrial stress in C-6’s mechanism of action against cancer cells, and suggest cell death may be mediated through paraptosis.

### Metabolic imbalances occur following treatment with C-6

**Figure 3:**
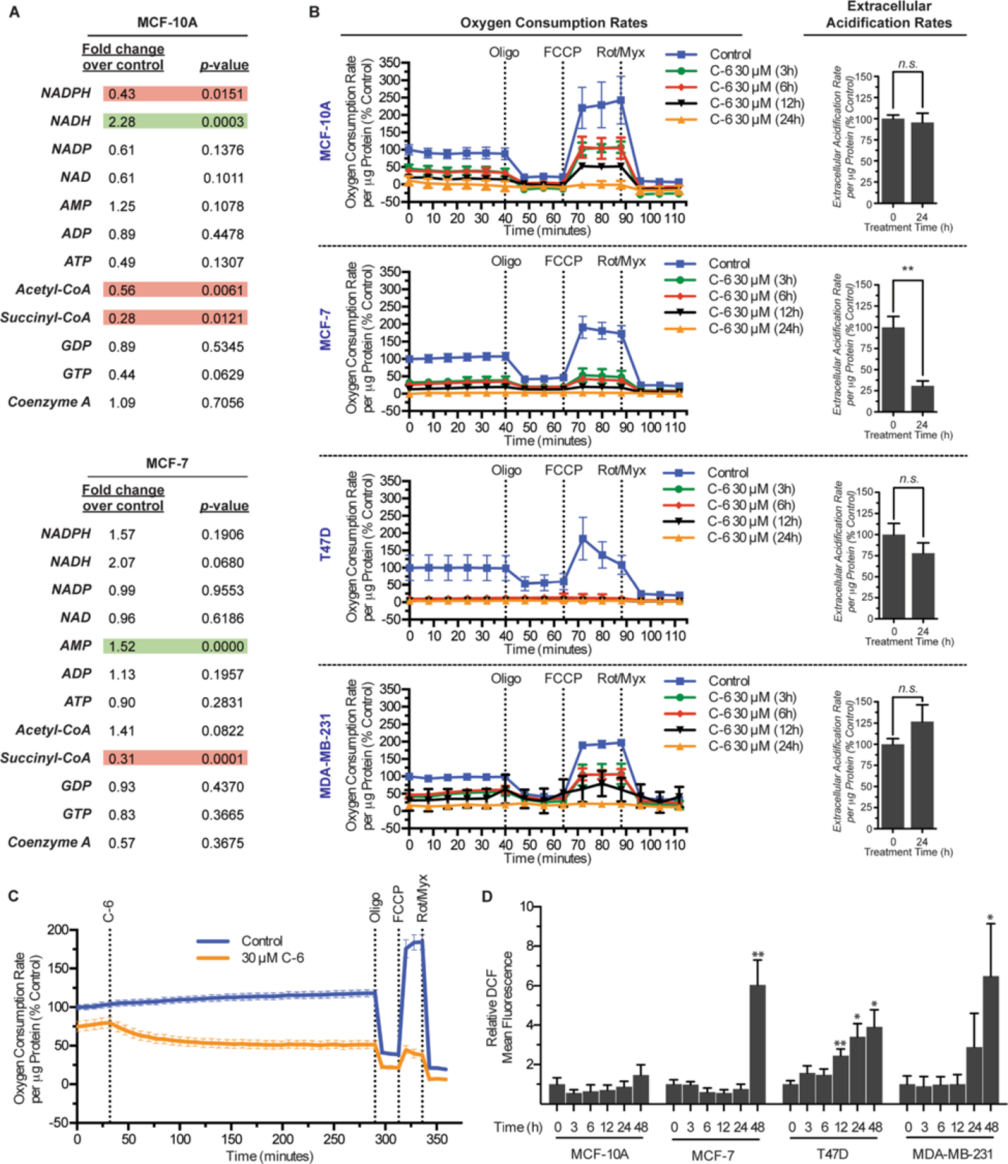
**C-6 disrupts metabolic homeostasis and energy production. (A)** Metabolomic analysis of tricarboxylic acid (TCA) cycle intermediates following a 3-hour treatment with 30 μM C-6 or a matched dimethyl sulfoxide (DMSO) vehicle control. Values represent the average of six replicates. **(B)** Measurement of oxygen consumption and extracellular acidification rates following pretreatment of cells with C-6. **(C)** Measurement of oxygen consumption rates of MCF-7 cells with simultaneous addition of 30 μM C-6. **(D)** Quantification of : 2′,7′-dichlorodihydrofluorescein diacetate (H_2_DCFDA) oxidation by flow cytometry analysis. Normalized values represent the average mean fluorescence and ± standard deviation of three independent replicates. Asterisks, ^*^ and ^**^, denote *P* values <0.05 and 0.01, respectively.

The observation that C-6-treated MCF-7 cells present with significant morphological defects of the mitochondria led us to assess mitochondrial function using metabolomic profiling of tricarboxylic acid (TCA) cycle intermediates. In order to measure acute changes in metabolic function following treatment with the small molecule, MCF-10A and MCF-7 cells were treated for 3 hours with 30 μM C-6 or a matched DMSO vehicle control. It was discovered that MCF-10A cells exhibited a statistically significant decrease in NADPH, acetyl-coenzyme A (CoA), and succinyl-CoA and an increase in NADH while MCF-7 cells displayed an increase in AMP and a decrease in the level of succinyl-CoA (Figure 3A). The metabolic intermediates measured in the experiment suggest functional defects in cellular energy production and also that MCF-10A cells, while insensitive to the cytotoxic effects of C-6, also experience altered energy metabolism as a result of C-6 treatment.

### Treatment with C-6 decreases rates of mitochondrial oxygen consumption

The altered metabolic profile observed in C-6-treated cells in conjunction with the mitochondrial defects identified by TEM prompted further studies to characterize the effects of C-6 on mitochondrial energy production. Oxygen consumption and ECARs were measured in MCF-10A, MCF-7, T47D, and MDA-MB-231 cells following a time-course treatment with either 30 μM C-6 or a matched DMSO vehicle control (Figure 3B). In each of the four cell lines examined, treatment with C-6 resulted in at least a 50% reduction in rates of basal oxygen consumption at the earliest time point tested. Specifically, MCF-10A, MDA-MB-231, and MCF-7 basal oxygen consumption was reduced by 54%, 58%, and 67% respectively while T47D cell basal respiration was dramatically reduced by 92% after only 3 hours of C-6 treatment. Longer C-6 treatment times (24 hours) resulted in an almost complete inhibition of oxygen consumption in every cell line. Despite the significant change observed in respiration, the ECARs were largely unchanged in three of the cell lines examined. In an effort to further characterize the acute effect of C-6 on respiration, the OCR of MCF-7 cells was measured simultaneously with the initiation of treatment. The results revealed that OCRs decreased within minutes following the addition of 30 μM C-6 and continued to decline for 2 hours before plateauing at approximately 50% of the starting basal levels (Figure 3C).

Experiments were also conducted to assess the functionality of the electron transport chain in the presence of C-6. While measuring OCRs, Oligo, FCCP, and a combination of Rot and Myx were added sequentially to the cells to assess the integrity of the electron transport chain and mitochondrial membrane potential [23]. Upon addition of Oligo, an inhibitor of ATP synthase, a decrease in oxygen consumption was observed in cell lines that maintained detectable respiration, suggesting that ATP synthesis occurs in conjunction with oxygen consumption and that C-6 does not uncouple energy production from electron transport [24]. The cells were then treated with FCCP to uncouple respiration followed by treatment with a combination of Rot and Myx to inhibit complex I and III activity, respectively. Of the cell lines that maintained detectable levels of oxygen consumption, the addition of FCCP resulted in an expected increase in oxygen consumption above basal levels and the addition of Rot and Myx resulted in the complete inhibition of mitochondrial respiration. Together, these data suggest that C-6-treated cells experience a time-dependent reduction in their capacity for oxidative phosphorylation even though the cells possess an intact and functional mitochondrial electron transport chain.

### Oxidative stress accompanies C-6 treatment in cancer cell lines

Considering the marked effect of C-6 treatment on mitochondrial morphology and oxidative phosphorylation, and the connection between dysfunctional mitochondria and production of reactive oxygen species (ROS), subsequent studies were conducted to assess levels of oxidative stress following treatment with the small molecule [25],[26]. MCF-10A, MCF-7, T47D, and MDA-MB-231 cells were treated with either 30 μM C-6 or a matched DMSO vehicle control for up to 48 hours and oxidative stress was then measured by flow cytometry following incubation with the ROS indicator H_2_DCFDA. After 48 hours of treatment with C-6, only the cancer cell lines displayed statistically significant increases in oxidative stress (Figure 3D). In contrast, the normal mammary epithelial cell line examined did not show any significant ROS accumulation. These data are consistent with a role of oxidative stress in caspase-independent cell death and further suggest that oxidative imbalances could play a role in C-6’s ability to selectively kill cancer cells [27]-[29].

### Structural modifications to C-6 can improve potency and cancer selectivity

**Figure 4:**
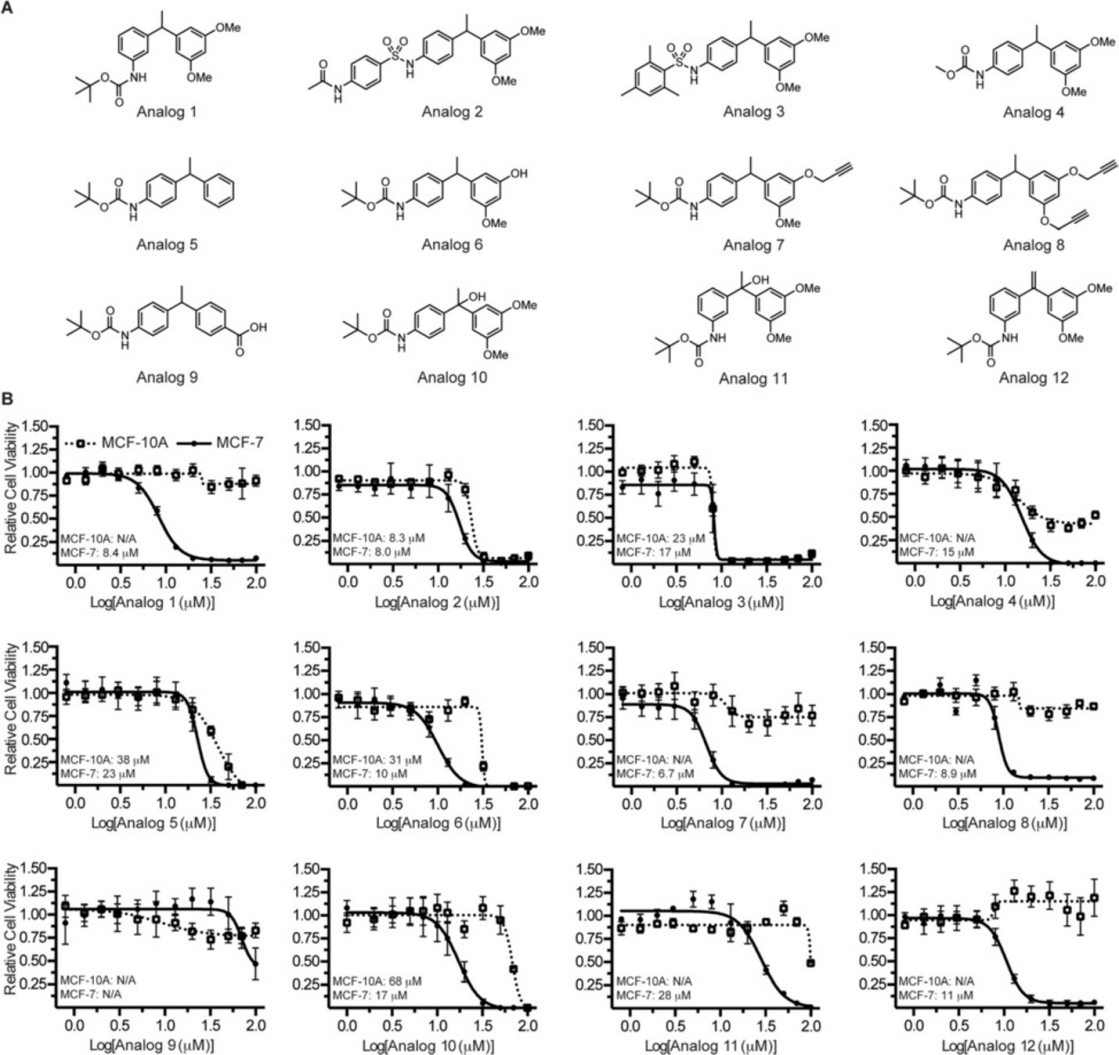
**Structural modifications alter C-6’s cancer selectivity. (A)** Analogs of C-6 designed with modifications to both the A-ring and B-ring. **(B)** The effects of C-6 structural modifications on potency and cancer selectivity as measured in a cell viability assay. Values represent the average of three replicates and error bars represent the ± standard deviation of these replicates.

In order to prepare analogs of C-6 with solubility profiles suited for intravenous injection, structure-activity relationship studies were conducted to assess the compound’s tolerance to modifications. Twelve analogs were designed to probe functional group tolerance on both the A-ring and B-ring of C-6 as well as the diarylmethine position (Figure 4A). Following their syntheses, the analogs were evaluated against MCF-10A and MCF-7 cells in a 12-point dose-response assay to measure cell viability (Figure 4B). These cell lines were selected as models to identify cytotoxic analogs that retained cancer-selective profiles. By modifying the position of the aniline group on the A-ring, we found that when the nitrogen resides at the *meta* position (analog 1), a more potent and cancer-selective profile could be obtained compared to the parent molecule. However, conversion of C-6’s carbamate ester to an aryl sulfonamide resulted in a complete loss of selectivity (analogs 2 and 3); replacing the tert-butyl group of the carbamate with a methyl group also attenuated the cancer-selective phenotype but to a lesser degree (analog 4). Of the synthetic changes made to the B-ring of C-6, removal of the two methoxy substituents (analog 5) or replacement of one methoxy group with an alcohol resulted in decreased efficacy. Modifying these same positions on the B-ring to include propargyl groups (analogs 7 and 8) resulted in slightly improved potencies and cytotoxic effects limited largely to the cancer cells; the *para*-carboxylic acid compound (analog 9) displayed a significant loss in potency in the cell viability assay. Finally, C-6 analogs containing modifications to the diarylmethine carbon were prepared and tested *in vitro*. A reduction in cancer selectivity was observed upon incorporation of a hydroxyl group at this position (analogs 10 and 11) and the presence of a structurally rigid alkene at the diarylmethine position (analog 12) had a minimal effect on the analog’s EC_50_ compared to the parent molecule. Collectively, the structural studies indicate that while C-6’s potency can tolerate a variety of functional group modifications, the cancer-selective phenotype is especially sensitive to these alterations.

Upon finding that several of the analogs either retained or improved upon the cancer-selective nature of the parent molecule C-6, studies were conducted to determine whether these analogs affected the same biological pathways as C-6. The three most potent and cancer-selective analogs (Analog 1, Analog 8, and Analog 12) were used in a panel of experiments to assess mechanistic similarities with C-6. The experiments were designed to evaluate the following characteristics in T47D cells: ER stress as measured by RT-PCR analyses of the transcript CHOP, cytoplasmic vacuole formation and mitochondrial swelling, caspase activation, mitochondrial functionality and oxygen consumption, and oxidative stress. In each of the experiments performed, the three analogs tested resulted in similar findings as with the parent molecule C-6 (Additional file 4). Based upon these findings, it is therefore likely that the cancer-selective analogs modulate the same biological pathways as C-6, resulting in an ER stress response and mitochondrial dysfunction.

## Discussion

In a previous report, our laboratories identified from a chemical library screen a small molecule that was both cytotoxic against and selective for cancer cells. Specifically, we demonstrated that the small molecule C-6 was capable of killing highly metastatic primary pleural effusion cells acquired from patients with chemoresistant breast cancer, and that the mechanism of cell death was independent of caspase activity. Since tumor cells can be defined by their ability to evade apoptosis [30],[31], non-apoptotic cell death pathways may provide alternative mechanisms by which to therapeutically target cancer cells. C-6’s unique phenotype and strong selectivity profile prompted us to expand our mechanistic studies with the goal of characterizing this non-apoptotic cell death pathway.

As a first step toward this goal, we conducted an RNA-seq experiment that demonstrated the expression of ER stress response genes was substantially upregulated and multiple genes encoded by the mitochondrial genome were significantly decreased after only 3 hours of treatment. Furthermore, Western blot analyses of four different ER stress-related proteins revealed that the induction of ER stress was common in all C-6-treated cells, although the magnitude of the stress response was varied in each of the cell types tested. Higher cancer cell proliferation rates and concomitant demands for new protein synthesis result in an increased burden on the protein folding machinery of the ER; in addition, glucose dyshomeostasis and hypoxia associated with the tumor microenvironment contribute to this burden by promoting protein misfolding and inhibiting proper glycosylation [32],[33]. As such, the presence of sustained ER stress has been established in many types of cancer and the pathway has been the focus of anticancer drug development [34]-[38]. In accordance with these observations, it is reasonable to consider cancer cells as being more sensitive to this perturbation while nonmalignant cells have a greater capacity to survive during periods of decreased protein translation. Further considering the role of ER stress in C-6-induced cell death, it is interesting to note that multiple signaling arms of the unfolded protein response (UPR) can be activated during periods of ER stress and interplay between these signaling pathways is directly responsible for the activation of either pro-survival or pro-death pathways (reviewed in [39]). The presence of multiple UPR signaling arms and the naturally occurring variation between cell types provides a reasonable explanation for disparities in the magnitude of the ER stress response observed between the cell lines examined. It is therefore possible that C-6 can differentially activate ER stress response pathways depending upon the predisposition of the cell line utilized.

We also demonstrated that C-6 has a considerable effect on mitochondrial morphology and function. TEM images revealed gross morphological changes to the mitochondria of C-6-treated MCF-7 cells; however, this effect was not observed in the normal mammary epithelial cell line examined. We were therefore surprised to find that MCF-10A cells experience metabolic dyshomeostasis and defects in oxidative phosphorylation in a similar manner to three cancer cell lines following C-6 treatment. However, a differential response between the normal epithelial cell line and malignant cell lines was established on the basis of ROS production. Upon treatment with C-6 for 48 hours, MCF-7, T47D, and MDA-MB-231 cells experience significantly higher levels of oxidative stress than the MCF-10A cells. These results demonstrate that C-6 has the ability to affect mitochondrial energy production and suggest that ROS could play a role in the cancer-selective nature of C-6.

Finally, this report details structural modifications made to C-6 and the effects of these scaffold modifications on the small molecule’s potency and cancer selectivity. Using MCF-10A and MCF-7 cells to model the biological activity of the analogs, we found that, with the exception of the *para*-carboxylic acid compound (analog 9), every analog was cytotoxic against the MCF-7 breast cancer cells with an EC_50_ less than 30 μM. In contrast, the cancer-selectivity profiles showed considerable variability. Nearly half of the structural alterations made to the parent molecule resulted in a diminished or complete loss of selectivity for cancer cells. Overall, these data establish that while scaffold modification is most likely to affect C-6’s cancer-specific cytotoxicity, C-6 analogs with excellent selectivity profiles and potencies that surpass the parent molecule can be synthesized. Additional experiments designed to assess mechanistic similarities between the three most cancer-selective analogs and the parent molecule were also conducted. Not unexpectedly, the results of these studies suggested that the analogs affect the same biological pathways as C-6. Based on these results, it is reasonable to consider that C-6 could be amenable to modifications supporting affinity-based target identification studies.

## Conclusions

The collective results of our investigation into C-6’s mechanism of action support a role for the induction of mitochondrial and ER stress and demonstrate that these stressors are a component of C-6’s biological activity. The results of the study also establish that caspase-independent cell death promoted by C-6 occurs concomitantly with the induction of oxidative stress, suggesting a role for oxidative dyshomeostasis in the cancer-selective nature of the small molecule. Finally, C-6 analogs were designed to explore the functional group tolerance of the molecule in cell viability assays. These structure-activity relationship studies revealed that analogs with cancer-selective properties and improved anticancer effects as compared to the parent molecule could be generated and that these analogs retained the same biological activity; these results also inform the design of molecules for fluorophore- and affinity-based target identification studies.

## Additional files

## Electronic supplementary material

Additional file 1: Synthesis of C-6 and C-6 analogs. (PDF 3 MB)

Additional file 2: Densiometric analyses of western blots probing ER stress proteins. Densiometric plots were generated from the data presented in Figure 1D. The analysis was conducted by normalizing each protein band to its respective loading control. (TIFF 1 MB)

Additional file 3: Inhibition of C-6-induced cytoplamic vacuolation by inhibitors of transcription and translation. MCF-7, T47D, and MDA-MB-231 cells were treated with C-6 (30 μM) for 24 hours with or without inhibitors of transcription (actinomycin D, ACTD) or translation (cycloheximide, CHX). Cells were then stained with MitoTracker and live imaging was conducted to assess mitochondrial morphology. (TIFF 11 MB)

Additional file 4: **Mechanistic studies of C-6 analogs.** **(A)** T47D cells were treated with C-6 (or analogs) for 24 hours, stained with MitoTracker, then imaged as live cells to assess mitochondrial morphology. **(B)** T47D cells were treated for 24 hours with either 30 μM C-6 or 30 μM of analog 1, 8, or 12 then CHOP gene expression was measured by real-time PCR. **(C)** Caspase 3/7, 8, and 9 activity was measured in T47D cells following 72 hours of small molecule treatment (each at 30 μM) or 18 hours of staurosporine (1 μM) treatment using the Promega Caspase-Glo assay system. **(D)** Measurement of oxygen consumption rates following pre-treatment of cells with either C-6 or analogs 1, 8, or 12 (each at 30 μM) for 24 hours. **(E)** Measurement of oxidative stress by DCF staining following 48 hours of treatment with 30 μM compound. (TIFF 9 MB)

Below are the links to the authors’ original submitted files for images.

Authors’ original file for figure 1

Authors’ original file for figure 2

Authors’ original file for figure 3

Authors’ original file for figure 4

## Abbreviations

ACTD: actinomycin D
BCA: bicinchoninic acid
CHOP: CCAAT-enhancer-binding protein homologous protein
CHX: cycloheximide
CoA: coenzyme A
DMEM: Dulbecco’s modified Eagle’s medium
DMSO: dimethyl sulfoxide
DTT: dithiothreitol
ECAR: extracellular acidification rate
EGF: epidermal growth factor
ER: endoplasmic reticulum
FBS: fetal bovine serum
FCCP: carbonyl cyanide 4-(trifluoromethoxy)phenylhydrazone
H2DCFDA: 2′,7′-dichlorodihydrofluorescein diacetate
HBSS: Hanks' balanced salt solution
ITS-X: insulin transferrin-selenium-X
JNK: c-Jun kinase
MOX: O-methoxylamine hydrochloride
MSTFA: N-methyl-N-trimethylsilyltrifluoracetamide
Myx: myxothiazol
OCR: oxygen consumption rate
Oligo: oligomycin A
ROS: reactive oxygen species
Rot: rotenone
RT-PCR: real-time polymerase chain reaction
SDS: sodium dodecyl sulfate
TCA: tricarboxylic acid cycle
TEM: transmission electron microscopy
UPR: unfolded protein response
