# Supplementary material for: The small molecule C-6 is selectively cytotoxic against breast cancer cells and its biological action is characterized by mitochondrial defects and endoplasmic reticulum stress
Source: Breast Cancer Res. 2014 Nov 26;16:472. doi: 10.1186/s13058-014-0472-0 (PMC4303206; doi:10.1186/s13058-014-0472-0)
Supplement: Supplementary file 1 — Additional file 1: Synthesis of C-6 and C-6 analogs. (PDF 3 MB) [file 13058_2014_472_MOESM1_ESM.pdf]

## Supporting Information

## Synthesis of C-6 and C-6 Analogs

***The small molecule C-6 is selectively cytotoxic against breast cancer cells and its biological action is characterized by mitochondrial defects and ER stress***

*Rachel M. Vaden[1], Keith M. Gligorich[2], Ranjan Jana[1], Matthew S. Sigman[1], and Bryan E. Welm[2, 3, 4]*

[1]Department of Chemistry, University of Utah, 315 S 1400 E, Salt Lake City, Utah, 84112, USA.

[2]Department of Oncological Sciences, Huntsman Cancer Institute, University of Utah, 2000 Circle of Hope Dr., Salt Lake City, Utah, 84112, USA.

[3]Department of Surgery, Huntsman Cancer Institute, University of Utah, 2000 Circle of Hope Dr., Salt Lake City, Utah, 84112, USA.

[4]Corresponding Author: bryan.welm@hci.utah.edu

**Chemical Synthesis: General Methods**

Tetrahydrofuran (THF), dichloromethane (DCM), diethyl ether, and toluene were used from an alumina column solvent system (Innovative Technology, Inc.). Triethylamine (TEA) was distilled over calcium hydride. Dimethylformamide (DMF) was stored over activated 3Å molecular sieves. Unless otherwise noted all chemicals were purchased from Aldrich, Acros, TCI, EMD, or Mallinckrodt and used without further purification. Unless otherwise stated, all reactions were performed under a nitrogen atmosphere. (–)-sparteine was prepared from (–)-sparteine sulfate pentahydrate (purchased from Acros) according to a previously reported procedure [1]. Pd[(–)-sparteine]$Cl_2$ was synthesized according to a previously reported procedure [2]. [Pd(I*i*Pr)$Cl_2$]$_2$ was synthesized according to a previously reported procedure [3]. $^1$H NMR spectra were obtained at 300 MHz, 400 MHz or 500 MHz using Varian spectrometers. $^1$H chemical shifts are reported in ppm and referenced to $CHCl_3$ at 7.26 ppm, $CD_2Cl_2$ at 5.32 ppm, or acetone-D6 at 2.05 ppm. $^{13}$C NMR spectra were obtained at 75 MHz, 100 MHz or 125 MHz using Varian spectrometers. $^{13}$C chemical shifts are reported in ppm and referenced to $CHCl_3$ at 77.16 ppm, $CD_2Cl_2$ at 53.84 ppm, or acetone-D6 at 29.84 ppm. High resolution mass spectrometry (HRMS) data were obtained on a Waters LCP Premier XE instrument by ESI/TOF. Infrared (IR) spectra were recorded using a Thermo Nicolet FT-IR. All melting points (MP) are uncorrected and recorded on Thomas Hoover Unimelt capillary melting point apparatus. The following abbreviations are used: EtOAc (ethyl acetate), TLC (thin layer chromatography).

**Synthesis of Analog 10 and C-6**

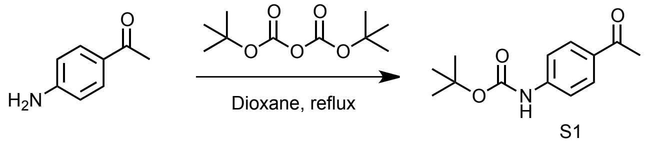


Compound **S1** was prepared following literature procedure and purity assessed using $^1$H NMR [4].

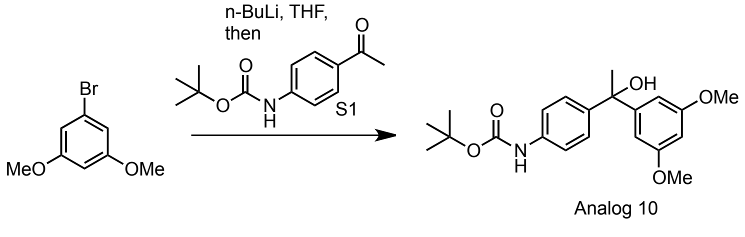


Analog 10

An oven dried round bottom flask fitted with a magnetic stirbar and rubber septum was charged with 1-bromo-3,5-

dimethoxybenze (4.9 g, 22 mmol, 2.5 equiv.). The flask was purged with $N_2$ and 50 mL of THF added via canula. After the solid was allowed to dissolve, the flask was cooled to -78 °C. Dropwise while stirring, previously titrated *n*-BuLi (22 mmol, 2.5 equiv.) was slowly added. The reaction stirred for 1 hour at -78 °C before a solution of **S1** (2.1 g, 8.9 mmol, 1.0 equiv.) in THF (20 mL) was added to the stirring mixture dropwise over 10 minutes. The reaction continued to stir at -78 °C for 2 hours. The reaction was then quenched with $H_2O$ and allowed to slowly warm to room temperature. The product was extracted into EtOAc (2 x 20 mL), washed with brine (2 x 20 mL), dried over $Na_2SO_4$, and concentrated under reduced pressure. The product was purified using silica gel flash column chromatography using a 30:70 mixture of acetone:hexanes. Yield 97%; TLC (30:70 acetone:hexanes) $R_f$=0.17; $^1$H NMR (400 MHz, $CD_2Cl_2$): δ 1.49 (s, 9H), 1.87 (s, 3H), 2.31 (br s, 1H), 3.74 (s, 6H), 6.32 (t, *J* = 2.1 Hz, 1H), 6.55 (d, *J* = 2.2 Hz, 2H), 6.60 (br s, 1H), 7.27-7.34(m, 3H); $^{13}$C NMR (100 MHz, $CDCl_3$): δ 28.4, 30.9, 55.6, 76.14, 80.7, 98.6, 104.5, 118.4, 126.7, 137.8, 142.9, 151.3, 153.1, 161.1; IR: 3331, 2976, 1703, 1707, 1595, 1525, 1502, 1453, 1424, 1405, 1391, 1315, 1236, 1203, 1154, 1105, 1051, 1015, 924, 893, 838, 774, 697 $cm^{-1}$; HRMS $(M+Na)^+$ : calcd. 396.1787, obsvd. 396.1785.

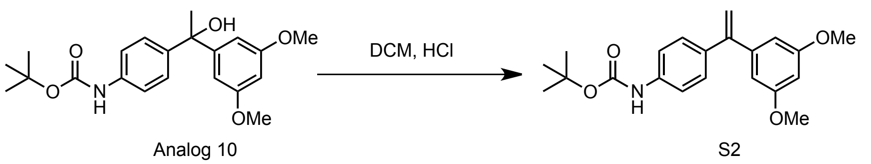


To a flask charged with **Analog 10** (77 mg, 0.2 mmol) in 20 mL of dry DCM, 1 drop of concentrated HCl was added and the reaction stirred at room temperature until TLC indicated consumption of the starting material. The mixture was then dried over $Na_2SO_4$, and concentrated. The final product was used without further purification. Yield >99%; TLC (30:70 acetone:hexanes) $R_f$=0.51; $^1$H NMR (400 MHz, $CDCl_3$): δ 1.50 (s, 9H), 3.72 (s, 6H), 5.36 (d, *J*=11.9 Hz, 1H), 6.40-6.43 (m, 1H), 6.46 (m, 2H), 6.72 (bs, 1H), 7.24-7.32 (m, 4H); $^{13}$C NMR (100 MHz, $CDCl_3$): δ 28.4, 55.3, 80.6, 99.9, 106.6, 113.5, 118.2, 128.8, 135.8, 138.1, 143.8, 149.5, 152.8, 160.5; IR: 3326, 2976, 2838, 2359, 2341, 726, 1652, 1589, 1522, 1455, 1423, 1407, 1392, 1366, 1349, 1314, 1266, 1233, 1204, 1152, 1051, 1027, 988, 926, 901, 839, 768, 690, 667, 647 $cm^{-1}$; HRMS $(M+)^+$ : calcd. 356.1862, obsvd. 356.1869.

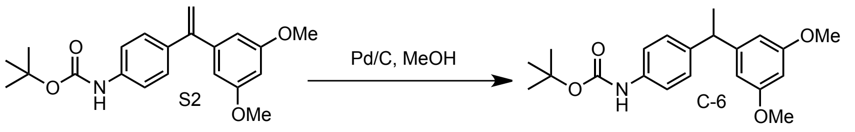


**C-6** was prepared by combining **S2** (0.150 g, 0.422 mmol) with 10% Pd/C (0.089 g, 0.08 mmol, 20 mol%) and 2.0 mL of MeOH. The flask was fitted with a balloon of $H_2$ and the reaction stirred for 12 hours. Then, the reaction mixture was passed through a plug of silica and concentrated. The $^1$H NMR spectrum matched previously reported spectrum [5]. Yield >99%; TLC (30:70 acetone:hexanes) $R_f$=0.51

**Synthesis of Analogs 2 and 3**

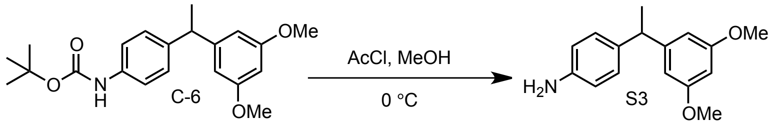


To a flame dried 10-mL round bottom flask equipped with a stir bar under a nitrogen atmosphere, was added 1.0 mL of methanol. The stirred solution was cooled to 0 °C and 560 µL of acetyl chloride (7.8 mmol, 15 equiv.) was added dropwise. The solution was stirred for 10 min. To a flame dried 5 ml round bottom flask, was added 190 mg of C-6 (0.52 mmol, 1.0 equiv.) and 1.0 mL of methanol. The solution of C-6 in methanol was slowly added dropwise to the stirred solution of acetyl chloride in methanol at 0 °C. The red mixture was allowed to warm to room temperature. The mixture was stirred for 3 hours at room temperature and turned yellow. The solvent was subsequently removed

*in vacuo* and approximately 3 mL of DCM was added. Next, hexanes were added until the solution turned cloudy and the solvent was removed *in vacuo* to yield a yellow solid. The solid was washed with $Et_2O$ and the residual solvent was removed by applying a vacuum to the solid. Yield: 90% (139 mg); yellow solid; mp = 68-71 °C; IR 2838, 2590, 1598, 1510, 1460, 1316, 1203, 1155, 1043 $cm^{-1}$; $^1H$ NMR (300 MHz, $CDCl_3$): δ 1.57 (d, *J* = 7.4 Hz, 3H), 3.74 (s, 6H), 4.06 (q, *J* = 7.1 Hz, 1H), 6.29-6.32 (m, 3H), 7.24-7.27 (m, 2H), 7.43 (d, *J* = 8.0 Hz, 2H) 10.39-10.51 (bs, 3H); $^{13}C$ NMR (75 MHz, $CDCl_3$): δ 21.8, 44.7, 55.5, 98.2, 106.1, 123.5, 127.8, 129.3, 147.8, 147.9, 161.0; HRMS (ESI/APCI) m/z $(M–Cl)^+$ calcd.: 258.1493 obsd.: 258.1497.

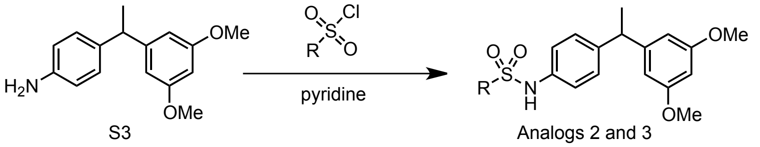


General procedure for the preparation of sulfonamides **Analogs 2** and **3**: In a glass vial, **S3** (0.018 g, 0.072 mmol, 1.0 equiv.) was combined with 0.15 mL of pyridine and the corresponding sulfonyl chloride (1.1 equiv.). After stirring at room temperature for 1 hour, 5 mL of $H_2O$ was added and the product was extracted into EtOAc. The organic layer was washed with 1 M HCl (2 x 5 mL) then brine (1 x 5mL), dried over $Na_2SO_4$, and concentrated. The products were used without further purification.

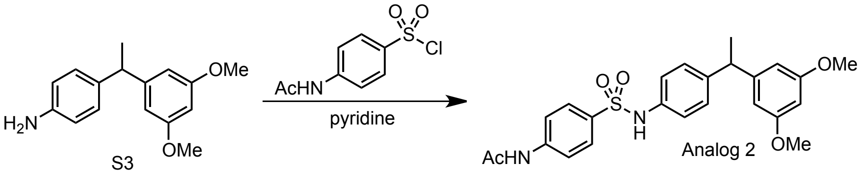


**Analog 2** was synthesized following the general sulfonamide procedure above. Yield 88%; TLC (30:70 EtOAc:hexanes) $R_f$=0.19; $^1H$ NMR (300 MHz, $CDCl_3$): δ 1.53 (d, *J*=7.28 Hz, 3H), 2.17 (s, 3H), 3.74 (s, 6H), 3.94 (q, *J*=7.00 Hz, 1H), 6.29 (s, 3H), 6.84 (s, 1H), 6.95 (d, *J*=8.64 Hz, 2H), 7.07 (d, *J*=8.64 Hz, 2H), 7.52 (d, *J*=8.65 Hz, 2H), 7.60-7.65 (m, 3H); $^{13}C$ NMR (75 MHz, $CDCl_3$): δ 21.7, 24.8, 44.5, 55.4, 97.8, 106.1, 119.2, 122.3, 128.5, 128.6, 133.9, 134.4, 142.2, 143.6, 148.7, 160.8, 168.9; IR: 546, 560, 614, 648, 667, 728, 835, 906, 1018, 1041, 1093, 1153, 1204, 1260, 1315, 1372, 1401, 1428, 1509, 1591, 1678, 2932, 3257 $cm^{-1}$; HRMS $(M+Na)^+$ : calcd. 477.1460, obsvd. 477.1454.

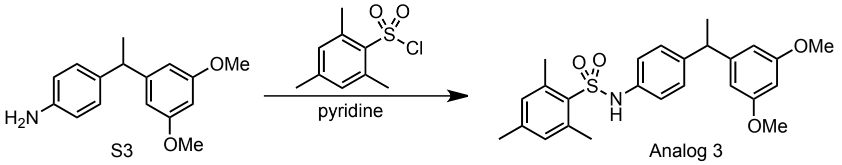


**Analog 3** was synthesized following the general sulfonamide procedure above. Yield 99%. TLC (30:70 EtOAc:hexanes) $R_f$=0.20; $^1H$ NMR (300 MHz, $CDCl_3$): δ 1.53 (d, *J*=7.28 Hz, 3H), 2.27 (s, 3H), 2.57 (s, 6H), 3.72 (s, 6H), 3.97 (q, *J*=7.69 Hz, 1H), 6.28 (s, 3H), 6.84 (d, *J*=8.65, 2H), 6.91 (s, 2H), 7.06 (d, *J*=8.65, 2H); $^{13}C$ NMR (75 MHz, $CDCl_3$): δ 21.0, 21.8, 23.1, 44.4, 55.3, 97.8, 106.1, 121.9, 128.5, 132.1, 133.5, 134.5, 139.4, 142.6, 143.3, 148.6, 160.9; IR: 534, 575, 654, 696, 731, 849, 909, 965, 990, 1018, 1041, 1074, 1146, 1203, 1224, 1289, 1319, 1375, 1427, 1455, 1509, 1593, 2836, 2936, 2965, 3274 $cm^{-1}$; HRMS $(M+Na)^+$ : calcd. 462.1715, obsvd. 452.1718.

**Synthesis of Analog 4**

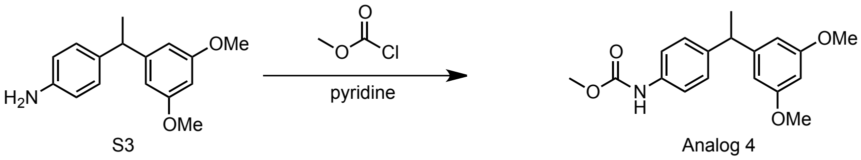


**Analog 4** was prepared according to a previously published procedure [6]. To a flame dried 5-mL round

bottom flask equipped with a stir bar under a nitrogen atmosphere, was added 29 mg of **Analog 4** (0.10 mmol, 1.0 equiv.) and 500 µL of DCM. Next, 19 µL of pyridine (0.23 mmol, 2.3 equiv.) was added and the mixture was stirred at room temperature for 5 min. The mixture was cooled to 0 °C and 9.0 µL of distilled methyl chloroformate (0.12 mmol, 1.2 equiv.) was added. The mixture was stirred at 0 °C for 2 h. Upon warming to room temperature, the mixture was passed through a small column of silica eluting with DCM. The solvent was removed *in vacuo* to yield a light yellow oil. Yield: 70% (22 mg); $R_f$ = 0.39 with 30% acetone/hexanes (silica); yellow oil; IR 3325, 2962, 1731, 1597, 1531, 1459, 1227, 1204, 1154, 1071 $cm^{-1}$; $^1H$ NMR (300 MHz, $CDCl_3$): δ 1.58 (d, *J* = 7.1 Hz, 3H), 3.74 (s, 6H), 3.78 (s, 3H), 4.03 (q, *J* = 7.0 Hz, 1H), 6.29 (t, *J* = 2.2 Hz, 1H), 6.36 (d, *J* = 2.2 Hz, 2H), 6.53-6.59 (bs, 1H), 7.16 (d, *J* = 8.5 Hz, 2H), 7.27-7.29 (m, 2H); $^{13}C$ NMR (75 MHz, $CDCl_3$): δ 22.0, 44.6, 52.6, 55.5, 97.9, 106.1, 128.3, 149.1, 160.9; HRMS (ESI/APCI) m/z $(M+H)^+$ calcd.: 316.1543 obsd.: 316.1550.

**Synthesis of Analogs 1, 11, and 12**

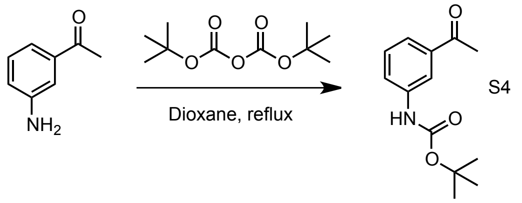


**S4** was prepared following a previously published literature procedure [7] and purity confirmed by $^1H$ NMR comparison [8].

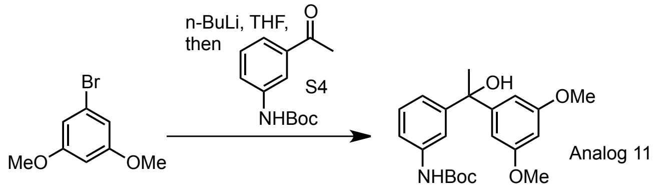


An oven dried round bottom flask fitted with a magnetic stirbar and rubber septum was charged with 1-bromo-3,5-dimethoxybenze (0.5 g, 2.3 mmol, 2.2 equiv.). The flask was purged with $N_2$ and 15 mL of THF added via canula. After the solid was allowed to dissolve, the mixture was cooled to -78 °C. Previously titrated *n*-BuLi (2.3 mmol, 2.2 equiv.) was slowly added dropwise while stirring. The reaction stirred for 1 hour at -78 °C before a solution of **S4** (246 mg, 1.0 mmol, 1.0 equiv.) in THF (10 mL) was added to the stirring mixture dropwise over 10 minutes. The reaction continued to stir at -78 °C for 2 hours. The reaction was then quenched with $H_2O$ and allowed to slowly warm to room temperature. The product was extracted into EtOAc (2 x 10 mL), washed with brine (2 x 10 mL), dried over $Na_2SO_4$, and concentrated under reduced pressure. The product **Analog 11** was purified using silica gel flash column chromatography and 30:70 acetone:hexanes. Yield 61%; TLC (30:70 acetone:hexanes) $R_f$=0.21; $^1H$ NMR (400 MHz, $CDCl_3$): δ 1.50 (s, 9H), 1.90 (s, 3H), 2.25 (s, 1H), 3.75 (s, 6H), 6.34 (t, *J*=4.0, 1H), 6.46 (bs, 1H), 6.57 (d, *J*=2.38, 2H), 7.04-7.07 (m, 1H), 7.21-7.26 (m, 1H), 7.30-7.31 (m, 1H), 7.36-7.38 (m, 1H); $^{13}C$ NMR (100 MHz, $CDCl_3$): δ 28.5, 30.9, 55.4, 76.3, 80.6, 98.8, 104.5, 116.2, 117.4, 120.6, 129.0, 138.4, 148.8, 150.5, 152.9, 160.7; IR: 656, 667, 707, 734, 772, 791, 843, 892, 936, 1046, 1091, 1148, 1202, 1234, 1288, 1366, 1391, 1424, 1455, 1489, 1532, 1592, 1699, 2975, 3334 $cm^{-1}$; HRMS $(M+Na)^+$ : calcd. 396.1787, obsvd. 396.1787.

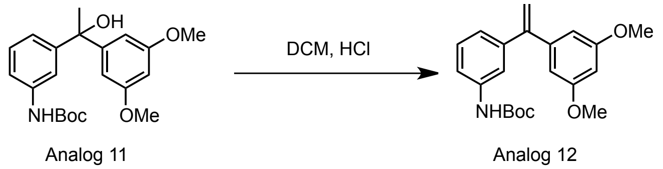


To a flask charged with **Analog 11** (195 mg, 0.05 mmol) in 20 mL of dry DCM, 1 drop of concentrated HCl was added and the reaction stirred at room temperature until TLC indicated consumption of the starting material. The mixture was then dried over $Na_2SO_4$, and concentrated. The final product (**Analog 12**) was used without further purification. Yield 81%; TLC (30:70 acetone:hexanes) $R_f$=0.50; $^1H$ NMR (400 MHz, $CDCl_3$): δ 1.51 (s, 9H), 3.77 (s, 6H), 5.45 (d, *J*=4.0, 2H),

6.43-6.48 (m, 4H), 6.99-7.02 (m, 1H), 7.22 (t, *J*=2 Hz, 1H), 7.25-7.27 (m, 1H), 7.43-7.45 (m, 1H); $^{13}$C NMR (100 MHz, $CDCl_3$): δ 28.5, 55.5, 80.7, 100.1, 106.8, 114.9, 118.2, 118.5, 123.2, 128.9, 138.4, 142.2, 143.6, 149.7, 152.8, 160.6; IR: 667, 706, 729, 795, 858, 1063, 1155, 1204, 1236, 1283, 1349, 1366, 1392, 1422, 1554, 1487, 1533, 1588, 1706, 1727, 2976, 3331 cm$^{-1}$; HRMS $(M+Na)^+$ : calcd. 378.1681, obsvd. 378.1683.

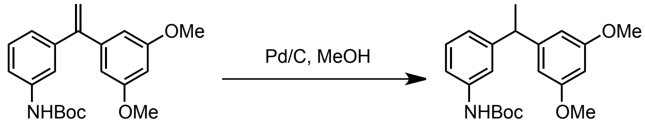

Analog 12 Analog 1

**Analog 1** was prepared by combining **Analog 12** (121 mg, 0.34 mmol) with 10% Pd/C (0.07 mmol, 20 mol%) and 5.0 mL of MeOH. The flask was fitted with a balloon of $H_2$ and the reaction stirred for 12 hours. Then, the reaction mixture was passed through a plug of silica and concentrated. Yield >99%; TLC (30:70 acetone:hexanes) $R_f$=0.50; $^1$H NMR (400 MHz, $CDCl_3$): δ 1.51 (s, 9H), 1.60(d, *J*=7.3 Hz, 3H), 3.76 (s, 6H), 4.05 (q, *J*=7.1 Hz, 1H), 6.31 (t, *J*=2.4, 1H), 6.40 (d, *J*=2.4, 2H), 6.51 (bs, 1H), 6.90-6.92 (m, 1H), 7.17-7.27 (m, 3H); $^{13}$C NMR (100 MHz, $CDCl_3$): δ 21.8, 28.4, 45.1, 55.3, 80.5, 97.8, 106.1, 116.5, 117.9, 122.3, 129.1, 138.5, 147.1, 148.6, 152.8, 160.8; IR: 667, 695, 708, 730, 789, 833, 898, 926, 1051, 1149, 1202, 1234, 1322, 1366, 1391, 1427, 1456, 1490, 1532, 1592, 1700, 2836, 2932, 2969, 3334 cm$^{-1}$; HRMS $(M+Na)^+$ : calcd. 380.1838, obsvd. 380.1841.

**Synthesis of Analogs 6 and 7**

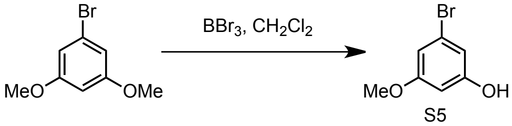

S5

An oven dried 250-mL round bottom flask was equipped with a stir bar and addition funnel. The flask and funnel were purged with $N_2$ and an $N_2$ atmosphere maintained throughout the reaction. 1-bromo 5,5-dimethoxy benzene (1.8 g, 8.5 mmol, 1.0 equiv.) was dissolved in 10 mL of dry DCM and cooled to -78 °C. A 0.5M solution of $BBr_3$ in DCM was prepared in the addition funnel such that there was 1.0 equiv. of $BBr_3$. The solution in the addition funnel was added dropwise over 20 minutes while the reaction stirred. After the reaction stirred at -78 °C for 1 hour, the mixture was allowed to warm to room temperature. The reaction was quenched slowly by adding cold sat. $NaHCO_3$ dropwise until the pH was slightly acidic. The product was extracted into EtOAc (4 x 15 mL) and the organic layer washed with brine. The product was dried over $Na_2SO_4$, concentrated, and purified using silica gel flash column chromatography (30:70 EtOAc:hexanes). Yield 74%; TLC (30:70 EtOAc:hexanes) $R_f$=0.33; $^1$H NMR (400 MHz, $CDCl_3$): δ 6.66 (s, 1H), 6.61 (s, 1H), 6.33 (s, 1H), 4.77 (s, 1H), 3.77 (s, 3H); $^{13}$C NMR (100 MHz, $CDCl_3$): δ 55.7, 100.9, 110.2, 11.6, 123.0, 157.1, 161.5; IR: 601, 672, 813, 825, 860, 968, 991, 1051, 1156, 1196, 1284, 1318, 1451, 1466, 1485, 1590, 2840, 2946, 3389 cm$^{-1}$; HRMS $(M)^+$ : calcd. 202.9708, obsvd. 203.9319.

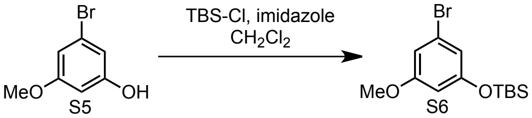


To a mixture of **S5** (1.088 g, 5.3 mmol, 1.0 equiv.) and imidazole (0.9121 g, 13.3 mmol, 2.5 equiv.) in 20 mL of DCM was slowly added *tert*-butyldimethylsilyl chloride (0.9692 g, 6.4 mmol, 1.2 equiv.). The reaction stirred at room temperature until TLC indicated complete consumption of starting material. The reaction was quenched with water, extracted into EtOAc, washed with water and brine, then dried over $Na_2SO_4$ and concentrated. The product was purified with flash chromatography (30:70 EtOAc:hexanes) on silica gel to afford S25. Yield >99%; TLC (30:70 EtOAc:Hexanes) $R_f$=0.80; $^1$H NMR (300 MHz, $CDCl_3$): δ 0.20 (s, 6H), 0.97 (s, 9H), 6.76 (s, 3H), 6.30-6.33 (m, 1H), 6.59-6.62 (m, 1H), 6.67-6.69 (m, 1H); $^{13}$C NMR (75 MHz, $CDCl_3$): δ -4.3, 18.3, 26.1, 55.6, 105.7, 110.5, 116.3, 122.7, 157.6, 161.2; IR: 551, 580, 675, 741, 779, 808, 836, 938, 977, 993, 1051, 1156, 1193, 1229, 1253, 1288, 1315, 1362, 139-, 1424, 1441, 1566, 1592, 2857, 2929, 2955 cm$^{-1}$; HRMS $(M)^+$ : calcd. 317.0572, obsvd. 317.0571.

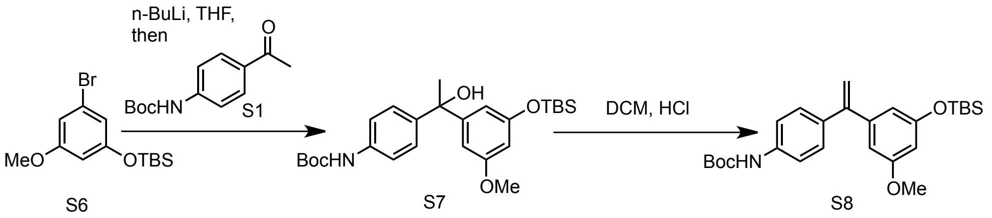


An oven dried round bottom flask fitted with a magnetic stirbar and rubber septum was charged with **S6** (1.4 g, 4.4 mmol, 3.0 equiv.). The flask was purged with $N_2$ and 20 mL of THF added via canula. After the solid was allowed to dissolve, the flask was cooled to -78 °C. Dropwise while stirring, previously titrated *n*-BuLi (4.4 mmol, 1.0 equiv.) was slowly added. The reaction stirred for 1 hour at -78 °C before a solution of **S1** (348 mg, 1.48 mmol, 1.0 equiv.) in THF (10 mL) was added to the stirring mixture dropwise over 10 minutes. The reaction continued to stir at -78 °C for 2 hours. The reaction was then quenched with $H_2O$ and allowed to slowly warm to room temperature. The product was extracted into EtOAc (2 x 20 mL), washed with brine (2 x 20 mL), dried over $Na_2SO_4$, and concentrated under reduced pressure. The product was purified using silica gel flash column chromatography and 30:70 acetone:hexanes. TLC (30:70 acetone:hexanes) $R_f$=0.45. A solution of **S7** in 20 mL of DCM was stirred with a magnetic stir bar. One drop of concentrated HCl was added to the mixture and the reaction stirred for 18 hours at room temperature. The solvent was then removed under reduced pressure and the **S8** was carried directly to the next step.

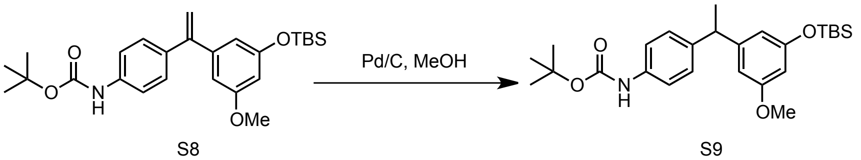


**S9** was prepared by combining **S8** (125 mg, 0.27 mmol) with 10% Pd/C (0.05 mmol, 20 mol%) and 2.0 mL of MeOH. The flask was fitted with a balloon of $H_2$ and the reaction stirred for 12 hours. Then, the reaction mixture was passed through a plug of silica and concentrated. Yield >99%; TLC (30:70 EtOAc:Hexanes) $R_f$=0.62; $^1$H NMR (300 MHz, $CDCl_3$): δ 0.16 (s, 6H), 0.97 (s, 9H), 1.51 (s, 9H), 1.56 (d, *J*=7.13 Hz, 3H), 3.72 (s, 3H), 3.99 (q, *J*=7.26 Hz), 6.21-6.38 (m, 3H), 6.51 (s, 1H), 7.10-7.14 (m, 1H), 7.23-7.29 (m, 2H); $^{13}$C NMR (75 MHz): δ -4.2, 18.4, 21.9, 25.8, 28.5, 44.2, 55.3, 80.5, 103.7, 106.9, 112.7, 118.7, 128.1, 136.4, 141.0, 148.8, 152.9, 156.7, 160.6

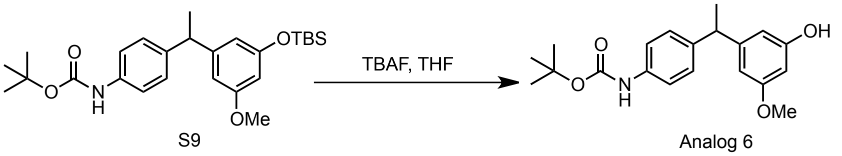


To a flask containing **S9** (0.4126 g, 0.91 mmol, 1.0 equiv.) in 10 mL of THF was added TBAF (1.09 mL of a 1M solution, 1.09 mmol, 1.2 equiv.). The reaction stirred at room temperature until TLC indicated complete consumption of starting material. The reaction was quenched with water, extracted into EtOAc (2 x 10 mL), washed with water (10 mL) and brine, dried over $Na_2SO_4$, and concentrated in vacuo. The product was purified with flash chromatography (30:70 EtOAc:hexanes) on silica gel to afford **Analog 6**. Yield >99%; TLC (30:70 EtOAc:Hexanes) $R_f$=0.62; $^1$H NMR (400 MHz, $CDCl_3$): δ 1.49-1.55 (m, 12H), 3.70 (s, 3H), 3.96 (q, *J*=7.25 Hz, 1H), 5.78 (s, 1H), 6.24 (s, 2H), 6.34 (m, 1H), 6.54 (s, 1H), 7.10 (d, *J*=8.61 Hz, 2H), 7.21-7.24 (m, 2H); $^{13}$C NMR (100 MHz, $CDCl_3$): δ 21.8, 28.4, 44.2, 55.3, 80.7, 98.9, 106.3, 107.4, 119.1, 128.2, 136.2, 141.0, 149.3, 153.3, 156.9, 160.8; IR: 695, 737, 773, 833, 904, 968, 995, 1016, 1048, 1149, 1191, 1235, 1313, 1367, 1391, 1410, 1452, 1501, 1519, 1594, 1612, 1691, 1725, 2973, 3324 $cm^{-1}$; HRMS $(M+K)^+$ : calcd. 382.1421, obsvd. 382.1419.

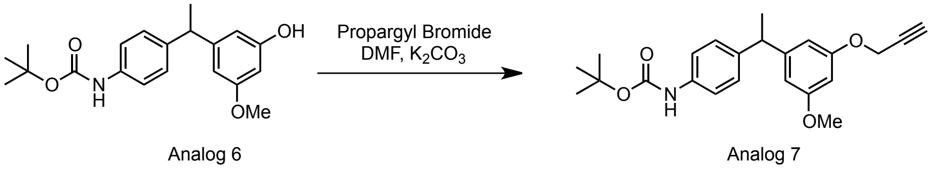


A mixture of **Analog 6** (0.0182 g, 0.53 mmol, 1.0 equiv.), propargyl bromide (0.0095 g, 0.79 mmol, 1.5 equiv.), and $K_2CO_3$ (0.0088 g, 0.63 mmol, 1.2 equiv) in DMF (0.5 mL) was heated at 45 °C for 12 hours. The reaction was allowed to cool to room temperature then quenched with water and extracted into EtOAc. The organic layer was washed twice with water (1 mL) then dried over $Na_2SO_4$ and concentrated. The product was purified with flash chromatography (30:70 EtOAc:hexanes) on silica gel to afford **Analog 7**. Yield 82%; TLC (30:70 EtOAc:hexanes) $R_f$=0.41; $^1$H NMR (400 MHz, $CDCl_3$): δ 1.51 (s, 9H), 1.58 (d, *J*=7.14, 3H), 2.50 (t, *J*=2.4Hz, 1H), 3.74 (s, 3H), 4.02 (q, *J*=7.24, 1H), 4.63 (s, 2H), 6.36-6.43 (m, 4H), 7.13, 7.25 (d, *J*=8.27 Hz, 2H), 7.25 (d, *J*=8.27 Hz, 2H); $^{13}$C NMR (100 MHz, $CDCl_3$): δ 21.8, 28.5, 44.4, 55.4, 55.9, 75.6, 78.7, 98.8, 106.8, 107.0, 118.8, 128.2, 136.5, 140.8, 149.2, 158.8, 160.8; IR: 553, 668, 695, 734, 772, 833, 900, 941, 1015, 1049, 1074, 1148, 1193, 1229, 1263, 1311, 1367, 1392, 1409, 1435, 1456, 1520, 1558, 1592, 1646, 1652, 1684, 1700, 1716, 2926, 2967, 3305 $cm^{-1}$; HRMS $(M+Na)^+$ : calcd. 404.1838, obsvd. 404.1823.

**Synthesis of Analog 8**

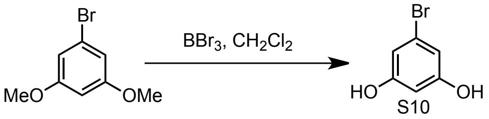


**S10** was prepared following a previously published literature procedure [9].

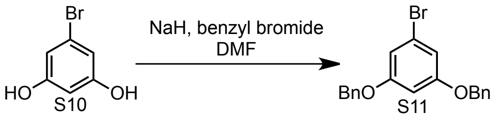


NaH (60% in mineral oil, 33mmol) was added to 50 mL of DMF and stirred under an $N_2$ atmosphere. The reaction was cooled to 0 °C and **S10** (3.1 g, 16.6 mmol, 1.0 equiv.) in 5 mL of DMF was added to the stirring mixture. The reaction stirred for 20 minutes. To the mixture was added benzyl bromide (33 mmol, 2.0 equiv.). The reaction was allowed to warm to room temperature and stirring continued for 3 hours. The reaction was quenched with saturated ammonium chloride solution and diluted with diethyl ether. The organic layer was dried and concentrated and the crude product purified by silica gel flash column chromatography (5:95 EtOAc:hexanes). Yield 91%; TLC (5:95 EtOAc:Hexanes) $R_f$=0.33; MP=58-60 °C; $^1$H NMR (300 MHz, $CDCl_3$): δ 5.00 (s, 4H), 6.54 (t, *J*=2.33 Hz, 1H), 6.77 (d, *J*=2.19 Hz, 2H), 7.32-7.41 (m, 10H); $^{13}$C NMR (75 MHz, $CDCl_3$): δ 70.4, 101.5, 111.2, 123.1, 127.7, 128.3, 128.8, 136.4, 160.5; IR: 630, 674, 695, 733, 816, 907, 1026, 1051, 1080, 1151, 1212, 1278, 1328, 1377, 1436, 1497, 1573, 1594, 2870, 3031 $cm^{-1}$; HRMS $(M)^+$ : calcd. 369.0490, obsvd. 369.0492.

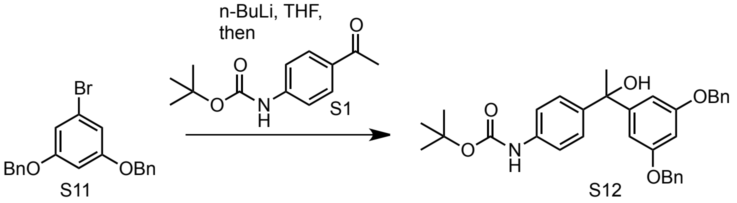


An oven dried round bottom flask fitted with a magnetic stirbar and rubber septum was charged with **S11** (3.9 mmol, 3.3 equiv.). The flask was purged with $N_2$ and 10 mL of THF added via canula. After the solid was allowed to dissolve, the flask was cooled to -78 °C. Previously titrated *n*-BuLi (3.9 mmol, 3.3 equiv.) was slowly added dropwise while stirring. The reaction stirred for 1 hour at -78 °C before a solution of **S1** (310 mg, 1.3 mmol, 1.0 equiv.) in THF (10 mL) was added to the stirring mixture dropwise over 10 minutes. The reaction continued to stir at -78 °C for 2 hours. The reaction was then quenched with $H_2O$ and allowed to slowly warm to room temperature. The product was extracted into EtOAc (2 x 10 mL), washed with brine

(2 x 10 mL), dried over $Na_2SO_4$, and concentrated under reduced pressure. The product **S12** was purified using silica gel flash column chromatography and 30:70 acetone:hexanes. Yield 51%; TLC (20:80 EtOAc:hexanes) $R_f$=0.11; $^1$H NMR (300 MHz, $CDCl_3$): δ 1.49 (s, 9H), 1.83 (s, 3H), 2.43 (s, 1H), 4.93 (s, 4H), 6.47 (s, 1H), 6.63-6.66 (m, 3H), 7.20-7.37 (m, 14H); $^{13}$C NMR (75 MHz, $CDCl_3$): δ 28.5, 30.9, 70.2, 76.1, 80.7, 100.4, 105.6, 118.3, 126.6, 127.8, 128.1, 128.7, 136.9, 137.3, 142.4, 150.8, 152.9, 159.8; IR: 647, 667, 696, 732, 836, 907, 1027, 1050, 1105, 1151, 1233, 1289, 1315, 1367, 1404, 1436, 1453, 1498, 1520, 1592, 1652, 1700, 2929, 2975, 3032, 3331 cm$^{-1}$; HRMS (M+Na)$^+$ : calcd. 548.2413, obsvd. 548.2411.

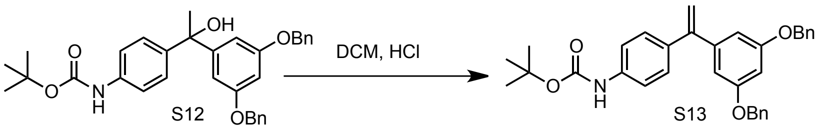


To a flask charged with **S12** (353 mg, 0.7 mmol) in 20 mL of dry DCM, 1 drop of concentrated HCl was added and the reaction stirred at room temperature until TLC indicated consumption of the starting material. The mixture was then dried over $Na_2SO_4$, and concentrated. The final product **S13** was used without further purification. Yield >99%; TLC (20:80 EtOAc:hexanes) $R_f$=0.32; MP: 100-102 °C; $^1$H NMR (300 MHz, $CDCl_3$): δ 1.51 (s, 9H), 4.98 (s, 4H), 5.37 (d, *J*=5.35, 2H), 6.56-6.60 (m, 4H), 7.21-7.40 (m, 14 H); $^{13}$C NMR (75 MHz, $CDCl_3$): δ 28.4, 70.2, 80.7, 101.6, 107.9, 113.8, 118.2, 127.7, 128.1, 128.6, 129.0, 135.9, 136.9, 138.1, 143.7, 149.3, 152.8, 159.7; IR: 594, 668, 696, 731, 839, 905, 1027, 1051, 1080, 1148, 1228, 1312, 1343, 1367, 1391, 1404, 1432, 1453, 1497, 1518, 1582, 1705, 2929, 2975, 3032, 3332 cm$^{-1}$; HRMS (M+Na)$^+$ : calcd. 530.2307, obsvd. 530.2298.

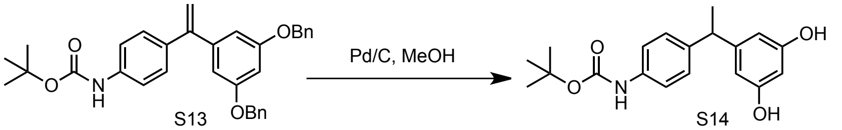


Compound **S14** was prepared by combining **S13** (210 mg, 0.4 mmol) with 10% Pd/C (0.08 mmol, 20 mol%) and 2.0 mL of MeOH. The flask was fitted with a balloon of $H_2$ and the reaction stirred for 12 hours. Then, the reaction mixture was passed through a plug of silica and concentrated. TLC (30:70 EtOAc:Hexanes) $R_f$=0.17; MP=112-114 °C; $^1$H NMR (300 MHz, Acetone-$d_6$): δ 1.47 (s, 9H), 1.52 (d, *J*=7.3 Hz, 3H), 3.94 (q, *J*=7.1, 1H), 6.15-6.16 (m, 1H), 6.21 (m, 2H), 7.15 (d, *J*=8.6 Hz, 2H), 7.45 (d, *J*=8.6 Hz, 2H), 8.0 (s, 2H), 8.27 (bs, 1H); $^{13}$C NMR (75 MHz, Acetone-$d_6$): δ 22.1, 28.5, 44.8, 79.8, 101.2, 107.0, 119.1, 128.5, 138.5, 141.3, 150.2, 153.8, 159.3; IR: 651, 667, 695, 773, 835, 921, 990, 1017, 1064, 1098, 1156, 1249, 1312, 1367, 1392, 1411, 1450, 1520, 1599, 1694, 2874, 2931, 2975, 3325 cm$^{-1}$; HRMS (M+Na)$^+$ : calcd. 352.1525, obsvd. 352.1526.

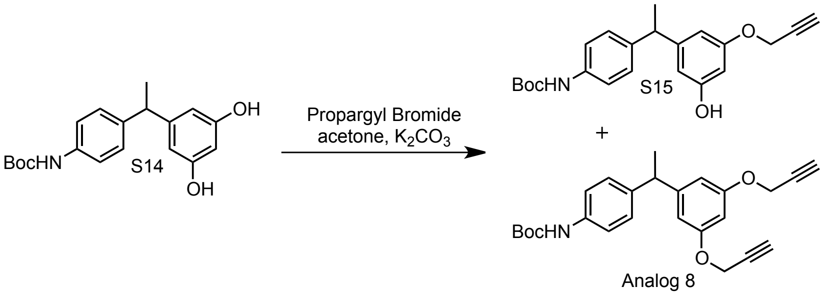


Propargyl bromide (26 mg, 0.2 mmol, 1.0 equiv.) was added to a stirring mixture of **S14** (73 mg, 0.2 mmol, 1.0 equiv.), acetone (15 mL) and $K_2CO_3$ (183 mg, 1.3 mmol, 6.0 equiv.). The reacted was heated to reflux and stirred for 16 hours. Then, the mixture was allowed to cool to room temperature, filtered, and concentrated. The products **S15** and **Analog 8** were purified by silica gel flash column chromatography (30:70 EtOAc:hexanes). **S15**: Yield 32%; TLC (30:70 EtOAc:hexanes) $R_f$=0.31; $^1$H NMR (400 MHz, $CDCl_3$): δ 1.51 (s, 9H), 1.55 (d, *J*=7.1 Hz, 3H), 2.49-2.50 (m, 1H), 3.98 (q, *J*=7.1 Hz, 1H), 4.60 (d, *J*=2.3 Hz, 2H), 5.17 (bs, 1H), 6.27-6.31 (m, 2H), 6.42-6.45 (m, 2H), 7.10-7.16 (m, 2H), 7.23-7.26 (m, 2H); $^{13}$C NMR (125 MHz, $CDCl_3$): δ 21.7, 28.5, 44.2, 55.94, 75.7, 78.6, 80.72, 99.9, 107.2, 108.2, 119.0, 128.2, 136.4, 140.8, 149.5, 156.7, 158.9, 173.2; IR: 648, 667, 695, 731, 774, 834, 906, 963, 1016, 1044, 1155, 1236, 1314, 1367, 1392, 1410, 1455, 1521, 1595, 1695, 2928, 2972, 3289 cm$^{-1}$; HRMS (M+Na)$^+$ : calcd.

390.1681, obsvd. 390.1679; **Analog 8**: Yield 43%; TLC (30:70 EtOAc:hexanes) $R_f$=0.48; $^1$H NMR (500 MHz, $CDCl_3$): δ 1.64 (s, 9H), 1.71 (d, *J*=7.3 Hz, 3H), 1.56 (t, *J*=2.4 Hz, 2H), 4.17 (q, *J*=6.8 Hz, 1H), 4.75 (d, *J*=1.2 Hz, 4H), 6.56-6.61 (m, 4H), 7.26-7.28 (m, 2H), 7.38-7.41 (m, 2H); $^{13}$C NMR (100 MHz, $CDCl_3$): δ 21.8, 28.5, 44.4, 56.0, 75.7, 78.6, 80.6, 99.6, 107.8, 118.8, 128.2, 136.5, 140.7, 149.3, 158.7, 173.2; IR: 667, 835, 1015, 1051, 1157, 1233, 1288, 1312, 1367, 1392, 1410, 1455, 1521, 1594, 1718, 2929, 2973, 3288 cm$^{-1}$; HRMS $(M+Na)^+$ : calcd. 428.1838, obsvd. 428.1839.

### Synthesis of Analog 5

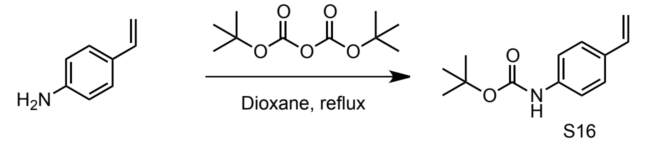


Compound **S16** was prepared following a previously published literature procedure and purity confirmed by $^1$H NMR comparison [10].

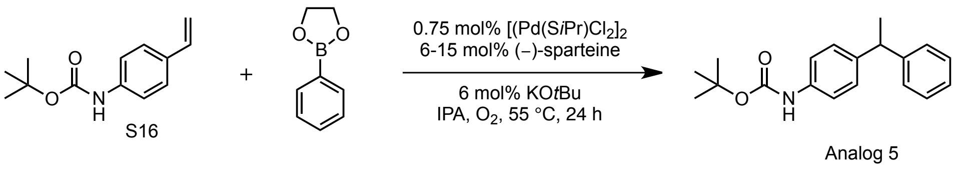


**Analog 5** was prepared following a previously published literature procedure and purity confirmed by $^1$H NMR comparison [5].

### Synthesis of Analog 9

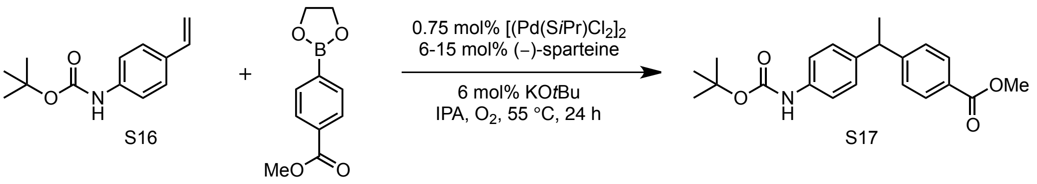


**S17** was prepared according to a previously published procedure [6]. To a flame dried 500 mL Schlenk flask equipped with a stir bar and a water condenser under a nitrogen atmosphere, was added 25.5 mg of $[Pd(I\textit{i}Pr)Cl_2]_2$ (0.0230 mmol, 0.00750 equiv.), 60.0 mL of isopropanol, and 106 mg of (–)-sparteine (0.450 mmol, 0.150 equiv.). A three-way joint fitted with a balloon of $O_2$ was installed on the condenser. The flask was evacuated *via* water aspiration and refilled with oxygen three times and the mixture was stirred vigorously for ca. 20 minutes at room temperature under $O_2$. To the stirred mixture was added, 658 mg of **S16** (3.00 mmol, 1.00 equiv.), 1.85 g of the boronic ester (9.00 mmol, 3.00 equiv.), which was synthesized according to a previously reported procedure, and 20.2 mg of KO*t*Bu (0.180 mmol, 0.0600 equiv.) [11]. The flask was evacuated *via* water aspiration and refilled with oxygen three times and the mixture was stirred vigorously for ca. 10 minutes at room temperature. The mixture was then heated to 55 °C and stirred vigorously for 20 hours. However, within 4 hours the reaction mixture had turned black. Upon cooling to room temperature, the mixture was transferred to a 250 mL round bottom flask and the solvent was removed *in vacuo*. Next, 40 mL of $H_2O$ were added and the mixture was transferred to a separatory funnel. The aqueous layer was extracted three times with 60 mL of 50% EtOAc/hexanes. All the organic extracts were combined, washed with 150 mL of brine, dried with $MgSO_4$, filtered, and concentrated *in vacuo* to yield a yellow solid. The mixture was purified by silica gel flash chromatography eluting with 8-10% acetone/hexanes. The product was isolated as an approximate 1:1 mixture with an alkene containing byproduct (284 mg total), which was inseparable by chromatography. Therefore, to a 25 mL round bottom flask equipped with a stir bar, were added 2.70 mL of $H_2O$, 553 mg of AD-mix-α (Aldrich), and 1.00 mL of *t*BuOH. The orange mixture was stirred for 10

minutes and was then cooled to 0 °C. The mixture of the desired product and alkene contaminant was dissolved in 1.00 mL of *t*BuOH and was added to the stirred solution of AD-mix-α. The orange mixture was stirred for 16 hours and then 60.0 mg of $Na_2SO_3$ was added. The mixture was stirred for 2 hours and 20 mL of $H_2O$ was added. The aqueous solution was extracted three times with 20 mL of EtOAc. The organic extracts were combined, washed with 60 mL of brine, dried with $MgSO_4$, filtered, and the solvent was removed *in vacuo* to yield a yellow oil. The product was purified by silica gel flash chromatography eluting with 10% acetone/hexanes. Yield: 8% (81 mg); $R_f$ = 0.36 with 20% acetone/hexanes; clear oil; IR 3344, 2973, 2360, 1720, 1610, 1436, 1280, 1159, 1113, 1054; $^1$H NMR (400 MHz, $CDCl_3$): δ 1.54 (s, 9H), 1.63 (d, *J* = 7.3 Hz, 3H), 3.90 (s, 3H), 4.16 (q, *J* = 7.1 Hz, 1H), 6.40-6.42 (bs, 1H) 7.12 (d, *J* = 8.4 Hz, 2H), 7.26-7.29 (m, 4H), 7.94 (d, *J* = 8.4 Hz, 2H); $^{13}$C NMR (100 MHz, $CDCl_3$): δ 21.9, 28.6, 44.4, 52.2, 119.0, 127.8, 128.3, 129.9, 136.8, 140.3, 152.1, 153.0, 167.3; HRMS (ESI/APCI) m/z $(M+Na)^+$ calcd.: 378.1681 obsd.: 378.1672 [6].

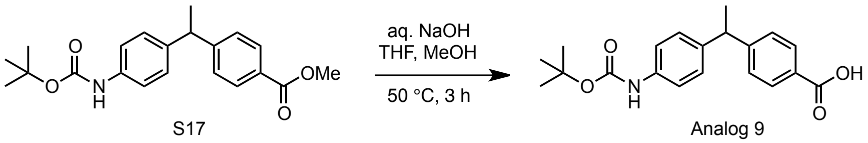


**Analog 9** was prepared according to a previously published procedure [6]. To a flame dried 10 mL thick-wall glass pressure vessel equipped with a stir bar under a nitrogen atmosphere, was added 70 mg of **S17** (0.20 mmol, 1.0 equiv.), 400 µL of THF, and 400 µL of methanol. Next, 600 µL of a 1.0 M solution of NaOH in $H_2O$ (0.60 mmol, 3.0 equiv.) was added and the vessel was sealed. The mixture was heated to 50 °C and was stirred for 3 hours. Upon cooling to room temperature, the mixture was transferred to a 10 mL round bottom flask and was concentrated *in vacuo* until approximately 600 µL remained. Next, 2.0 mL of $H_2O$ was added and 1.0 M aqueous HCl was added until the pH was 1-2. The aqueous solution was extracted three times with 10 mL of EtOAc. The organic extracts were combined, washed with 30 mL of brine, dried with $MgSO_4$, filtered, and concentrated *in vacuo* to yield a white solid. Yield: 94% (64 mg); $R_f$ = 0.35 with 40% acetone/hexanes; white solid; mp = 195-197 °C; IR 3380, 2979, 2930, 1678, 1610, 1525, 1410, 1283, 1234, 1168, 1017; $^1$H NMR (300 MHz, $CD_3OD$): δ 1.47 (s, 9H), 1.58 (d, *J* = 7.1 Hz, 3H), 4.14 (q, *J* = 7.3 Hz, 1H), 7.10 (d, *J* = 8.2 Hz, 2H), 7.26-7.30 (m, 4H), 7.89 (d, *J* = 8.4 Hz, 2H); $^{13}$C NMR (75 MHz, $CD_3OD$): δ 22.2, 28.9, 45.6, 120.2, 128.8, 129.0, 131.0, 138.9, 141.4, 153.8; HRMS (ESI/APCI) m/z $(M+Na)^+$ calcd.: 364.1525 obsd.: 364.1517 [6].

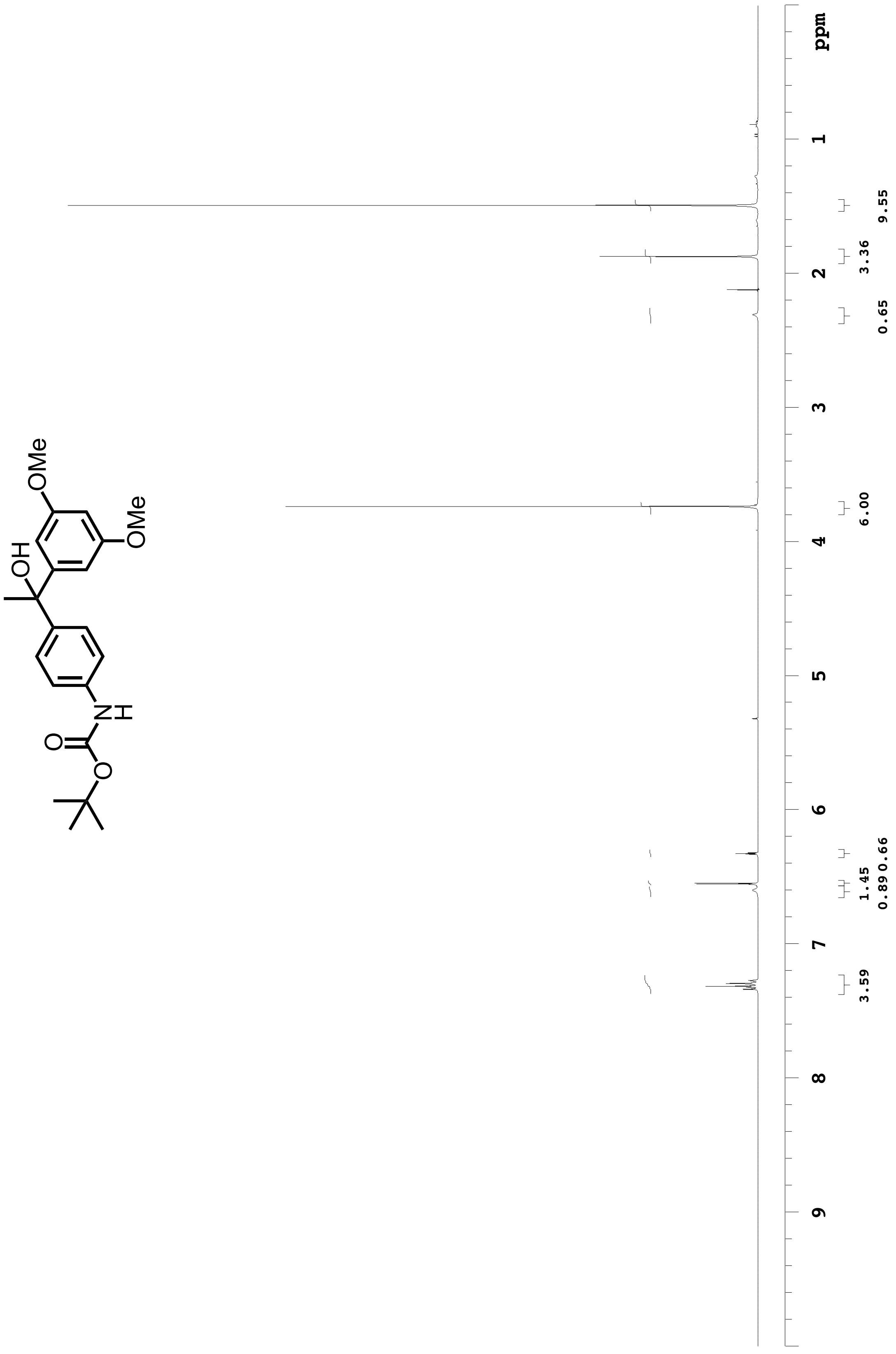

OMe
OMe
OH
NH
O
O
ppm
1
2
3
4
5
6
7
8
9
9.55
3.36
0.65
6.00
1.45
0.89
0.66
3.59

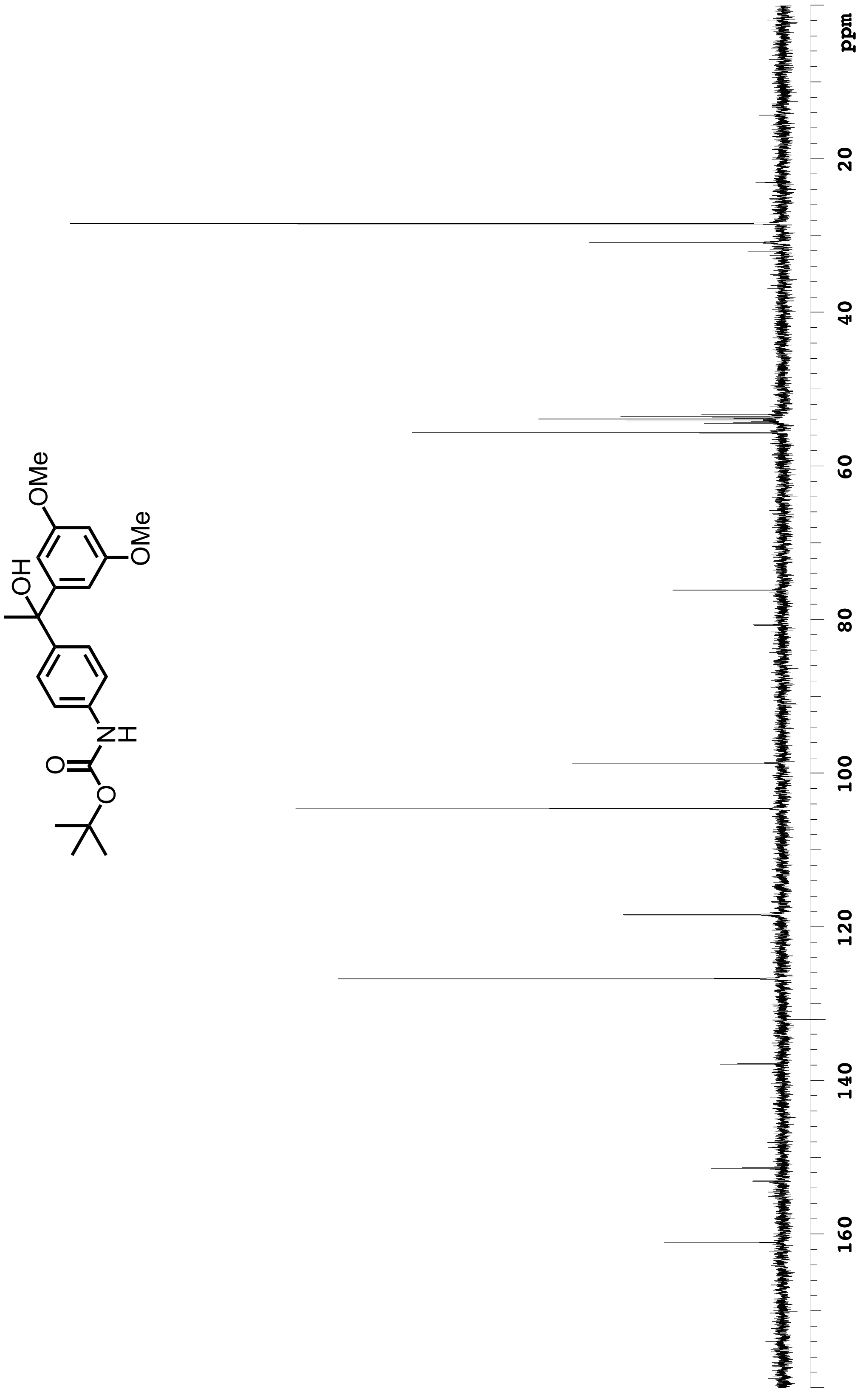
OMe
OMe
OH
N
H
O
O
ppm
20
40
60
80
100
120
140
160

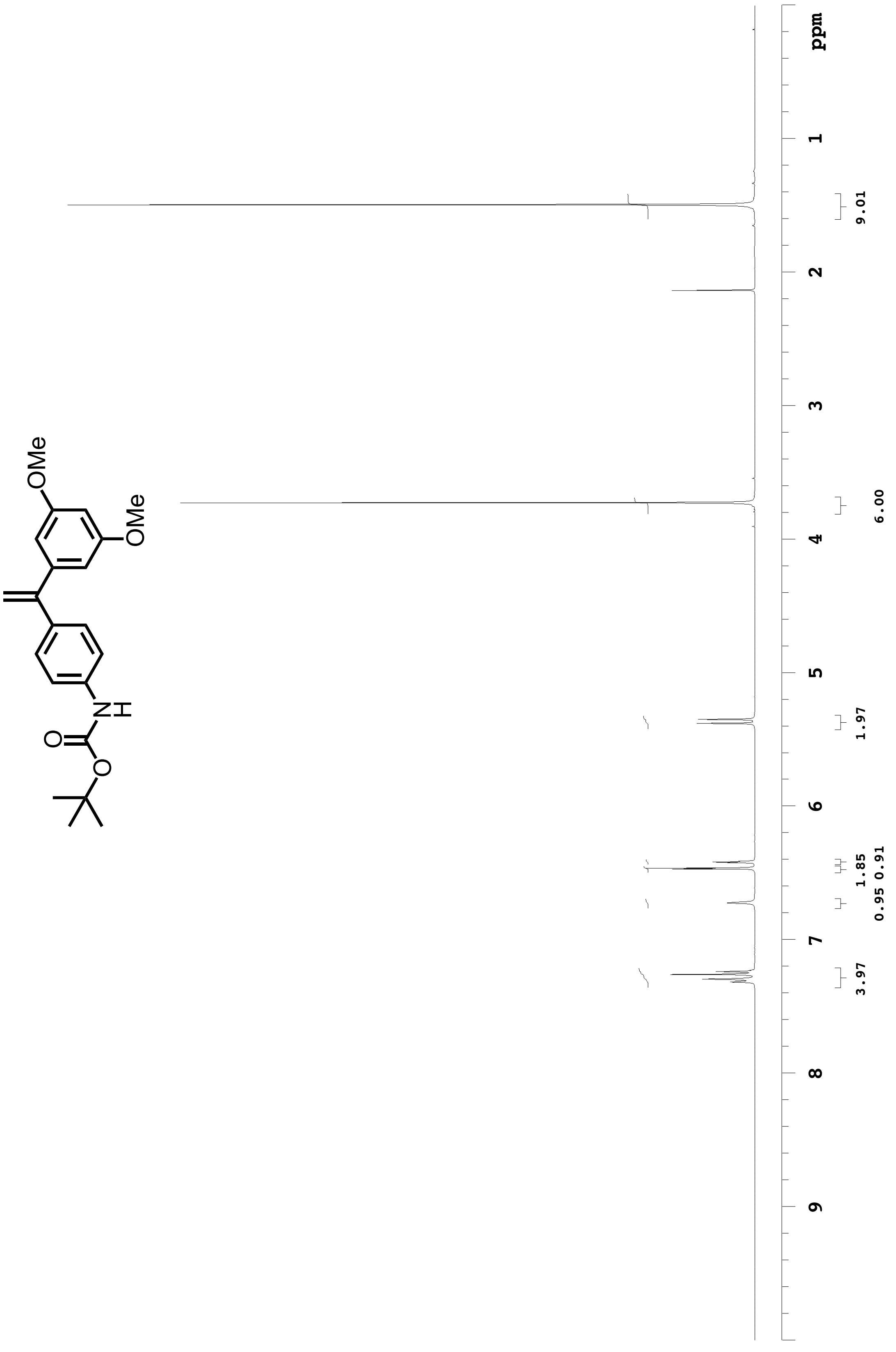
OMe
OMe
NH
O
O
ppm
1
2
3
4
5
6
7
8
9
9.01
6.00
1.97
1.85
0.95 0.91
3.97

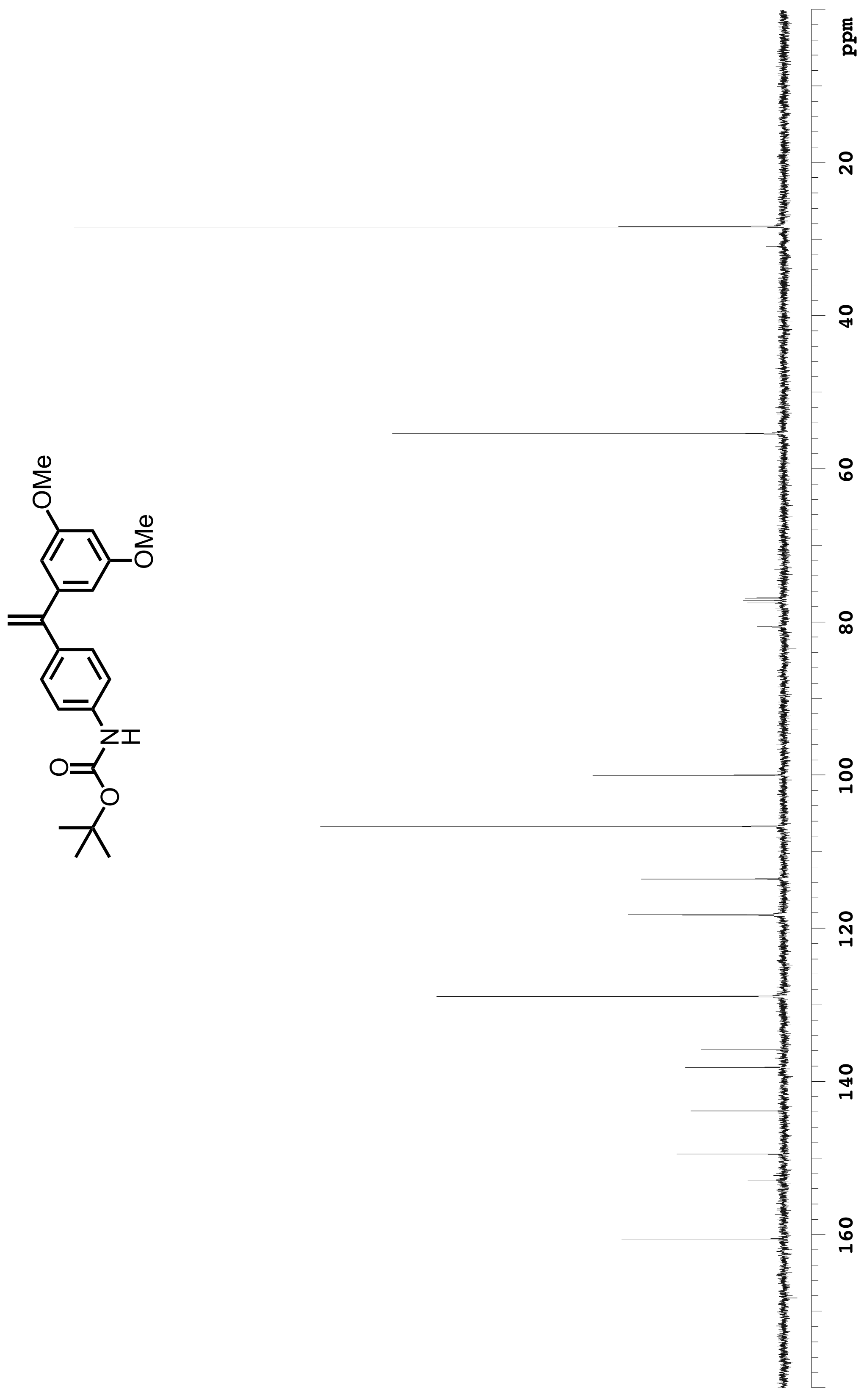
OMe
OMe
NH
O
O
ppm
20
40
60
80
100
120
140
160

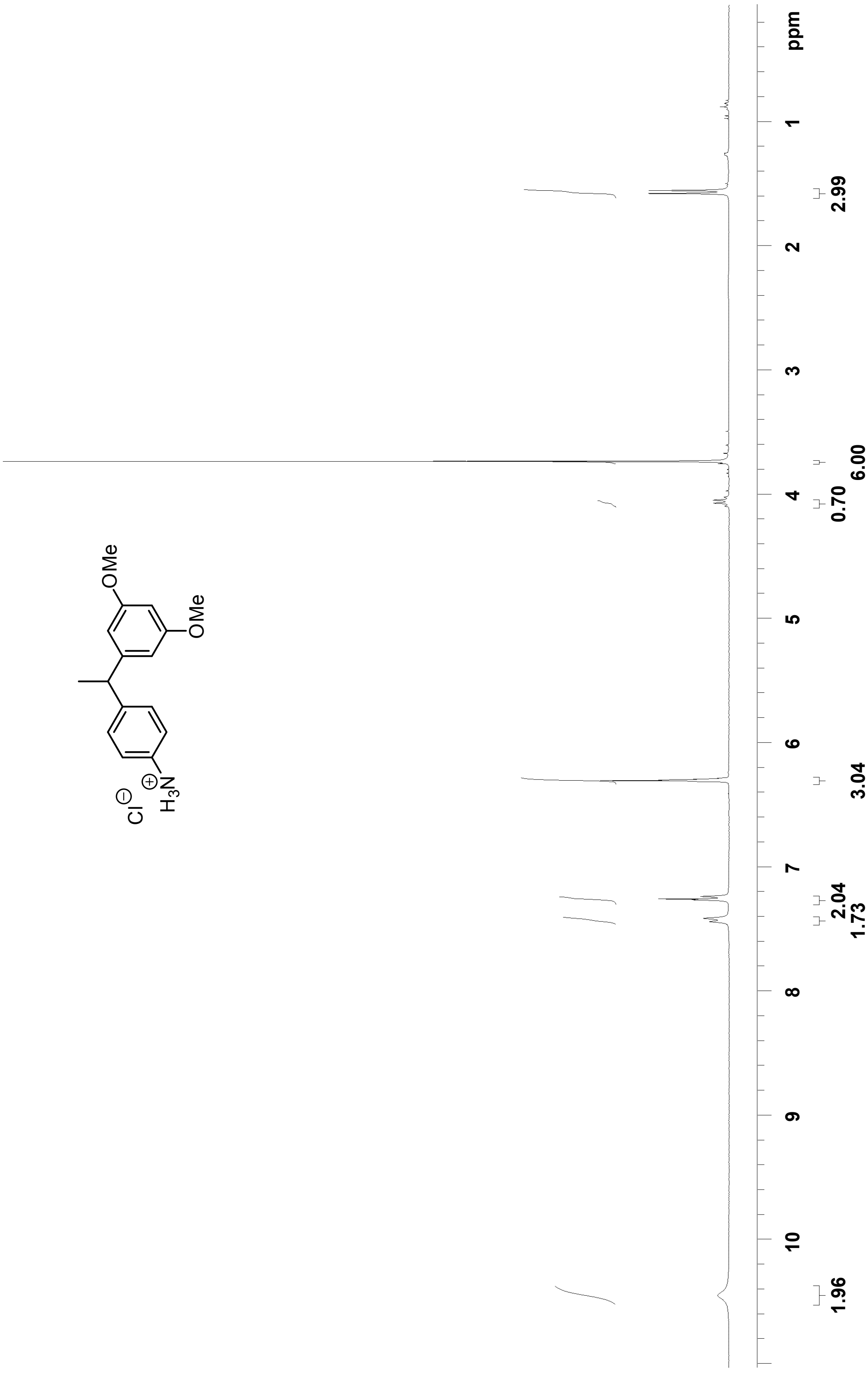

OMe
OMe
$Cl^{\ominus}$
$H_3\overset{\oplus}{N}$
ppm
1
2
3
4
5
6
7
8
9
10
2.99
0.70
6.00
3.04
2.04
1.73
1.96

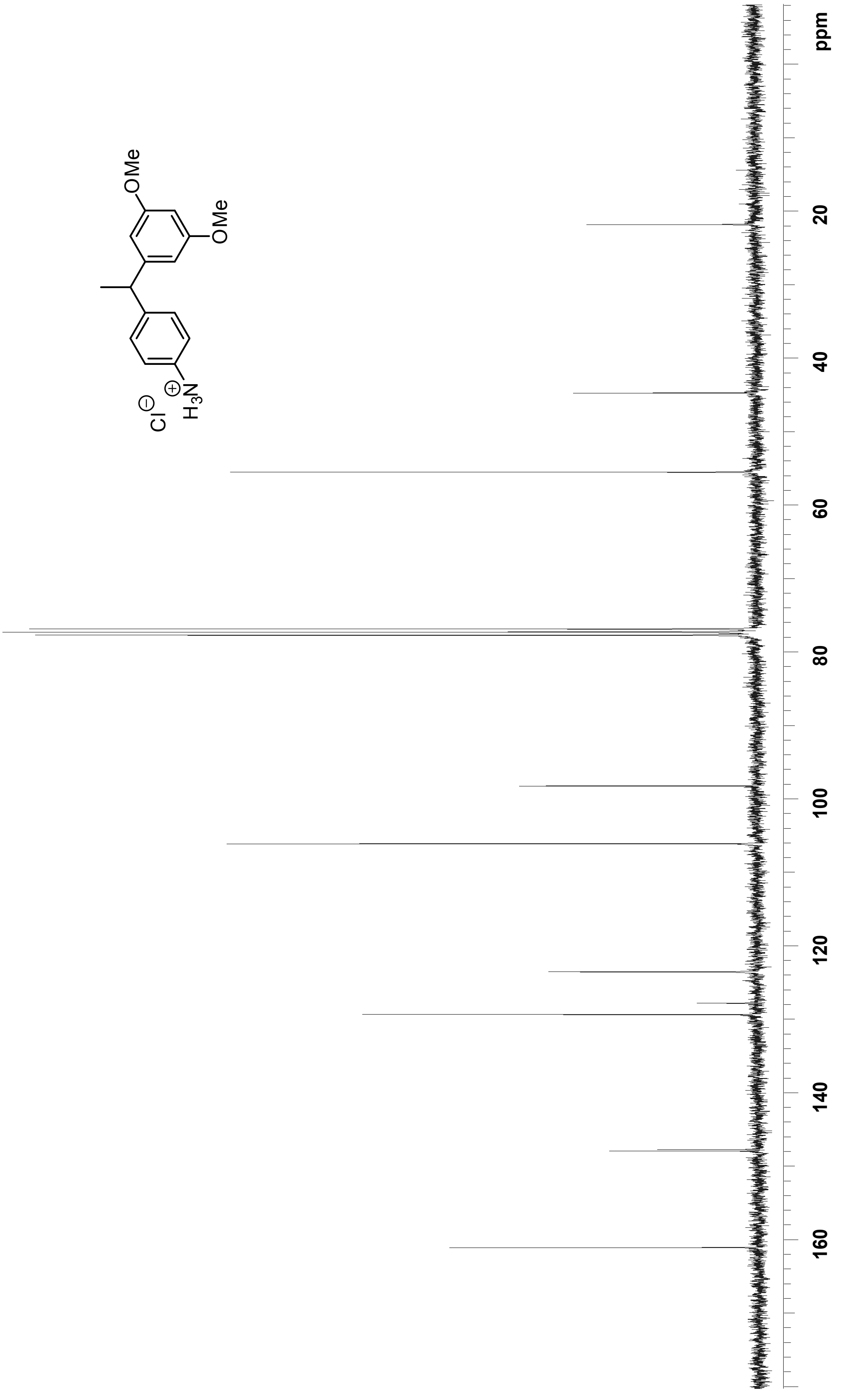
OMe
OMe
Cl
H3N
ppm
20
40
60
80
100
120
140
160

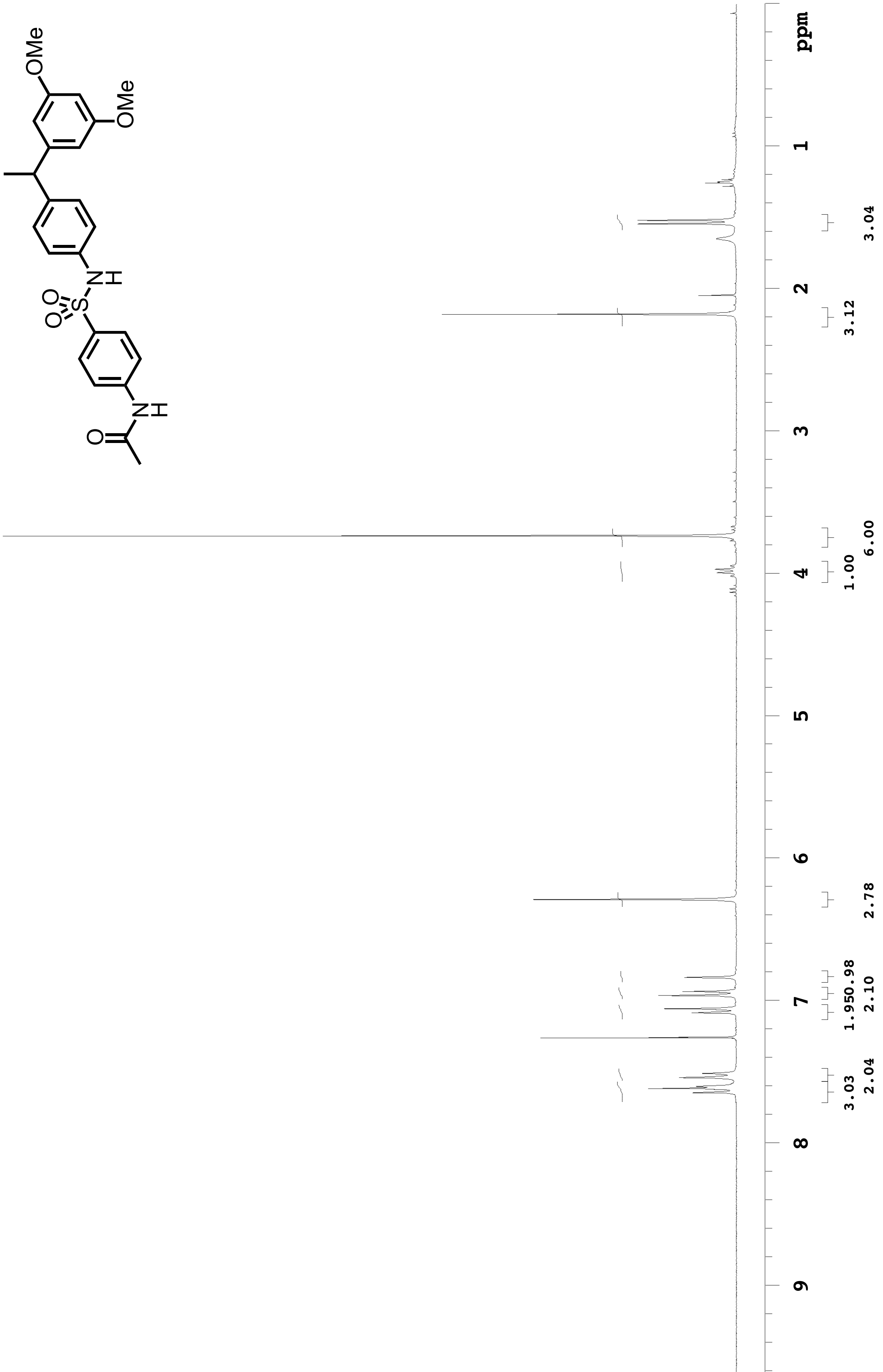

OMe
OMe
ppm
1
2
3
4
5
6
7
8
9
3.04
3.12
1.00
6.00
2.78
1.950.98
2.10
3.03
2.04

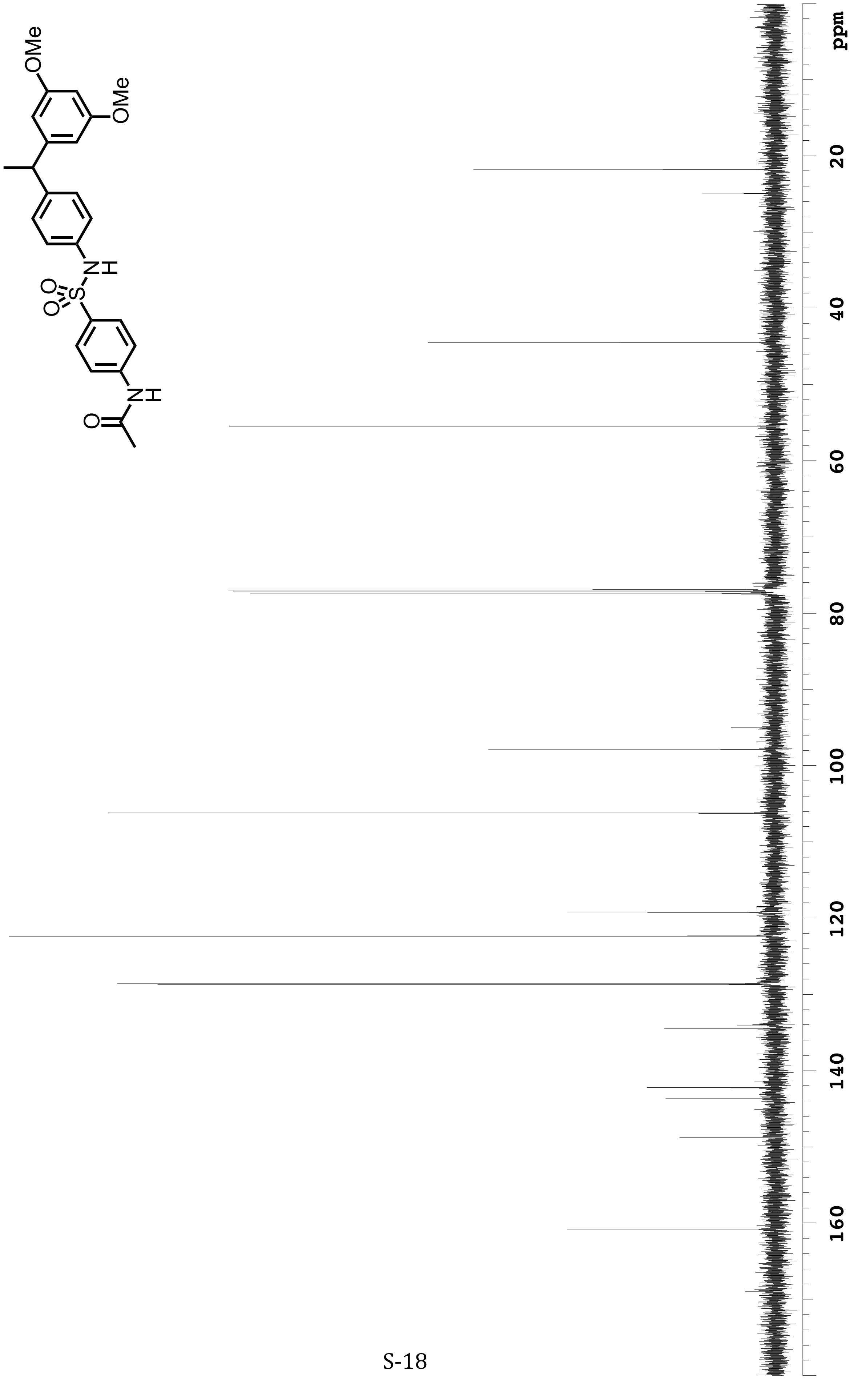
OMe
OMe
ppm
20
40
60
80
100
120
140
160

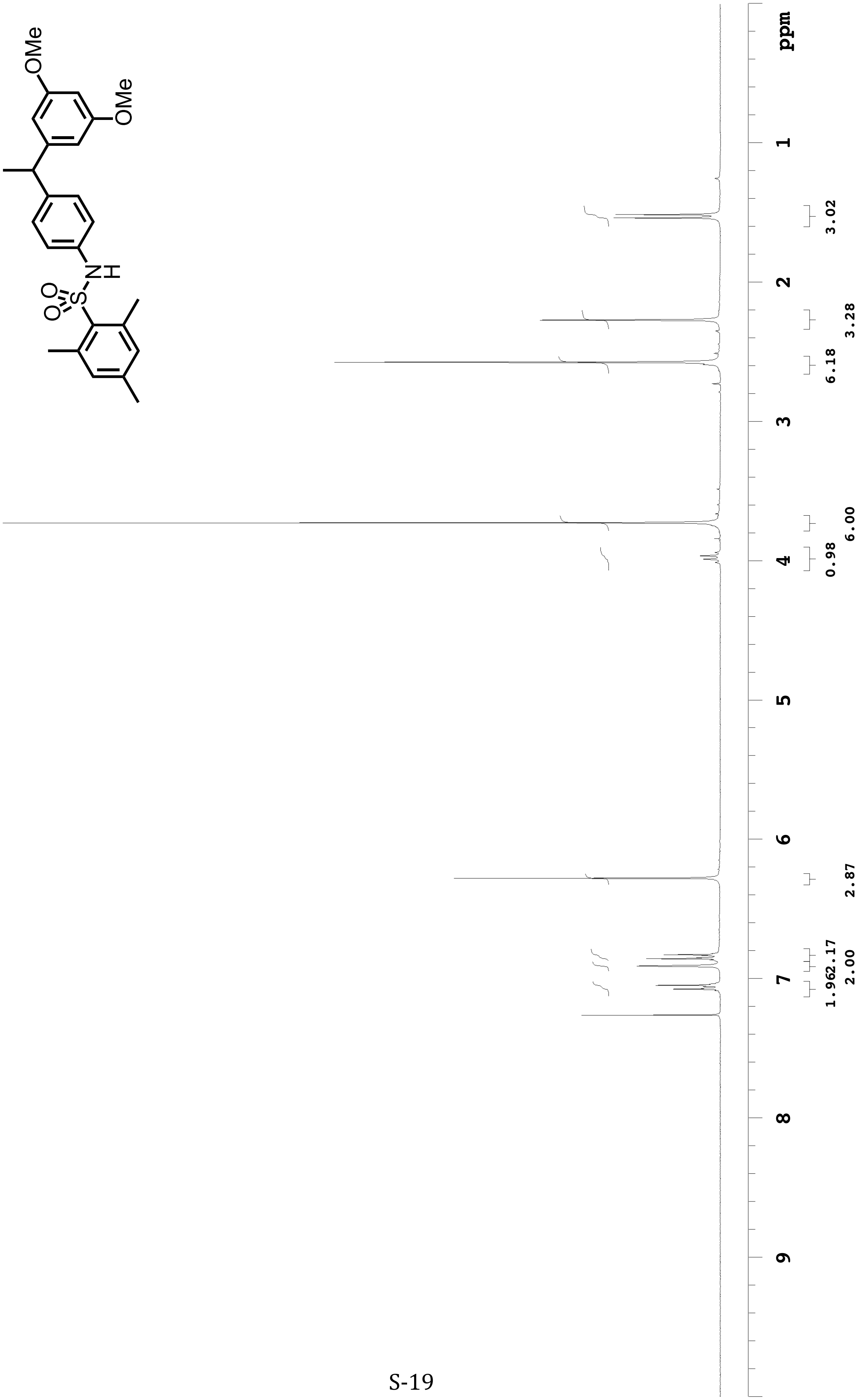
OMe
OMe
N
H
S
O
O
ppm
1
2
3
4
5
6
7
8
9
3.02
3.28
6.18
6.00
0.98
2.87
2.17
1.96
2.00

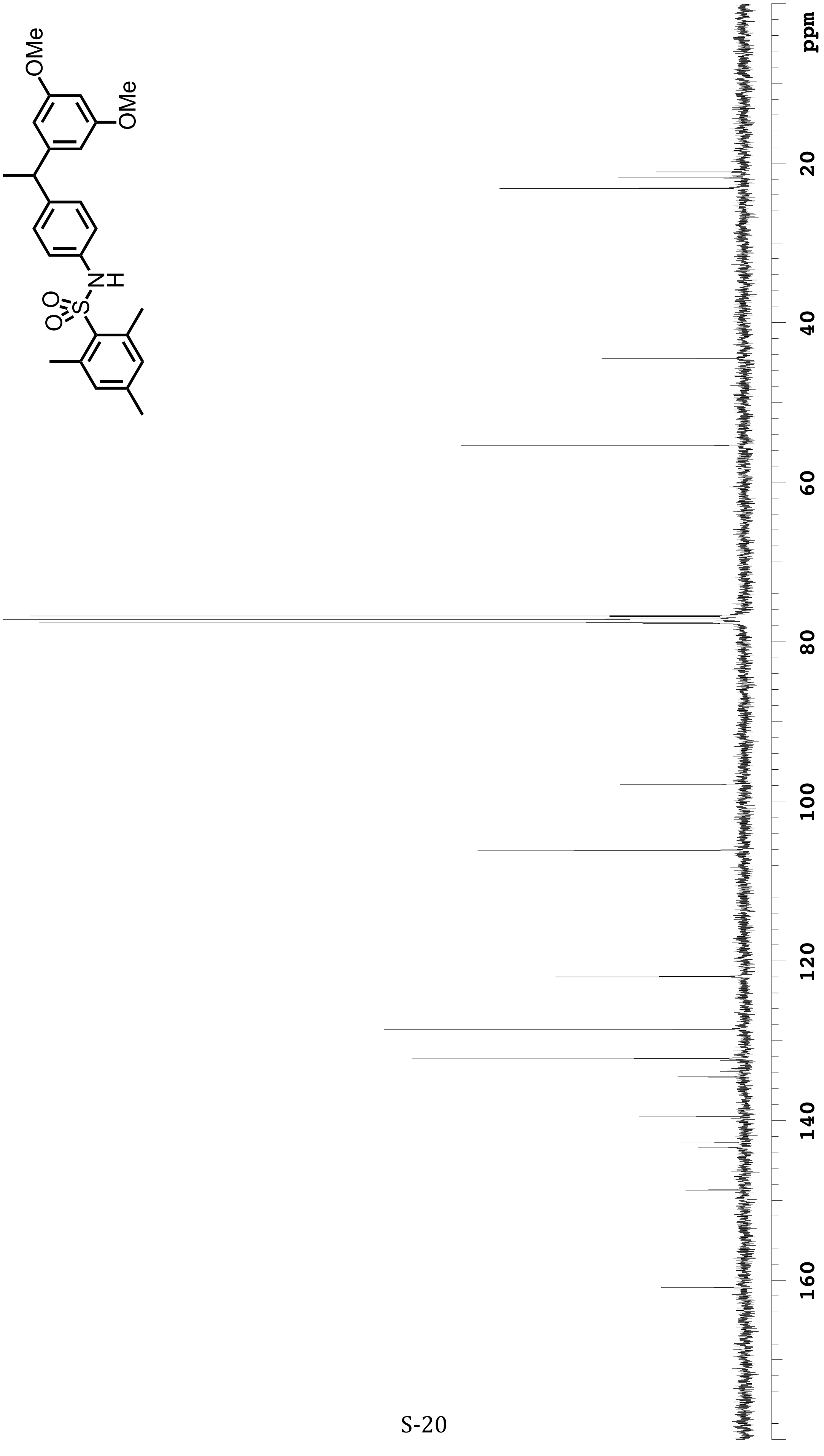
OMe
OMe
O
O
S
N
H
ppm
20
40
60
80
100
120
140
160

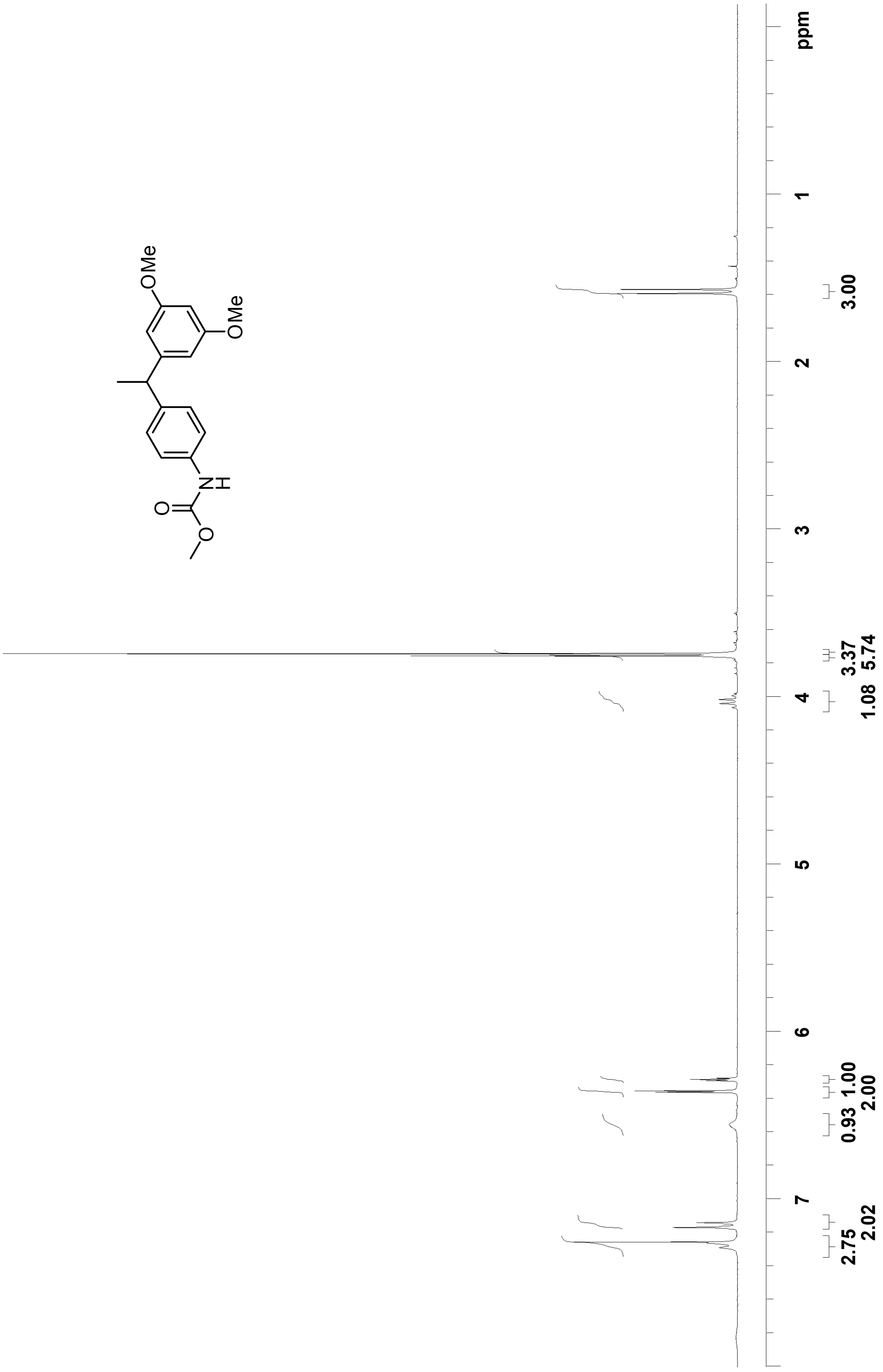

OMe
OMe
N
H
O
O
ppm
1
2
3
4
5
6
7
3.00
3.37
5.74
1.08
1.00
2.00
0.93
2.75
2.02

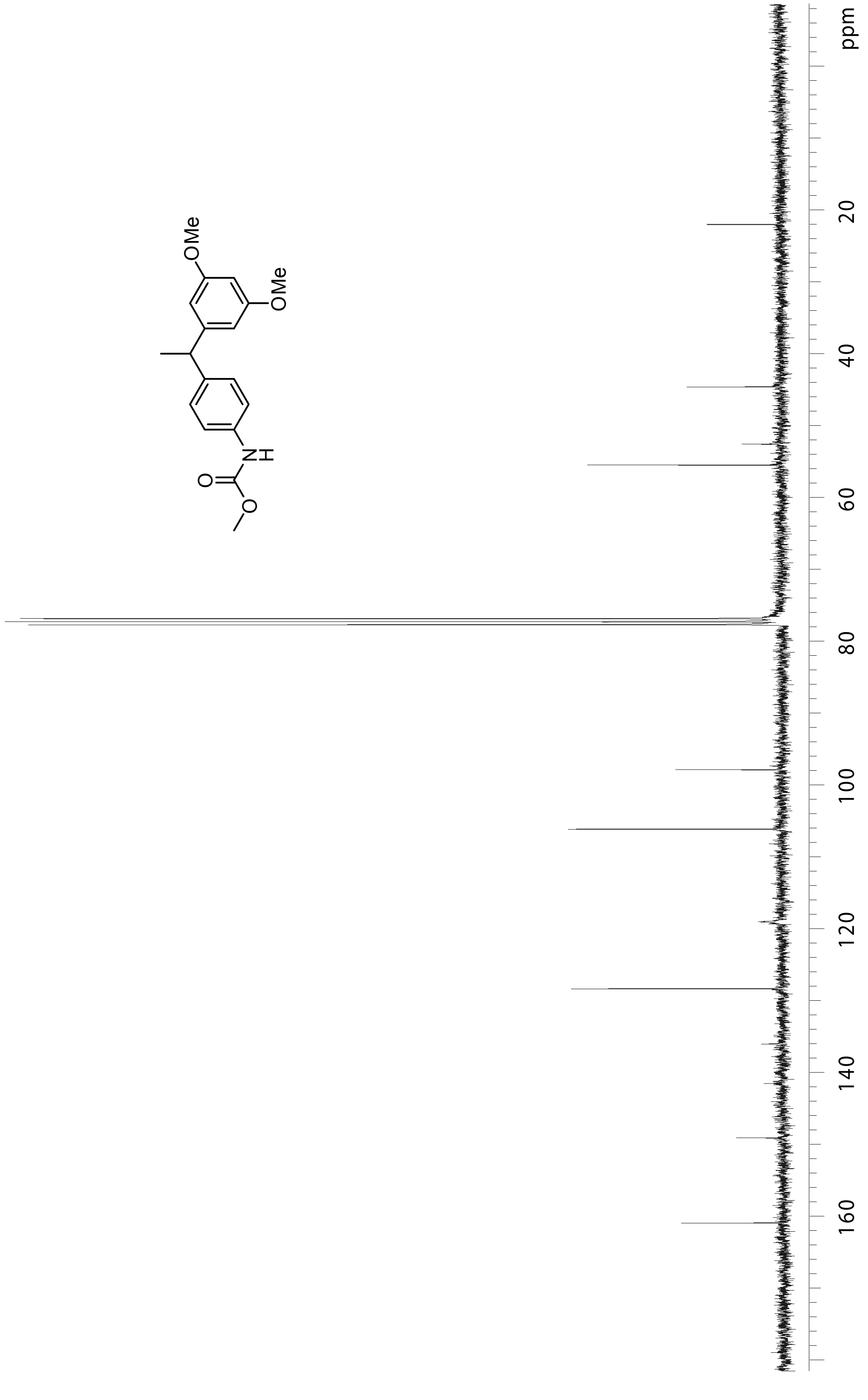
OMe
OMe
N
H
O
O
ppm
20
40
60
80
100
120
140
160

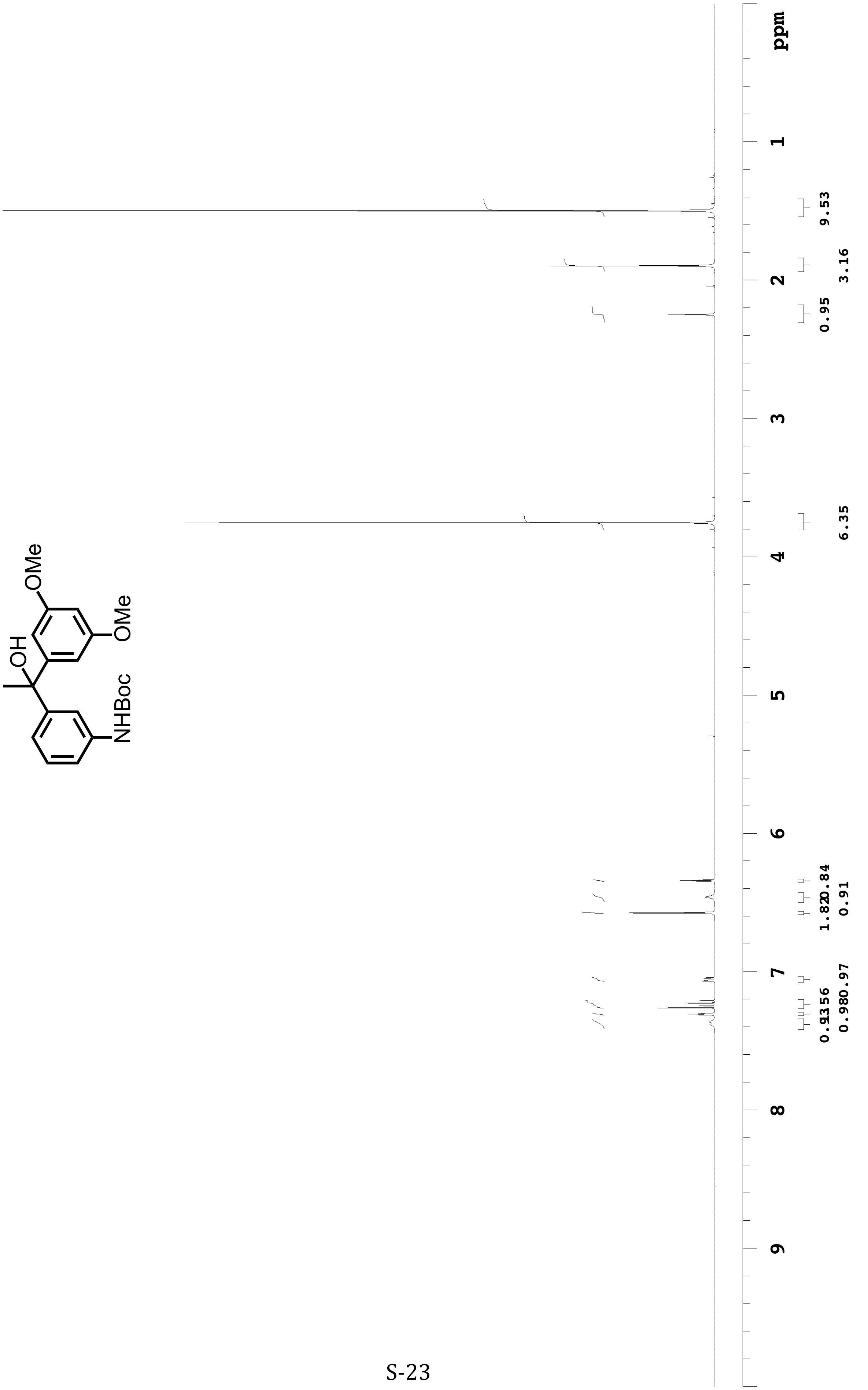
OH
OMe
OMe
NHBoc
ppm
9.53
3.16
0.95
6.35

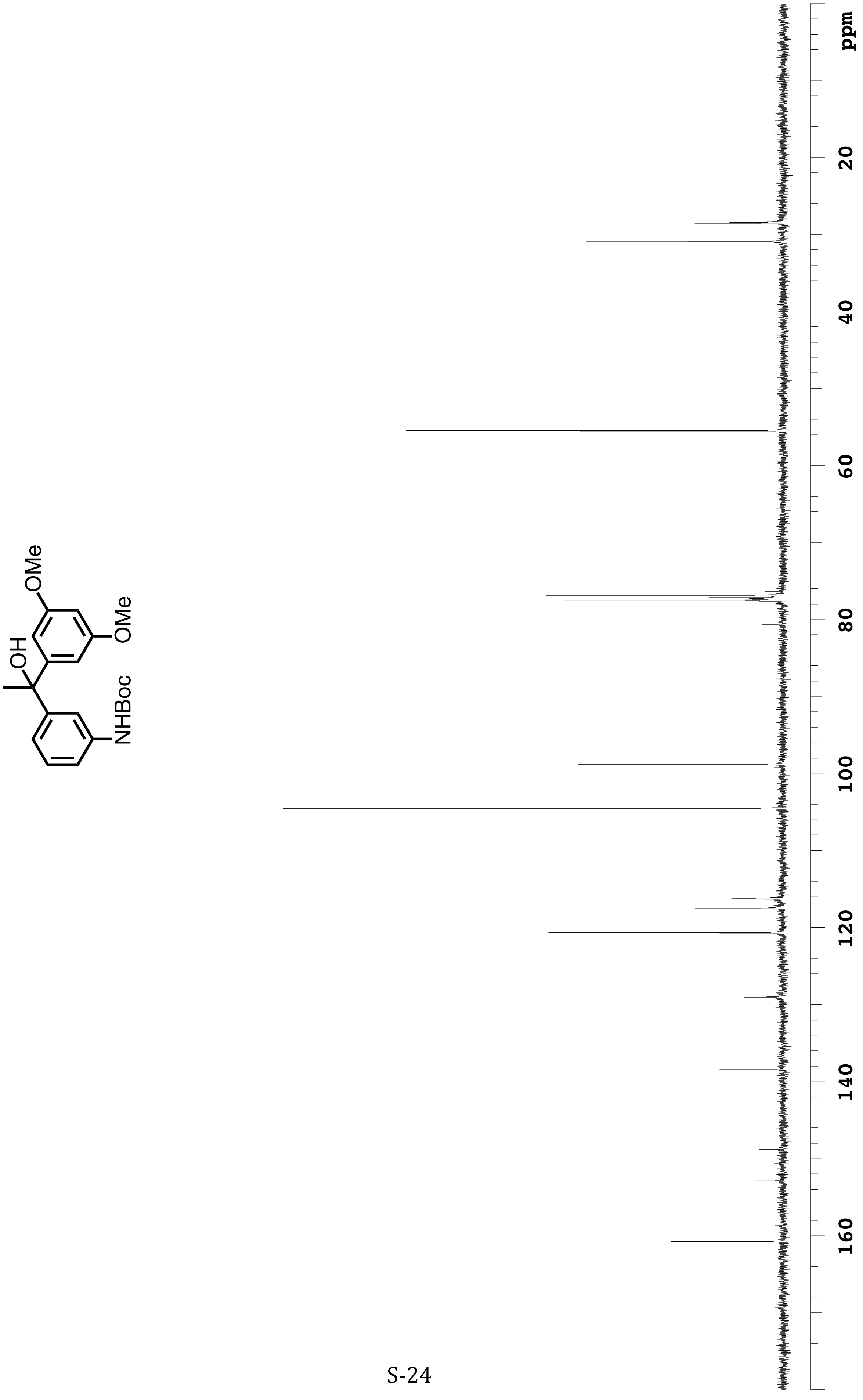
OMe
OMe
OH
NHBoc
ppm
20
40
60
80
100
120
140
160

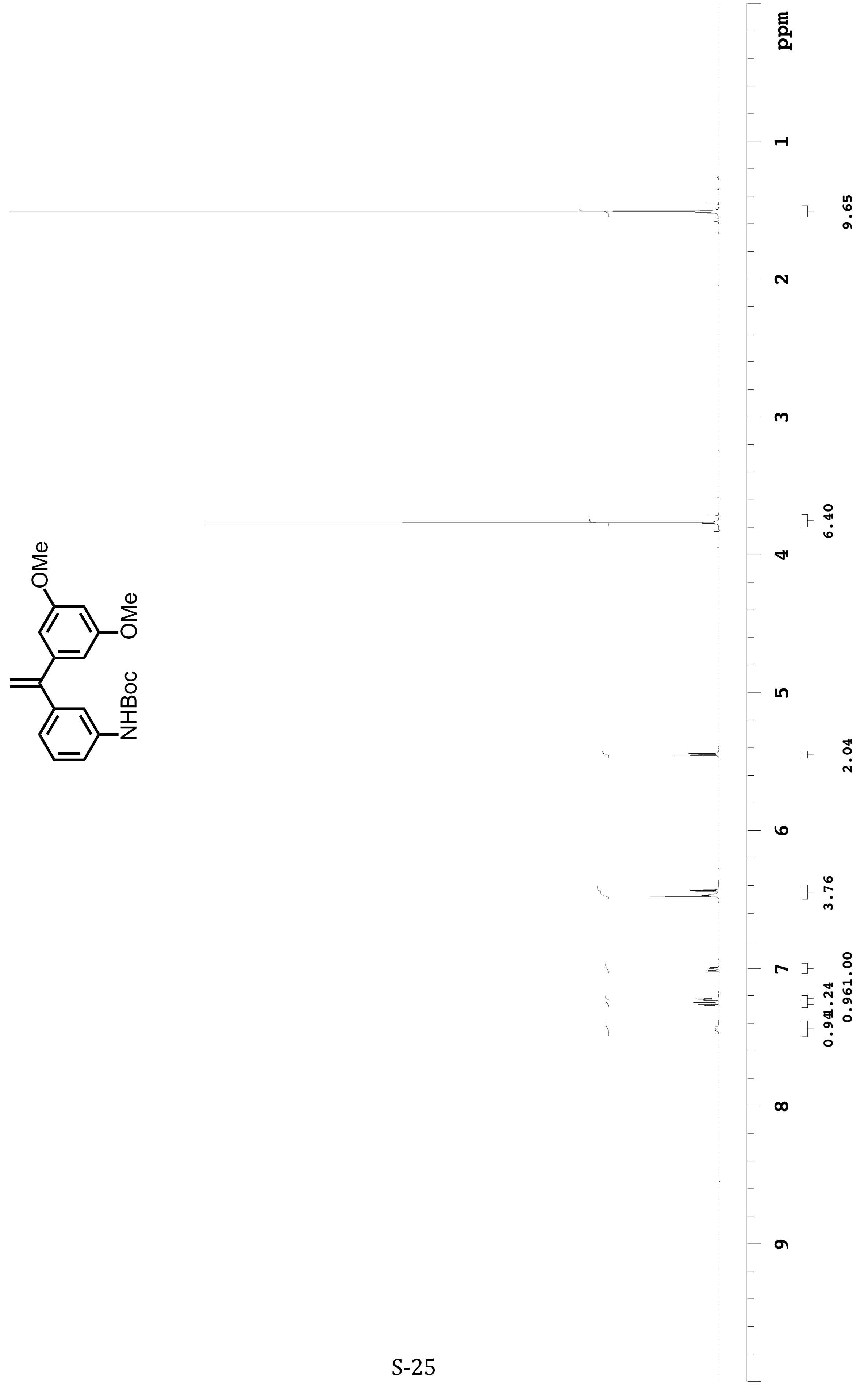
OMe
OMe
NHBoc
ppm
1
2
3
4
5
6
7
8
9
9.65
6.40
2.04
3.76
1.00
0.96
1.24
0.94

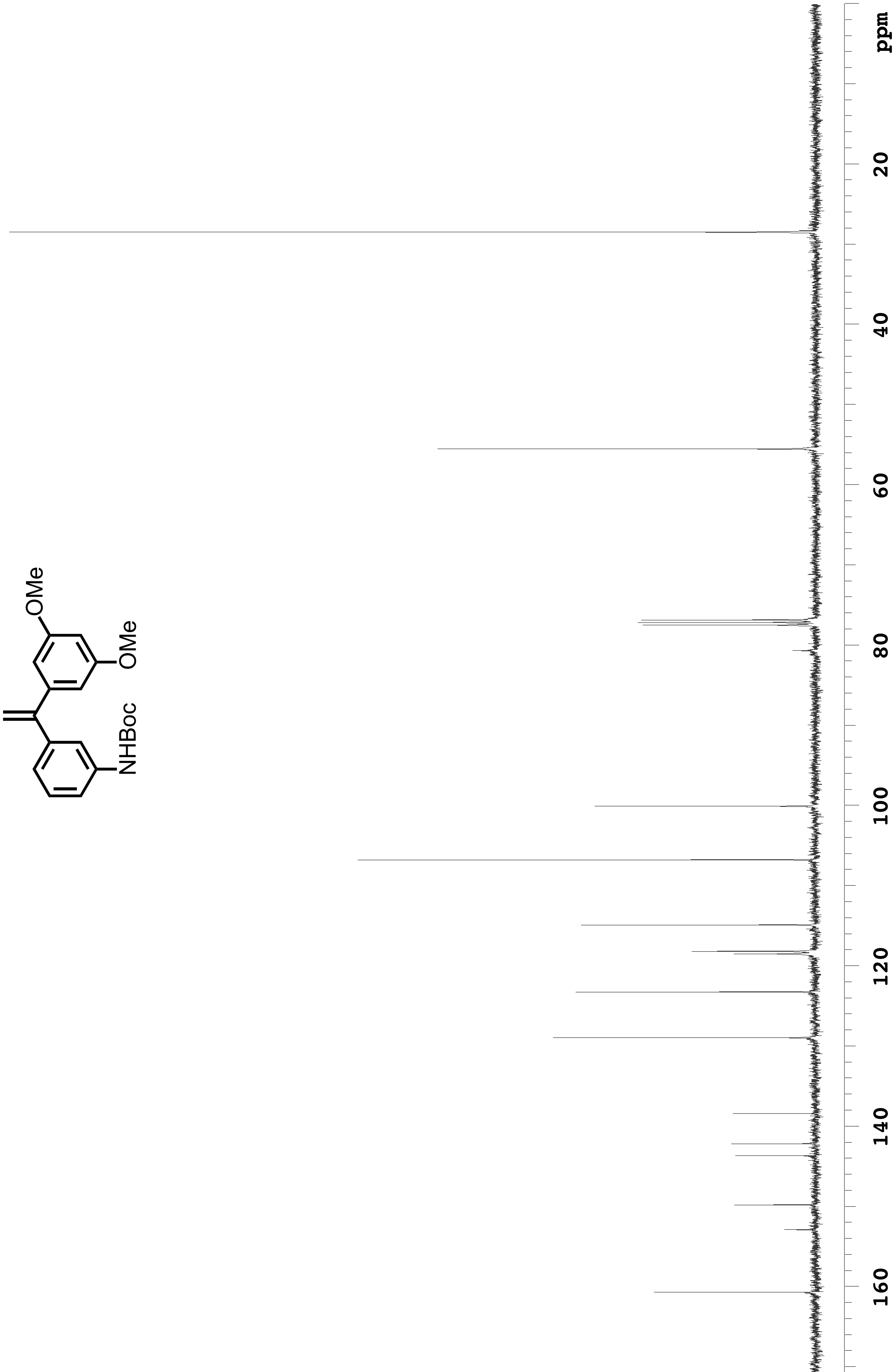
OMe
OMe
NHBoc
ppm
20
40
60
80
100
120
140
160

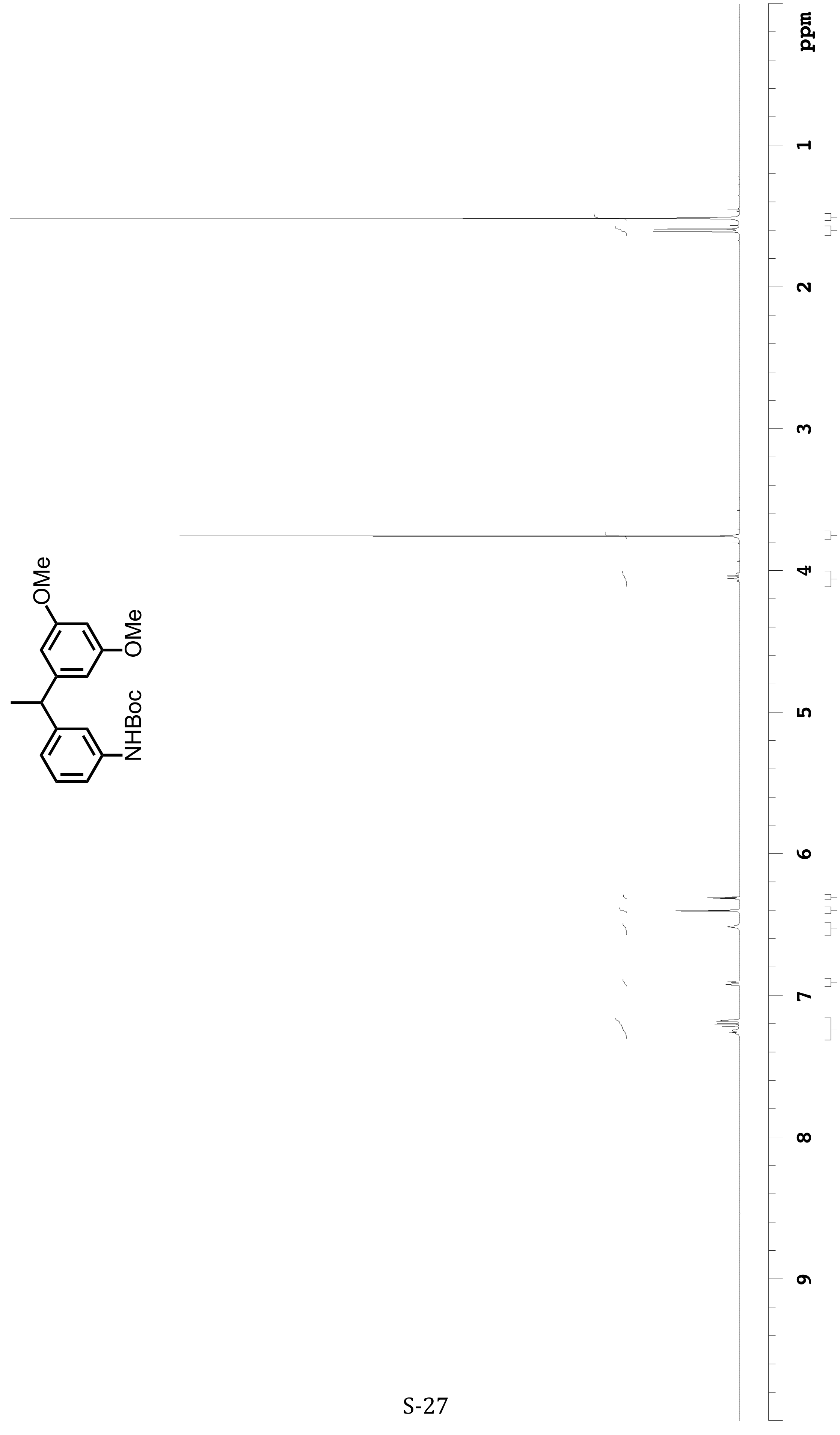
OMe
OMe
NHBoc
ppm
1
2
3
4
5
6
7
8
9
9.43
3.22
6.26
1.09
1.020.87
1.93
1.00
3.18

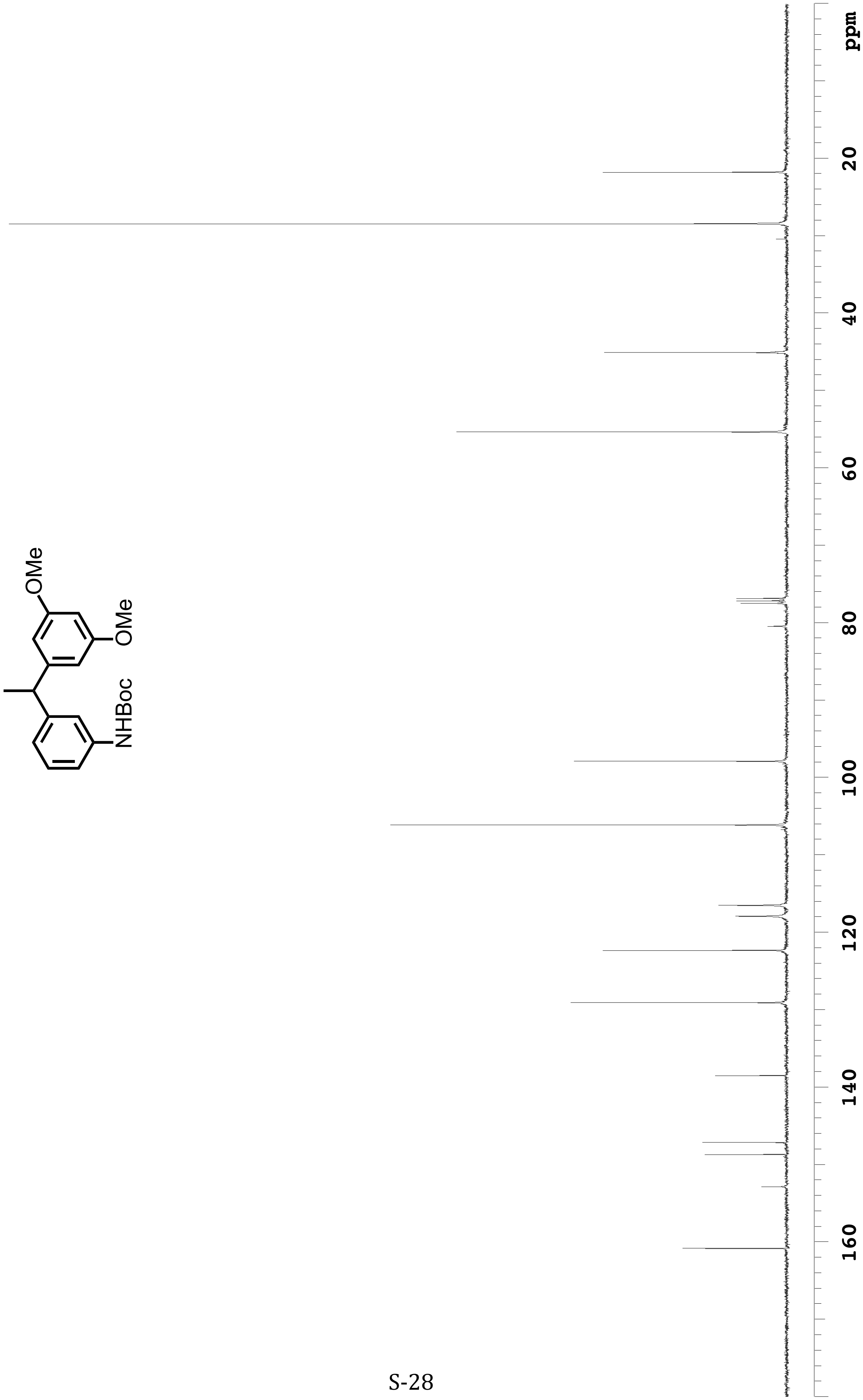
OMe
OMe
NHBoc
ppm
20
40
60
80
100
120
140
160

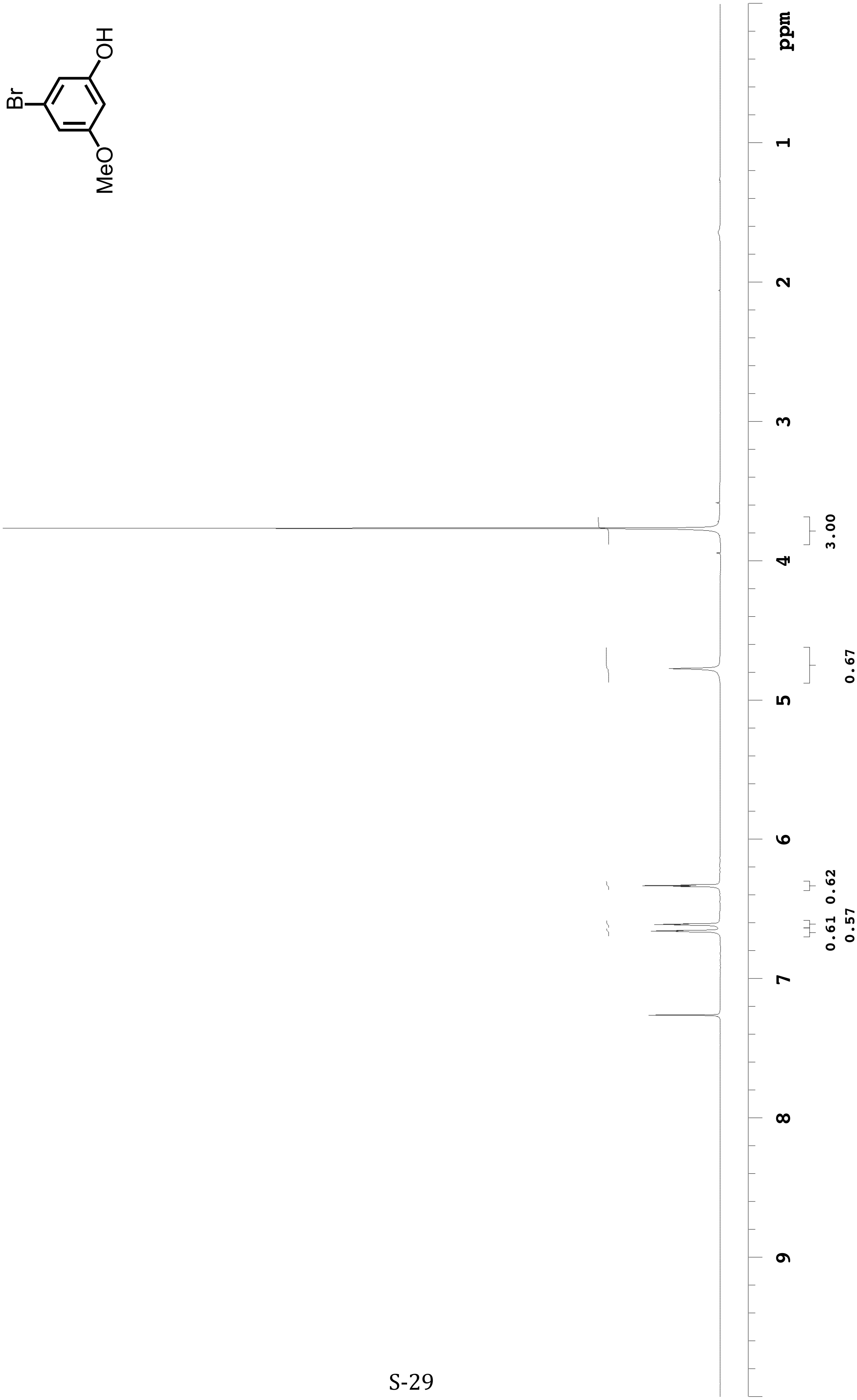

Br
OH
MeO
ppm
1
2
3
4
5
6
7
8
9
3.00
0.67
0.61 0.62
0.57

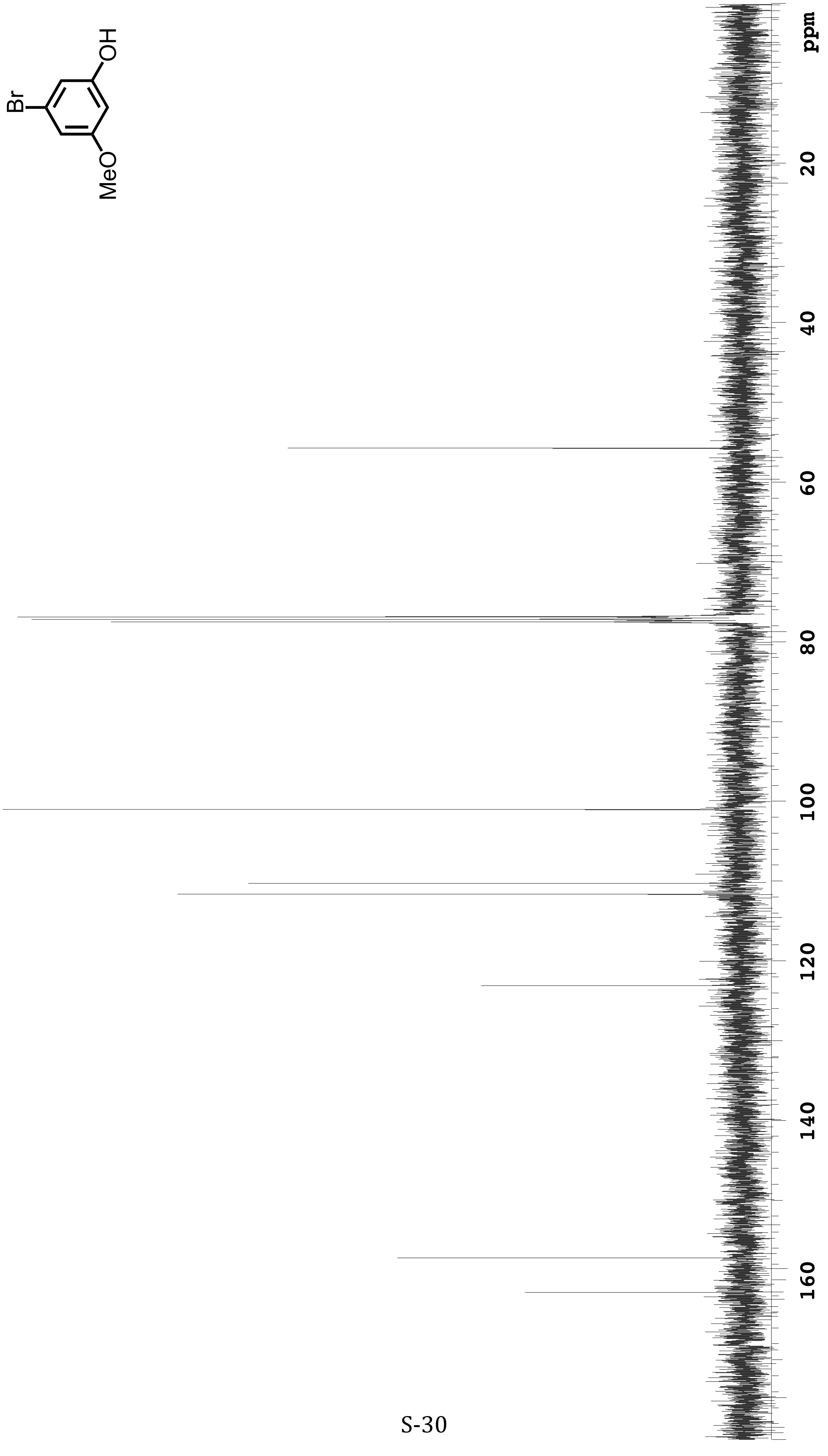
Br
OH
MeO
ppm
20
40
60
80
100
120
140
160

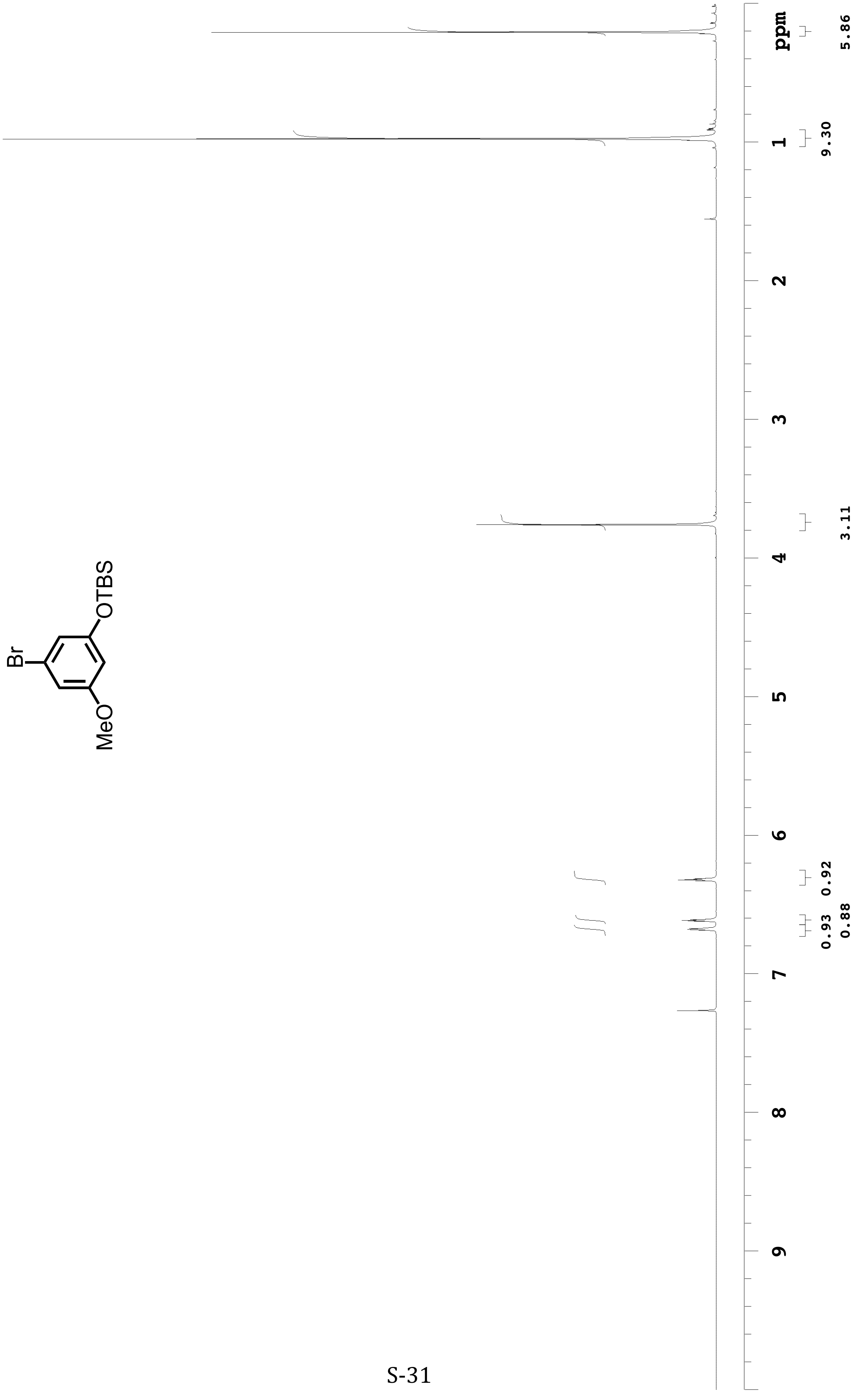

Br
OTBS
MeO
ppm
1
2
3
4
5
6
7
8
9
5.86
9.30
3.11
0.92
0.93
0.88

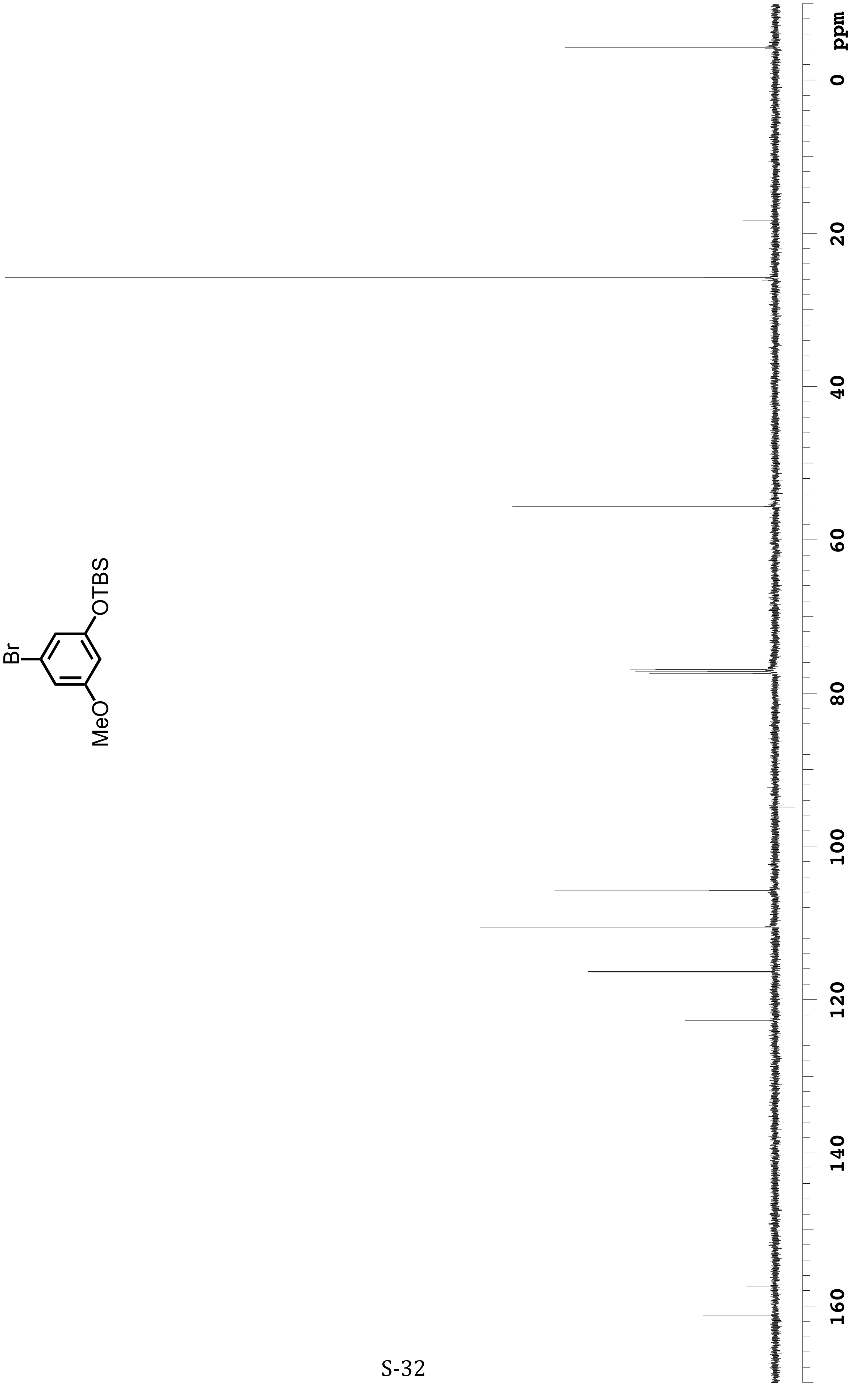
OTBS
Br
MeO
ppm
0
20
40
60
80
100
120
140
160

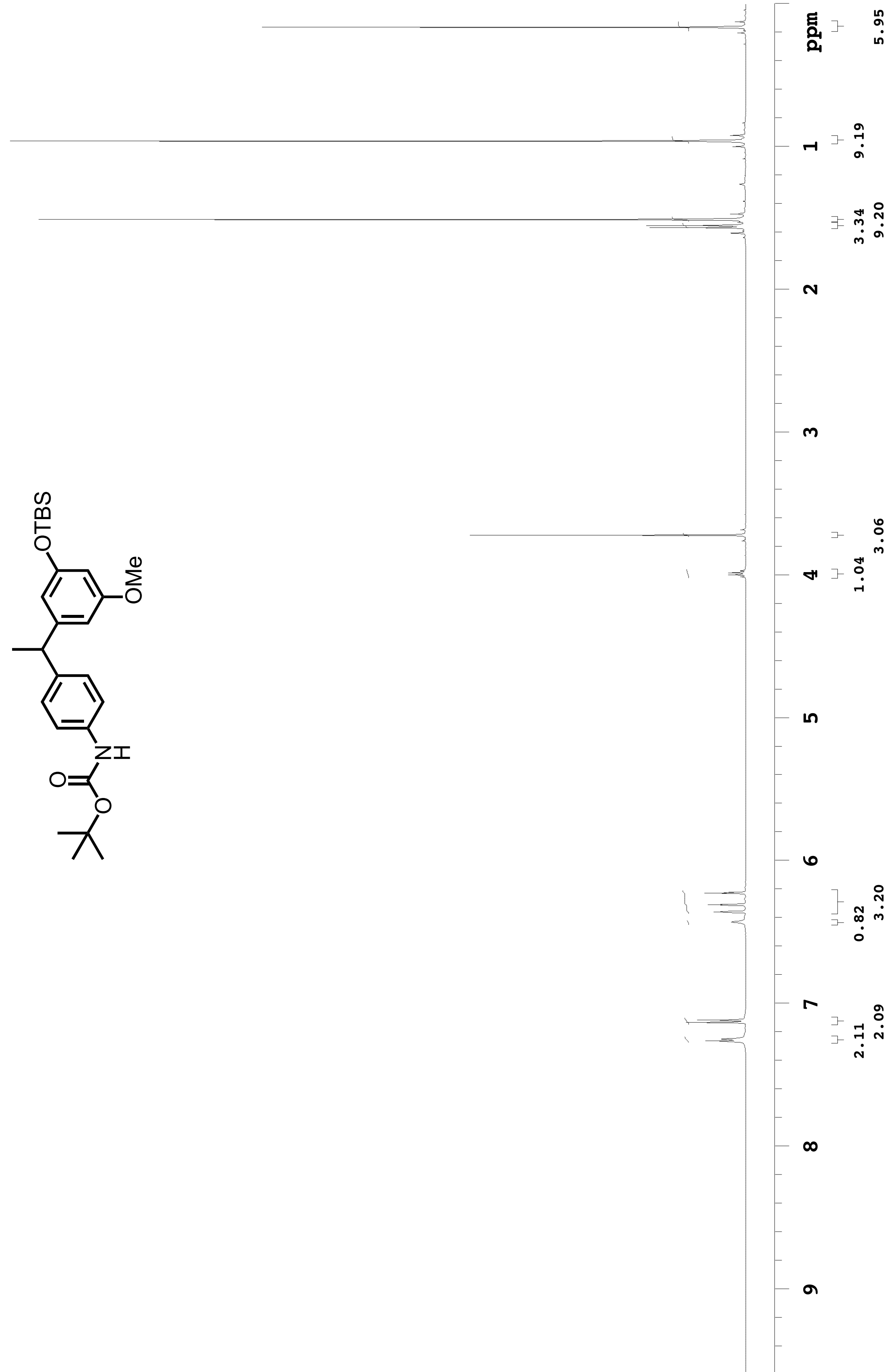
OTBS
OMe
ppm
1
2
3
4
5
6
7
8
9
5.95
9.19
3.34
9.20
1.04
3.06
0.82
3.20
2.11
2.09

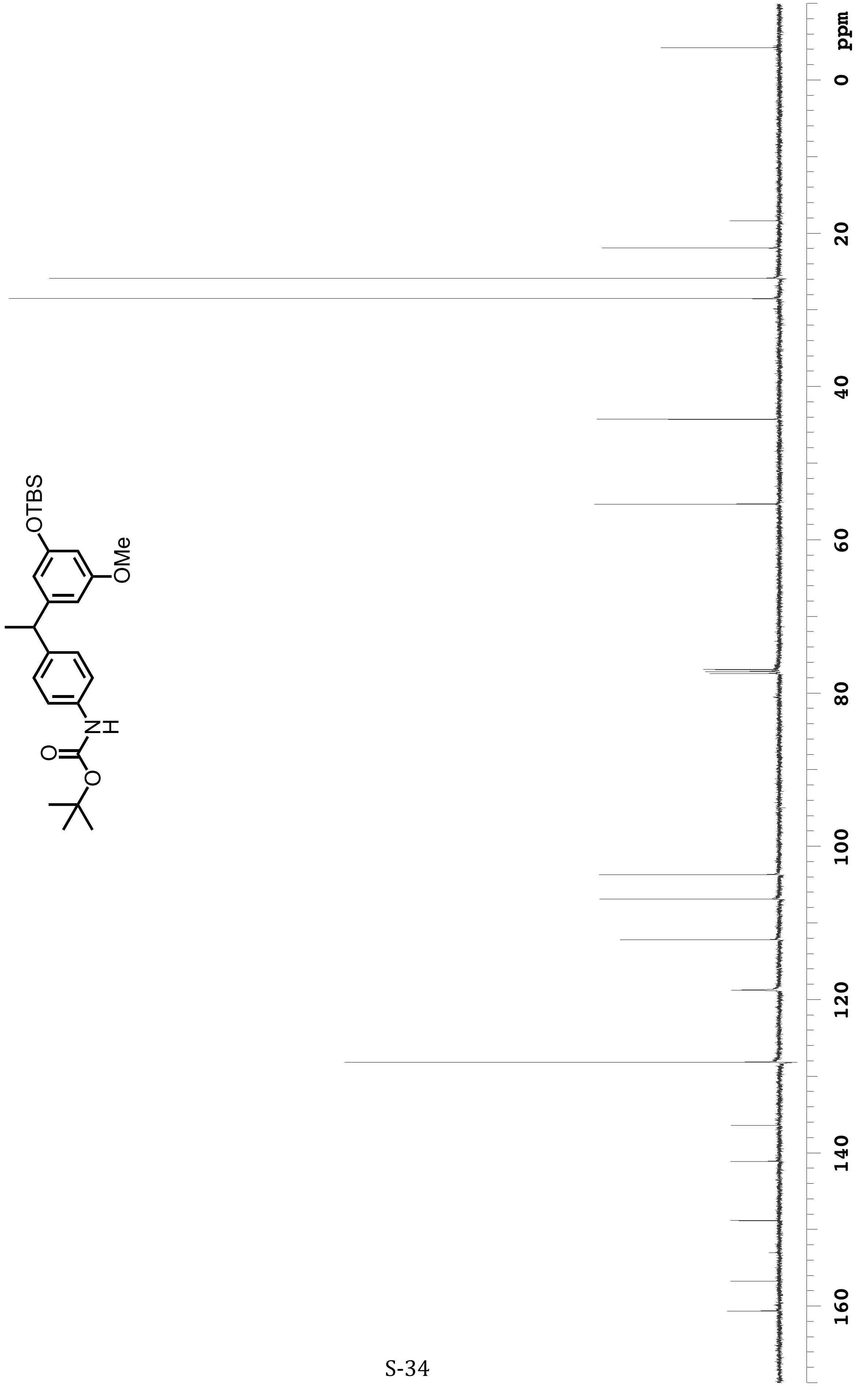
OTBS
OMe
O
O
N
H
ppm
0
20
40
60
80
100
120
140
160

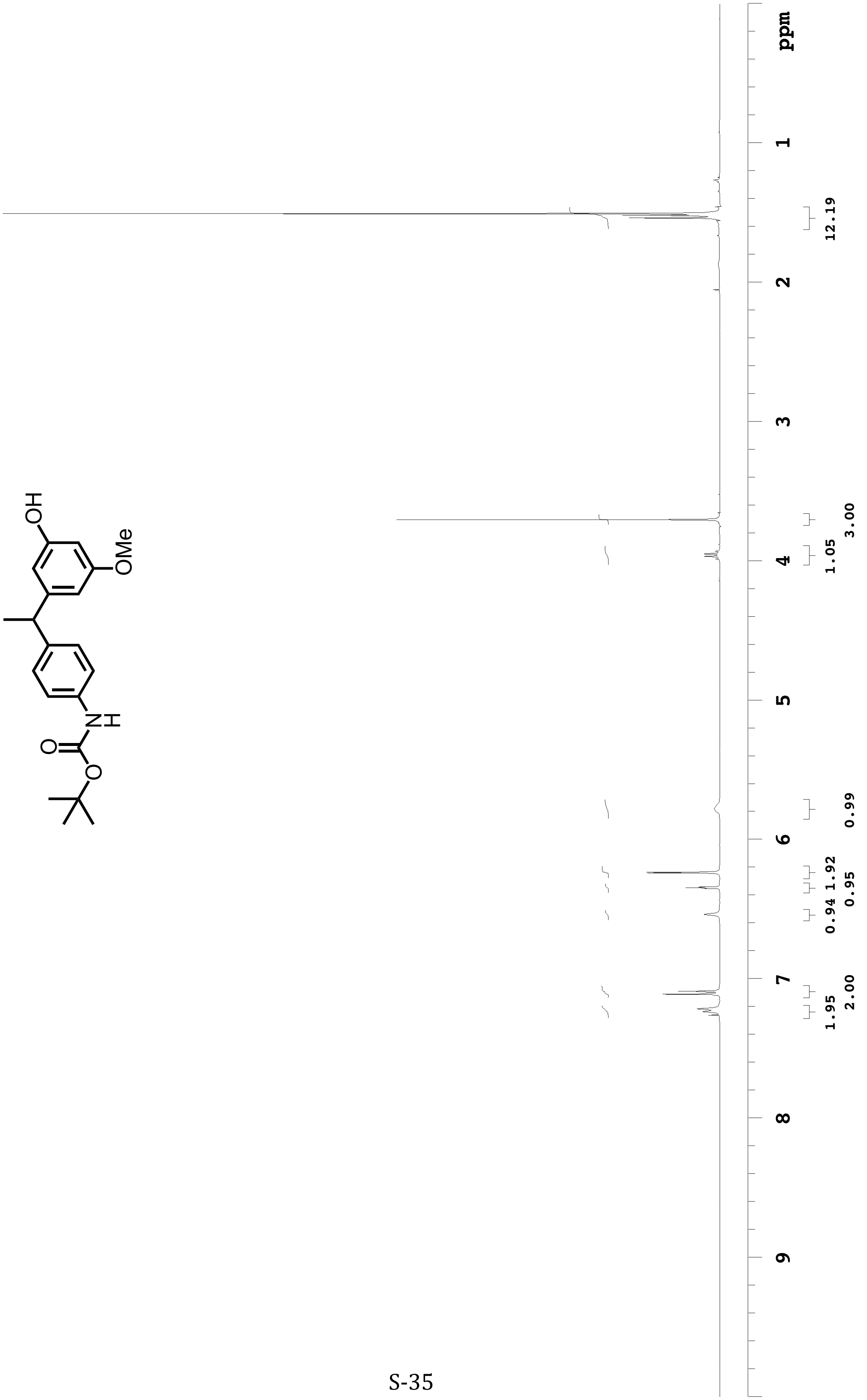
ppm
1
2
3
4
5
6
7
8
9
12.19
1.05
3.00
0.99
0.94
1.92
0.95
1.95
2.00
OH
OMe
N
H
O
O

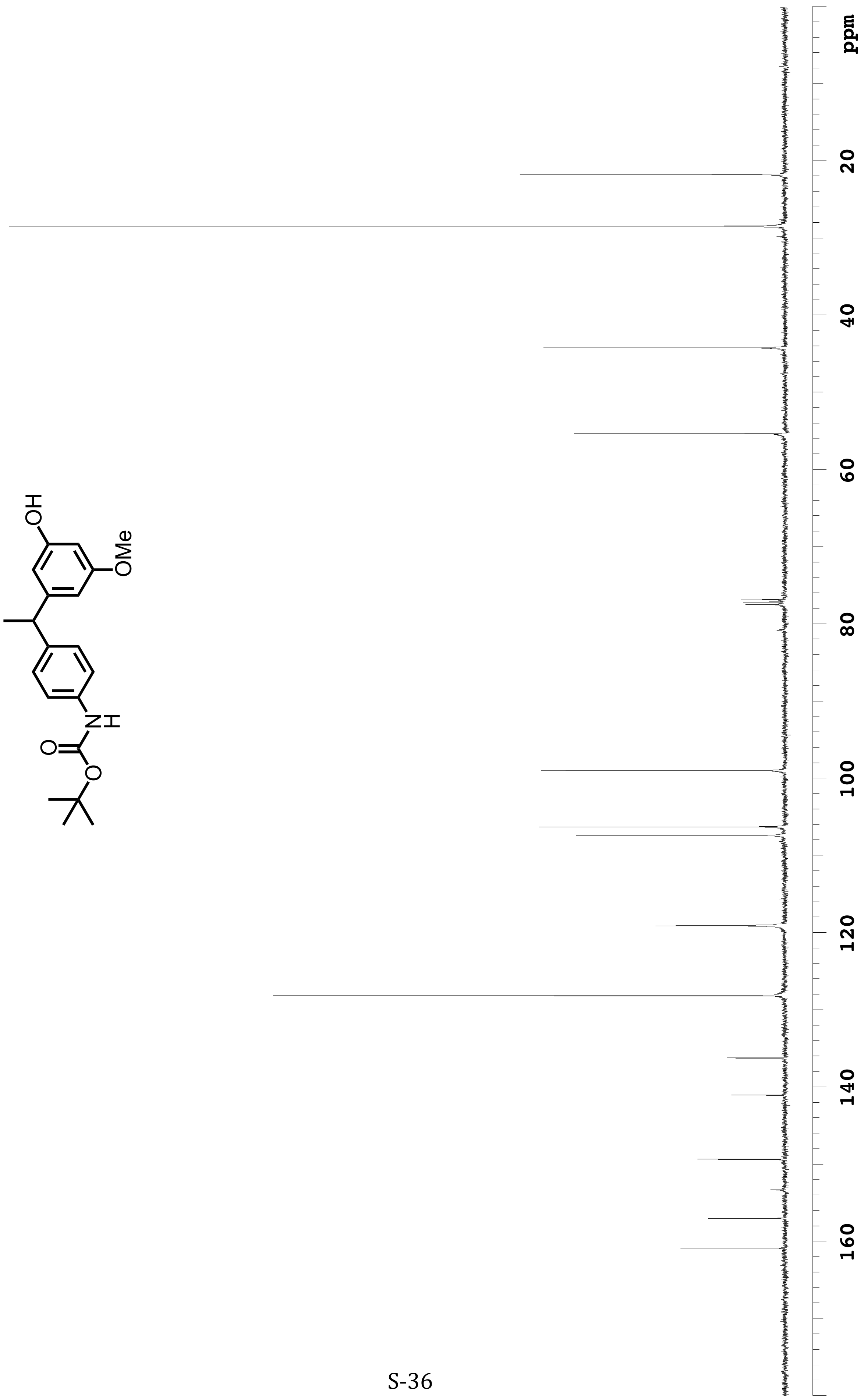
ppm
20
40
60
80
100
120
140
160
HO
OMe
N
H
O
O

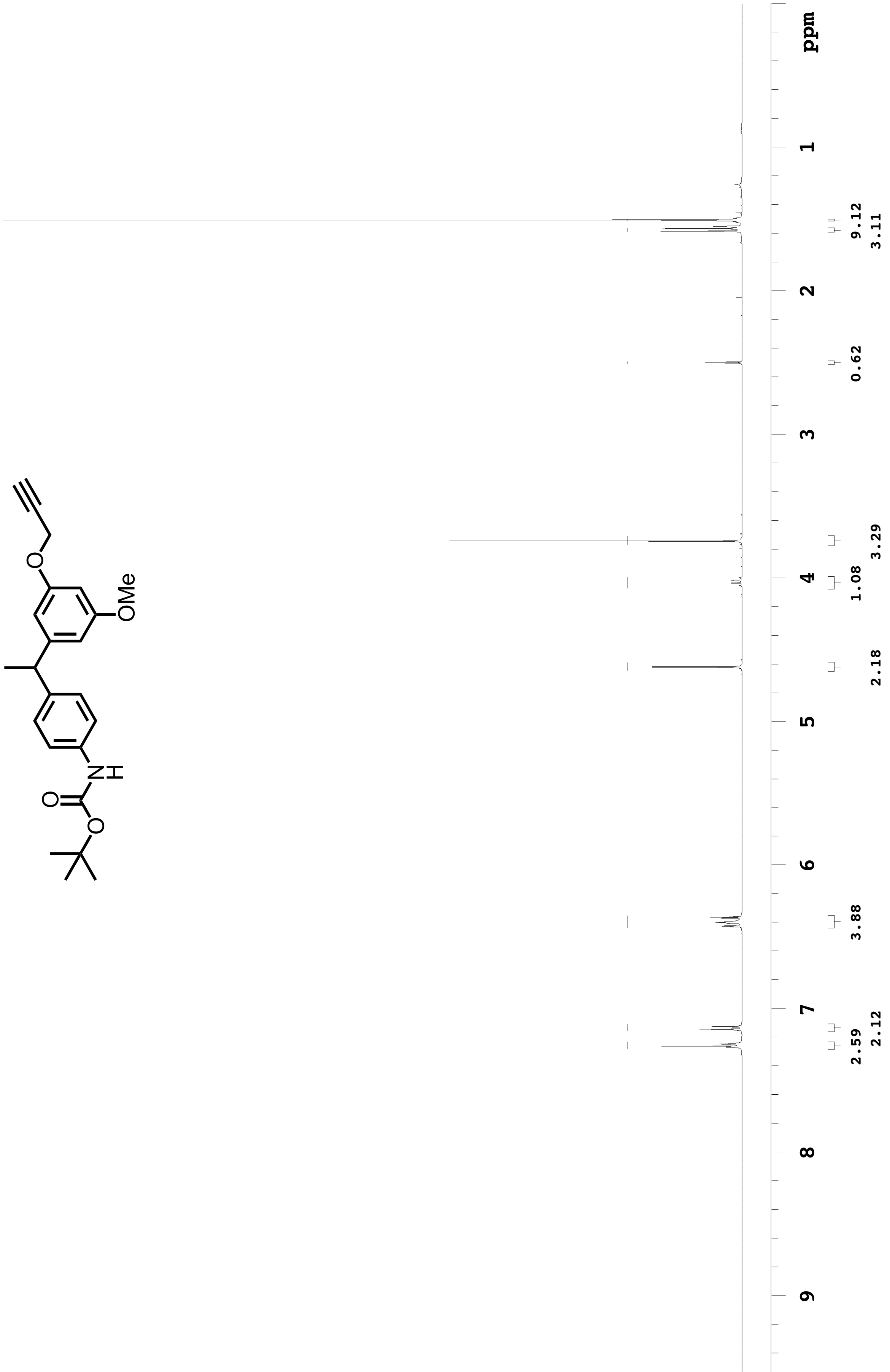
OMe
ppm
1
2
3
4
5
6
7
8
9
9.12
3.11
0.62
3.29
1.08
2.18
3.88
2.59
2.12

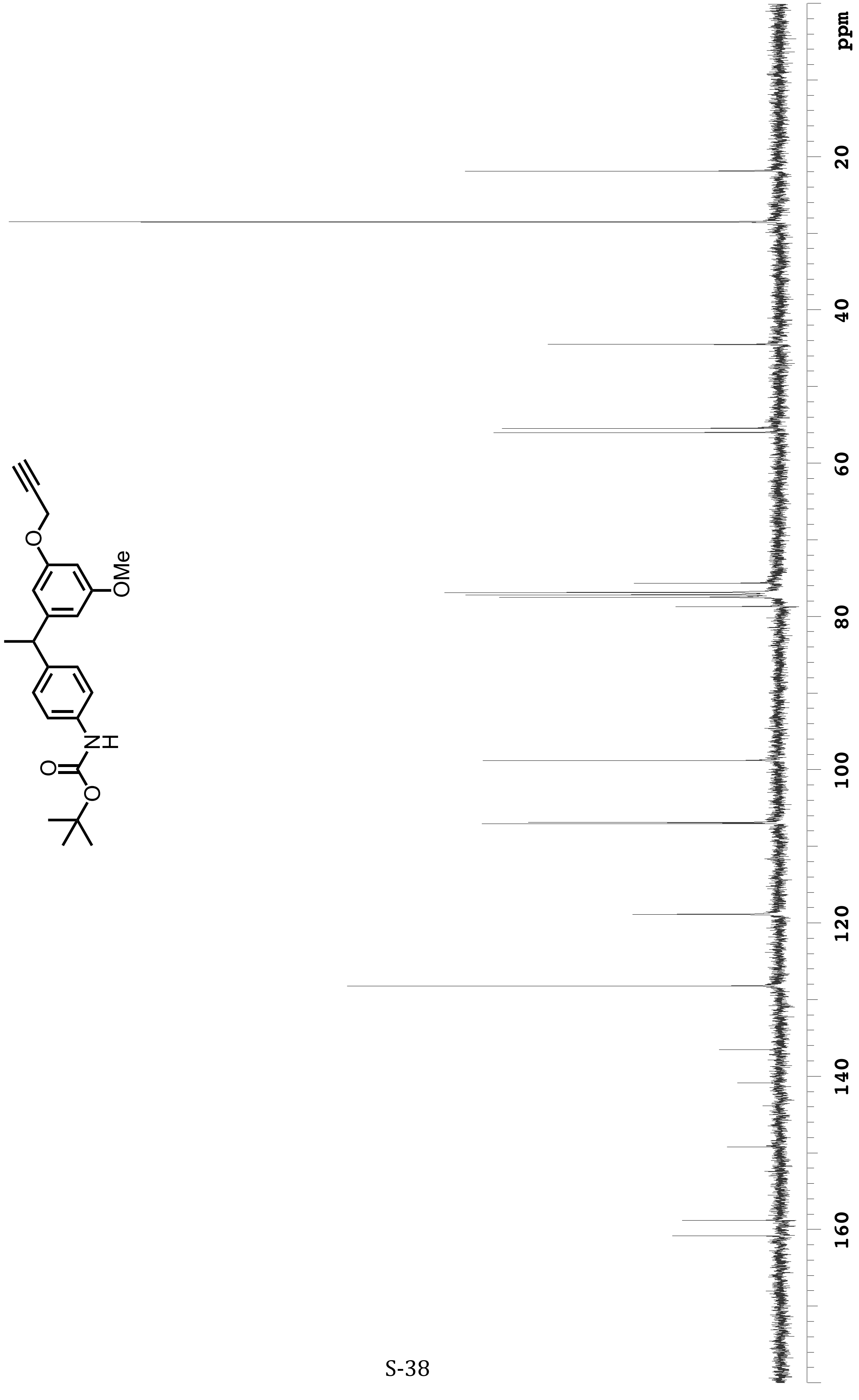
OMe
ppm
20
40
60
80
100
120
140
160

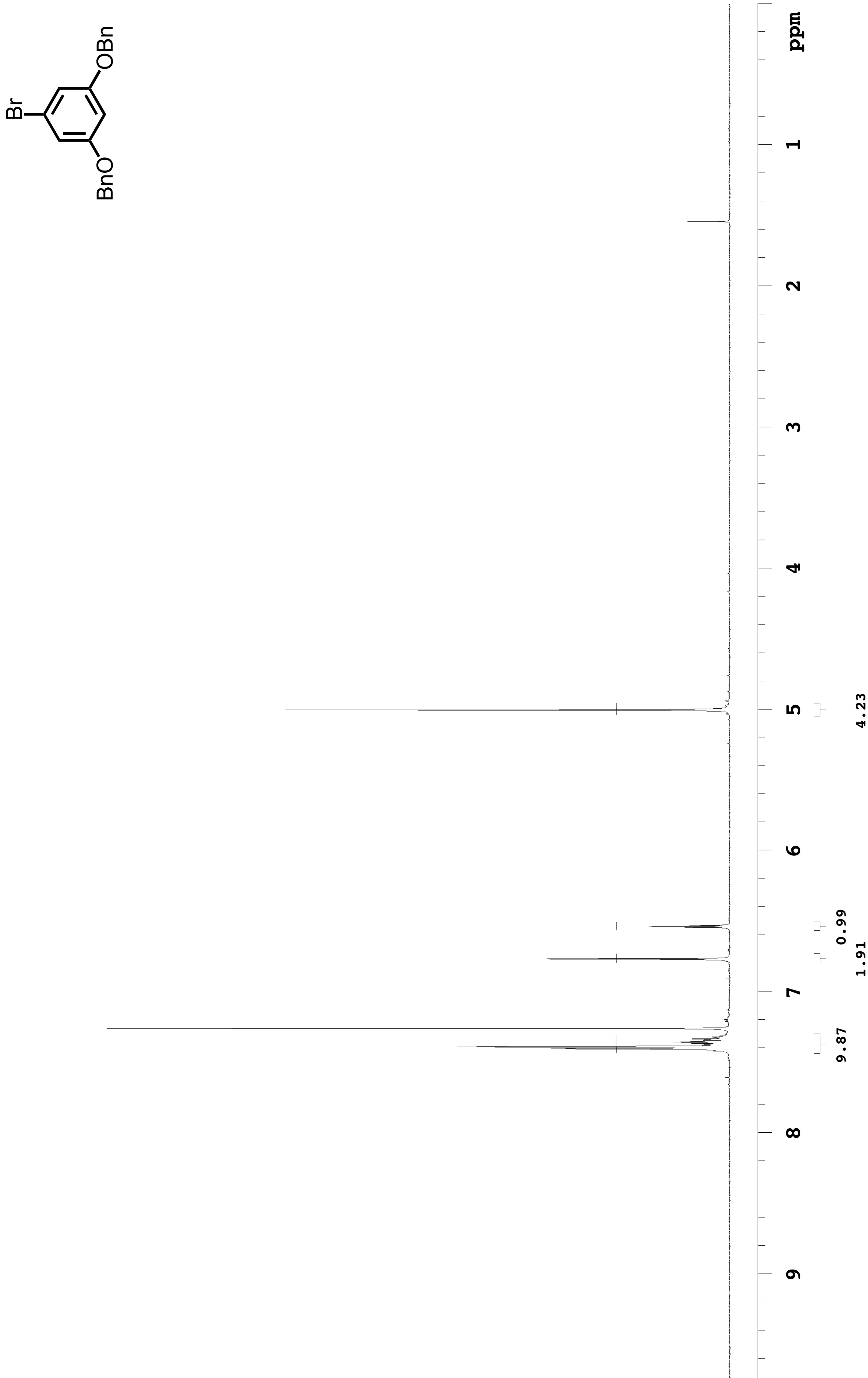

Br
BnO
OBn
ppm
1
2
3
4
5
6
7
8
9
4.23
0.99
1.91
9.87

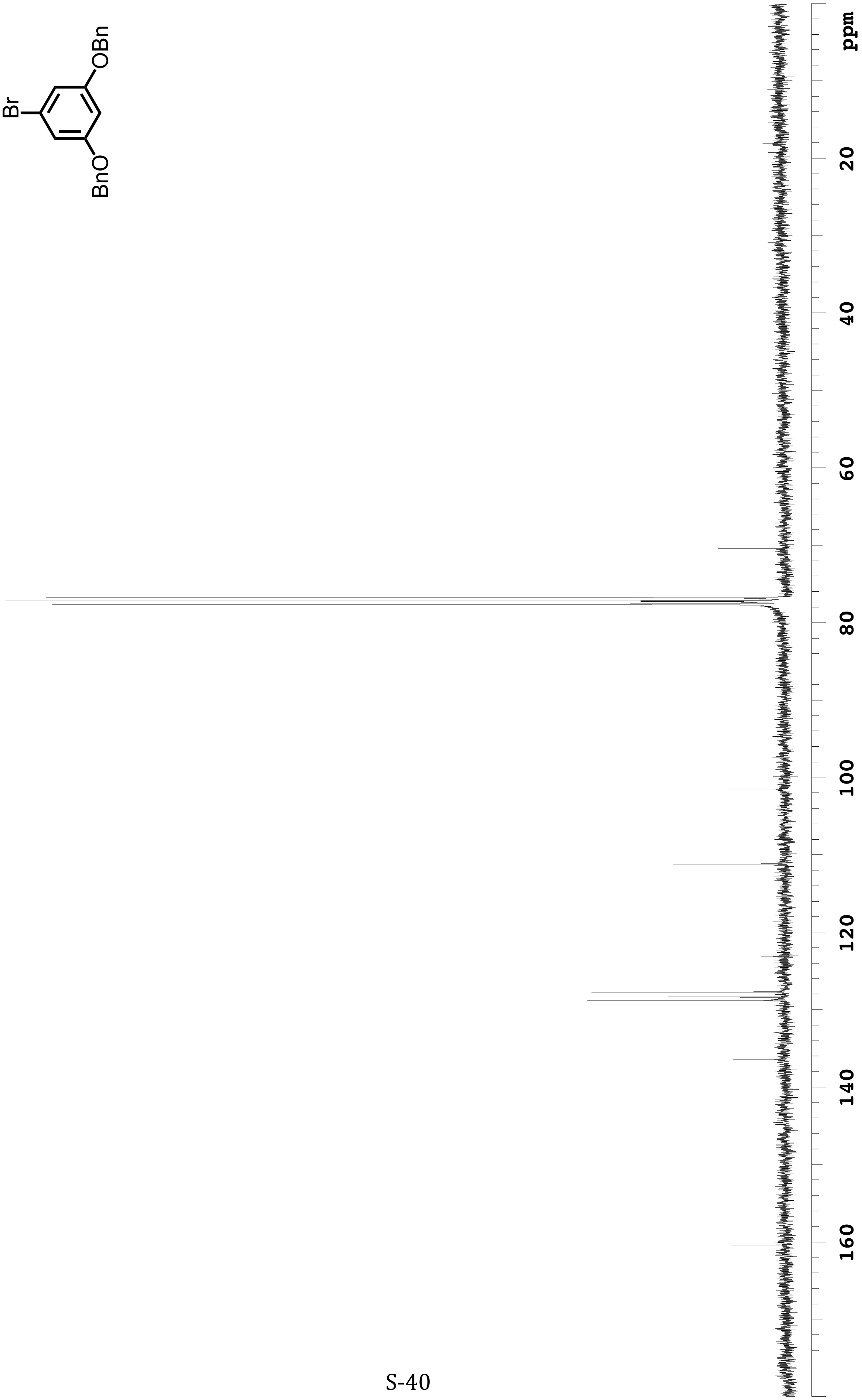

Br
BnO
OBn
ppm
20
40
60
80
100
120
140
160

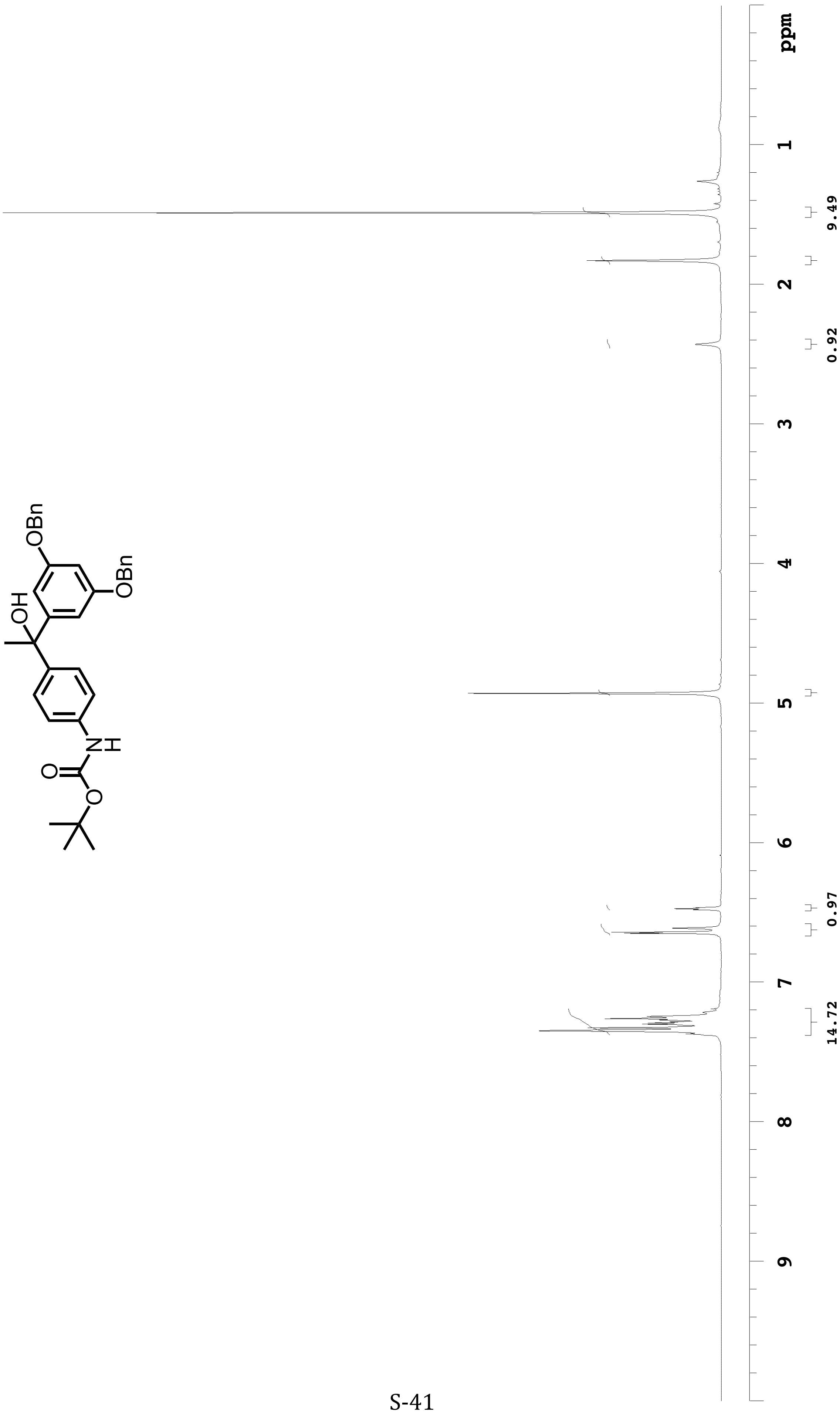

OBn
OBn
OH
N
H
O
O
ppm
1
2
3
4
5
6
7
8
9
9.49
2.91
0.92
3.94
0.97
3.05
14.72

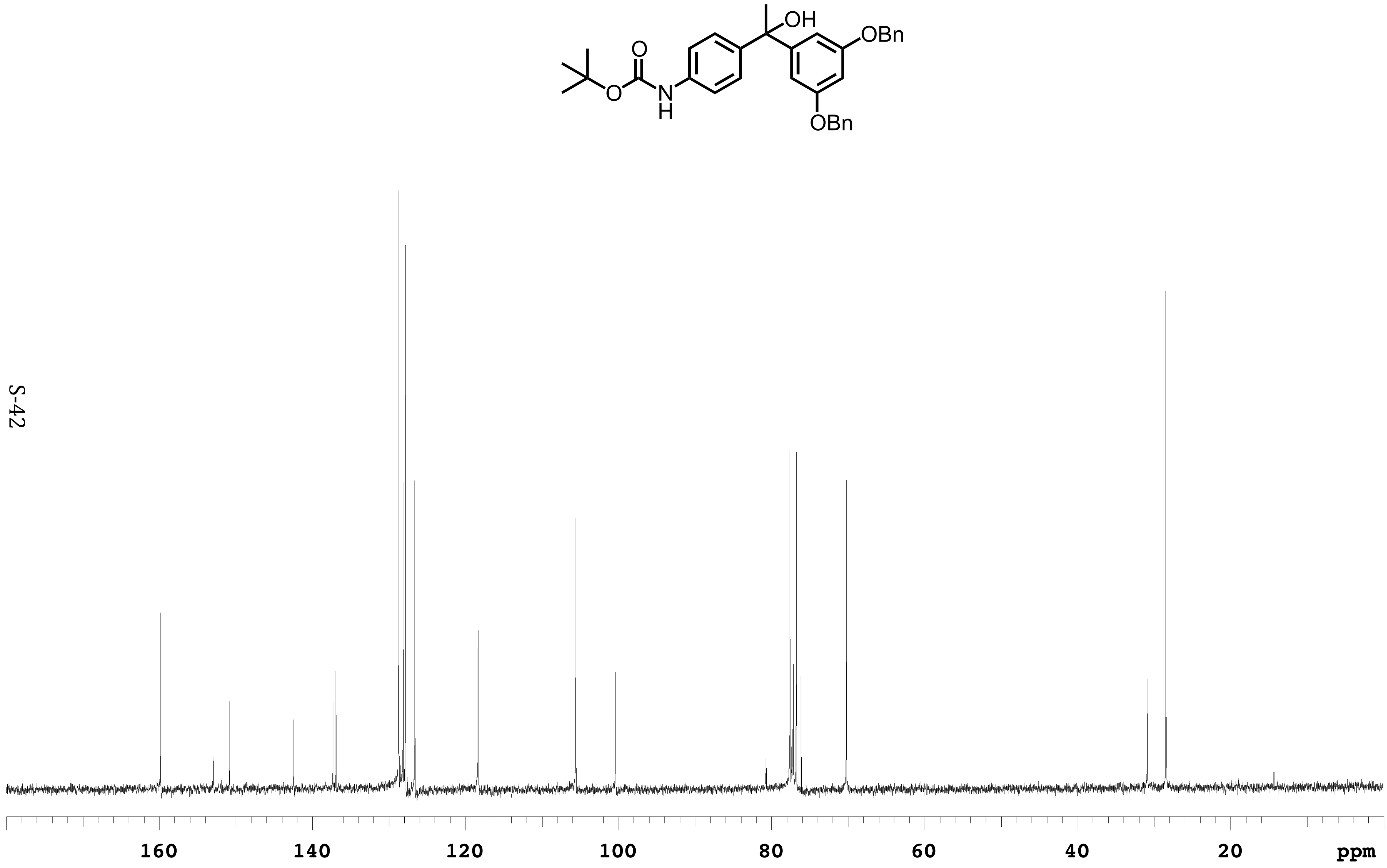
OH
OBn
O
O
N
H
OBn
160
140
120
100
80
60
40
20
ppm

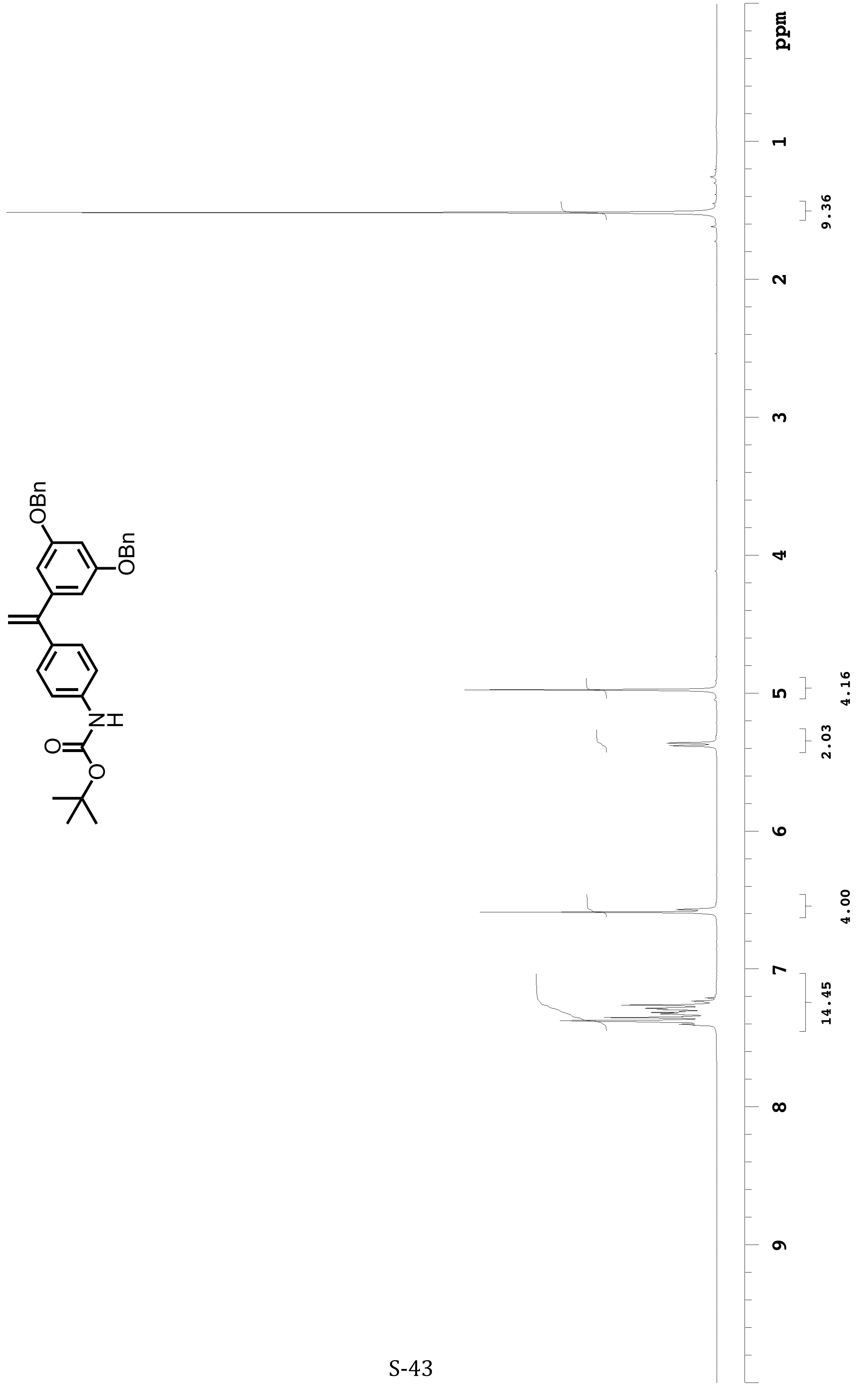
OBn
OBn
NH
O
O
ppm
1
2
3
4
5
6
7
8
9
9.36
4.16
2.03
4.00
14.45

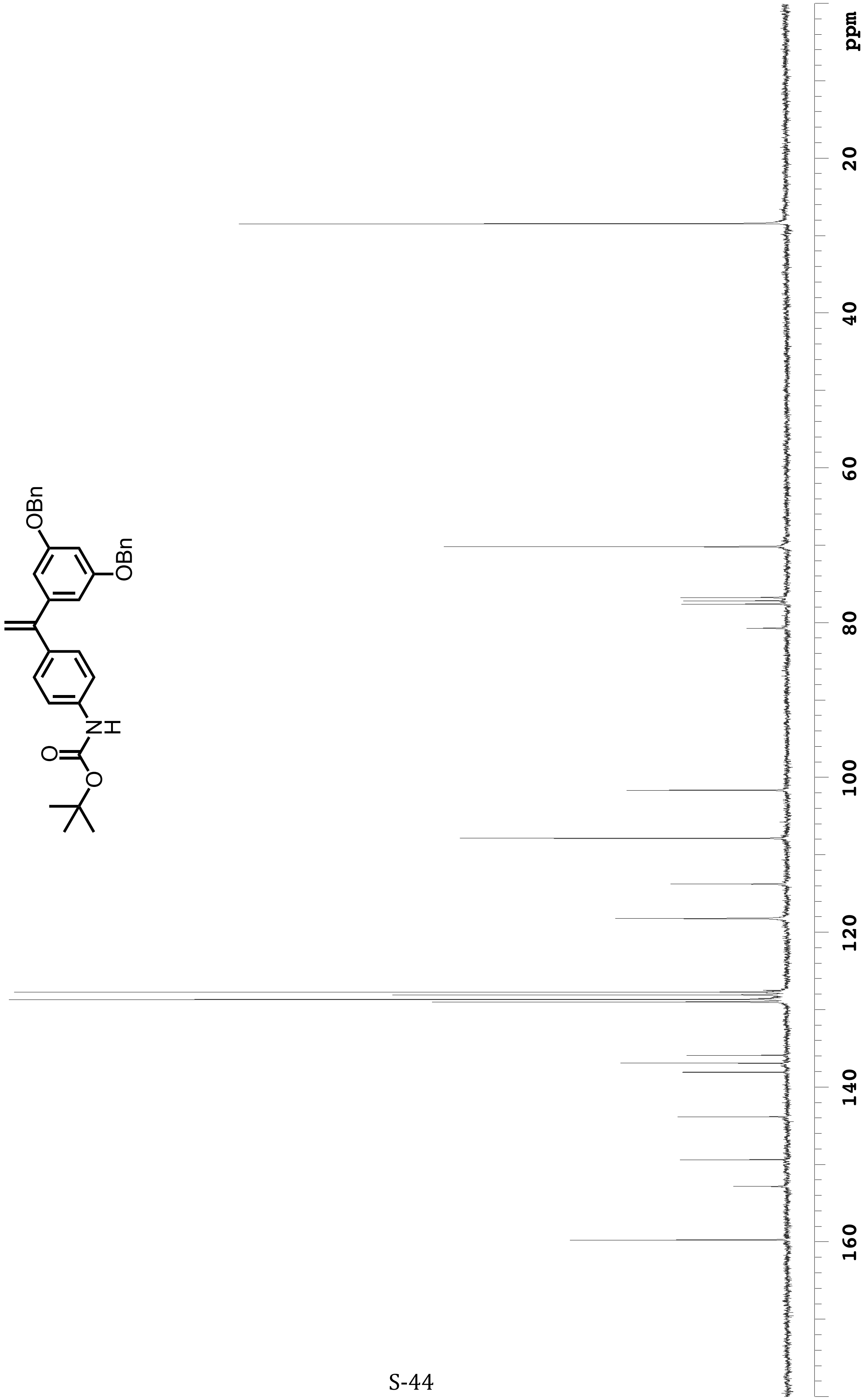
OBn
OBn
N
H
O
O
ppm
20
40
60
80
100
120
140
160

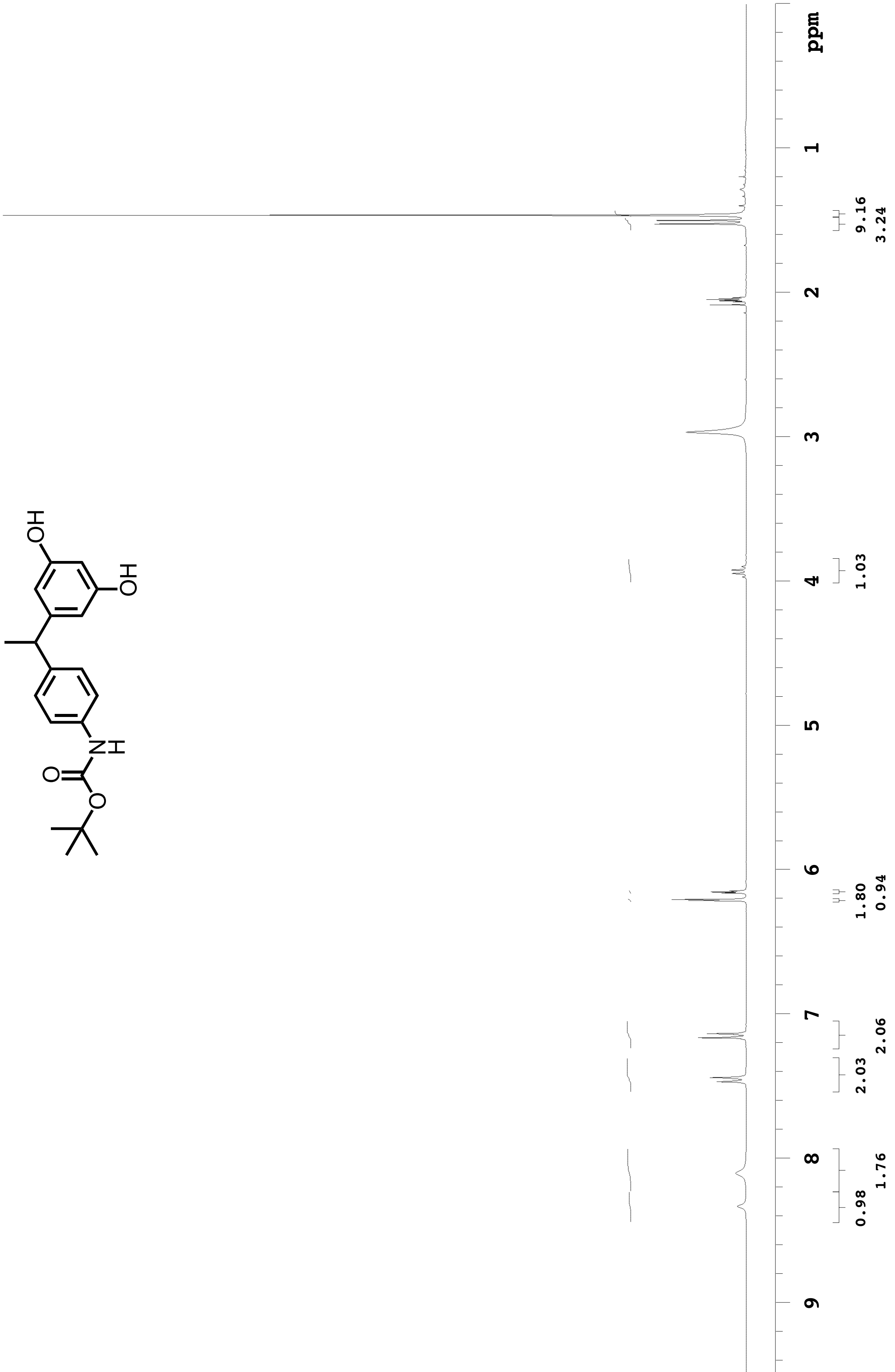
HO
OH
N
H
O
O
ppm
1
2
3
4
5
6
7
8
9
9.16
3.24
1.03
1.80
0.94
2.06
2.03
1.76
0.98

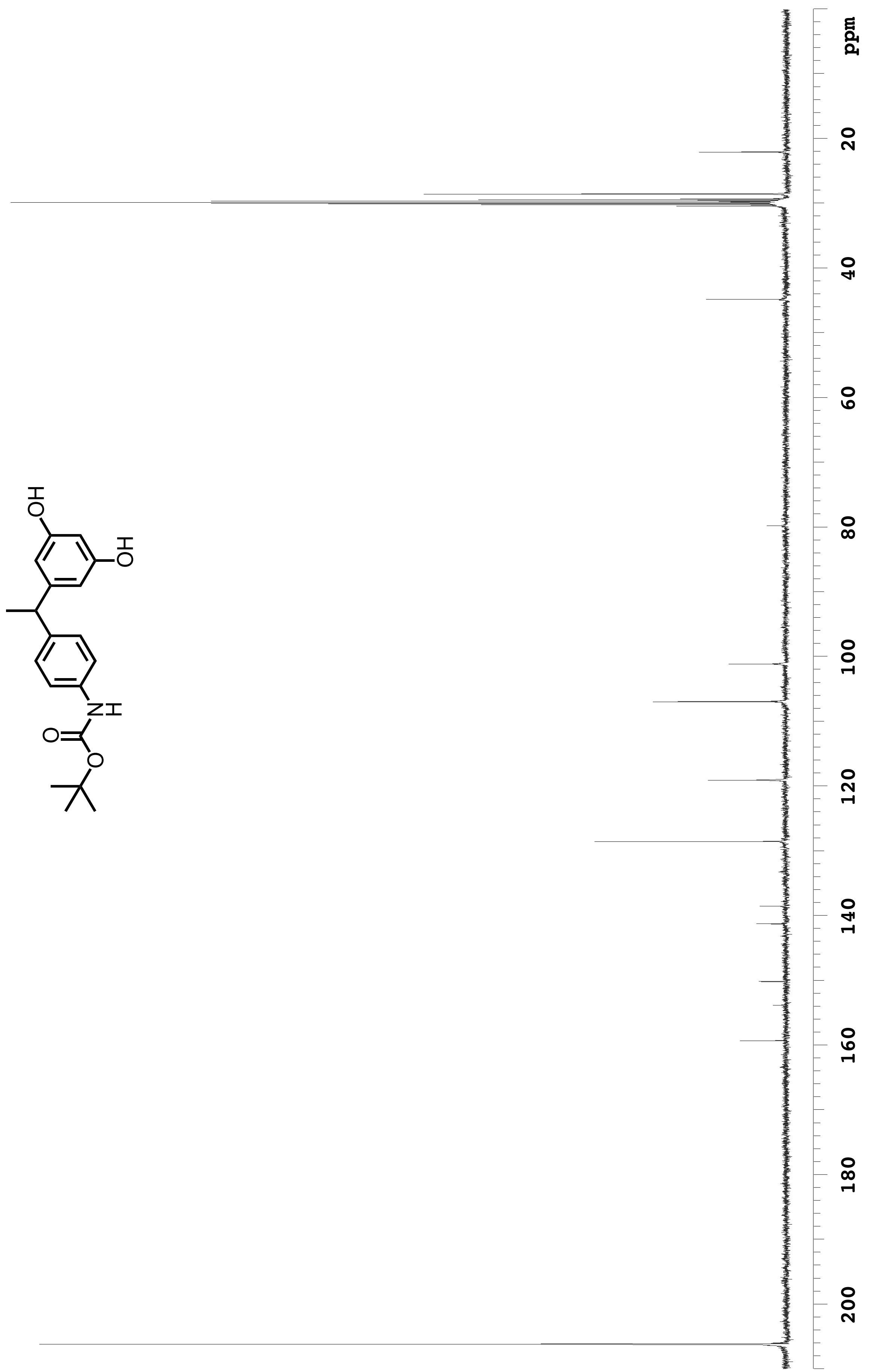
ppm
20
40
60
80
100
120
140
160
180
200
HO
OH
N
H
O
O

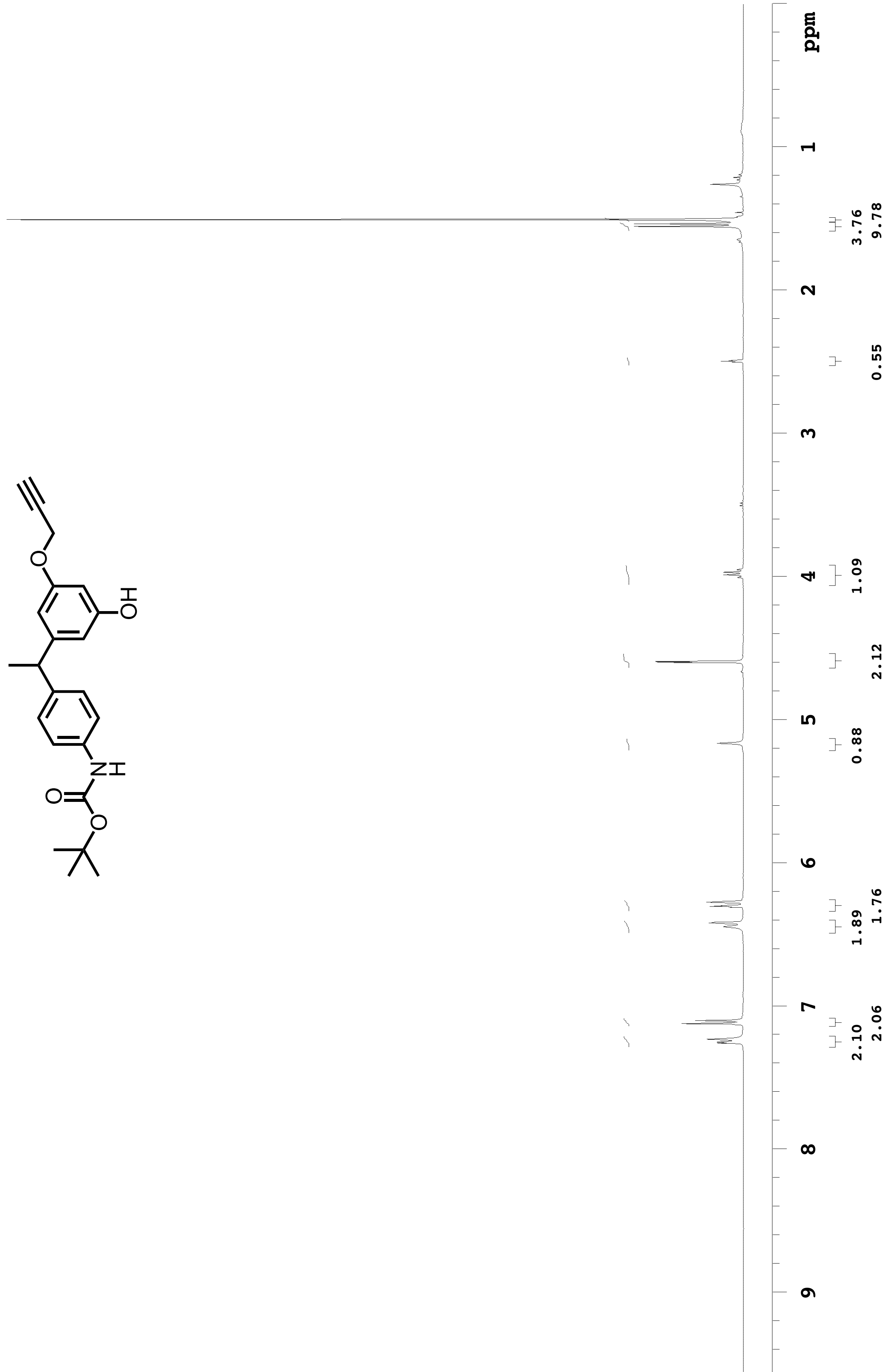
ppm
1
2
3
4
5
6
7
8
9
3.76
9.78
0.55
1.09
2.12
0.88
1.89
1.76
2.10
2.06
OH
O
NH
O
O

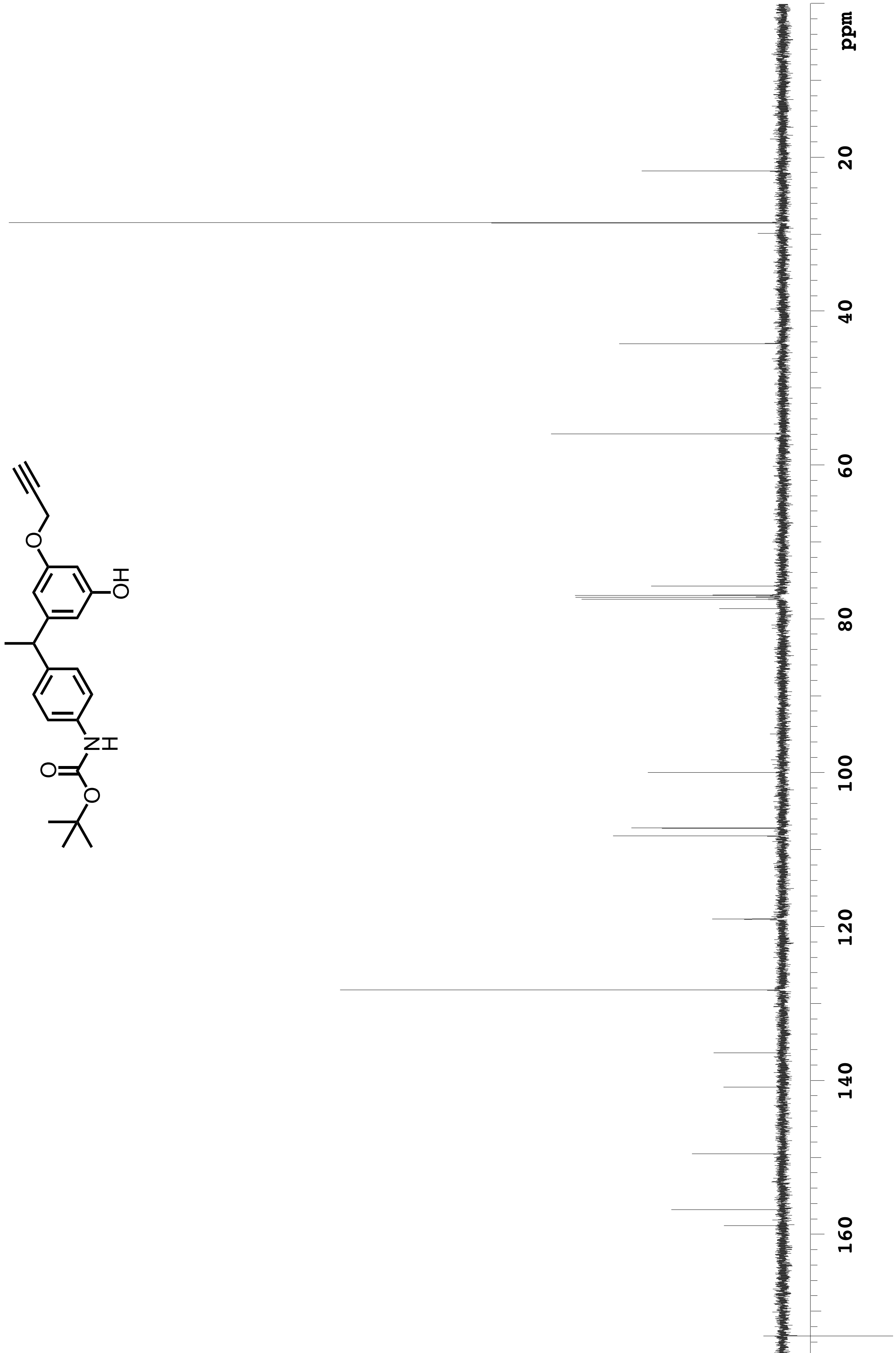
ppm
20
40
60
80
100
120
140
160

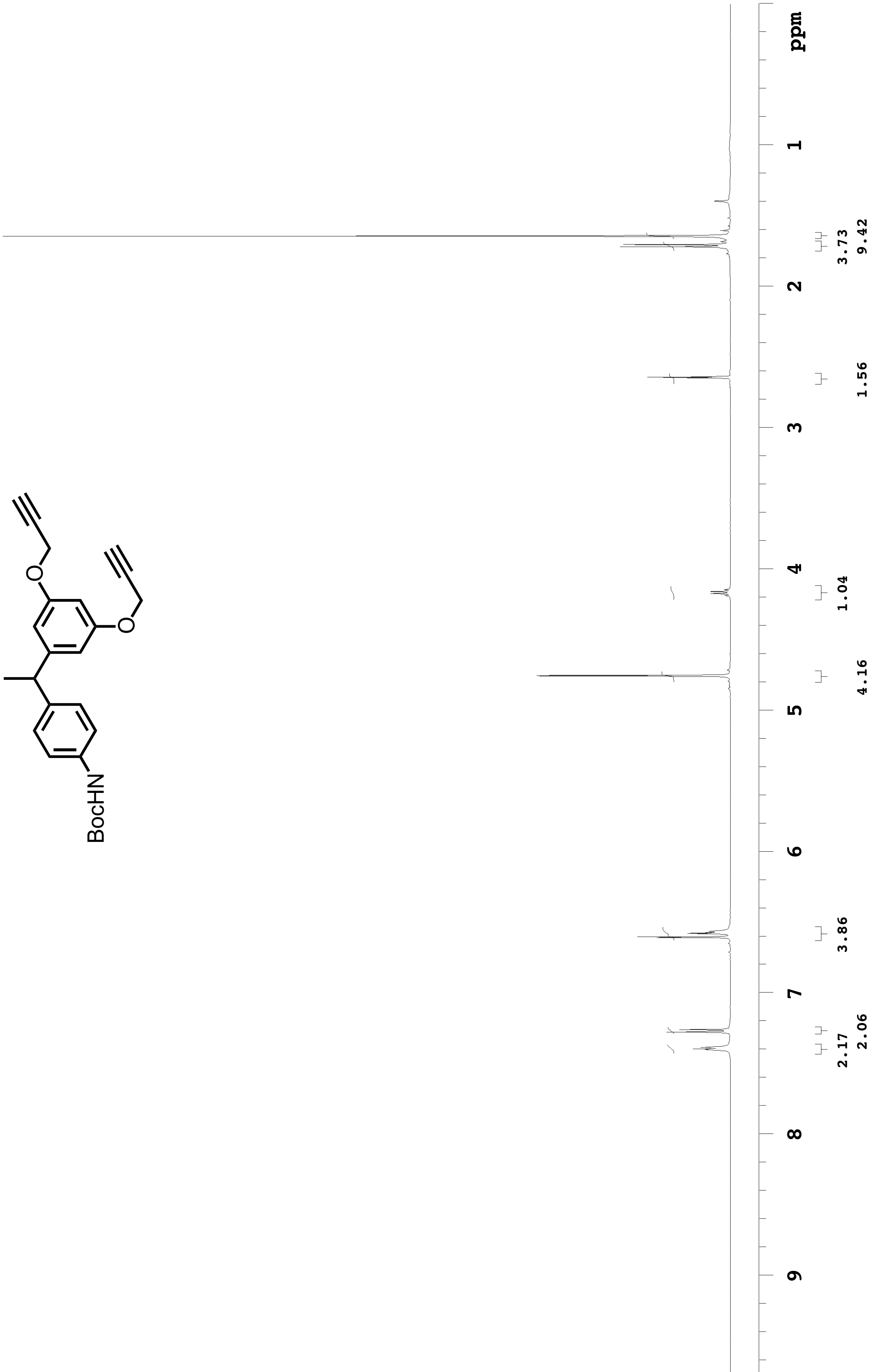
BocHN
O
O
ppm
1
2
3
4
5
6
7
8
9
3.73
9.42
1.56
1.04
4.16
3.86
2.17
2.06

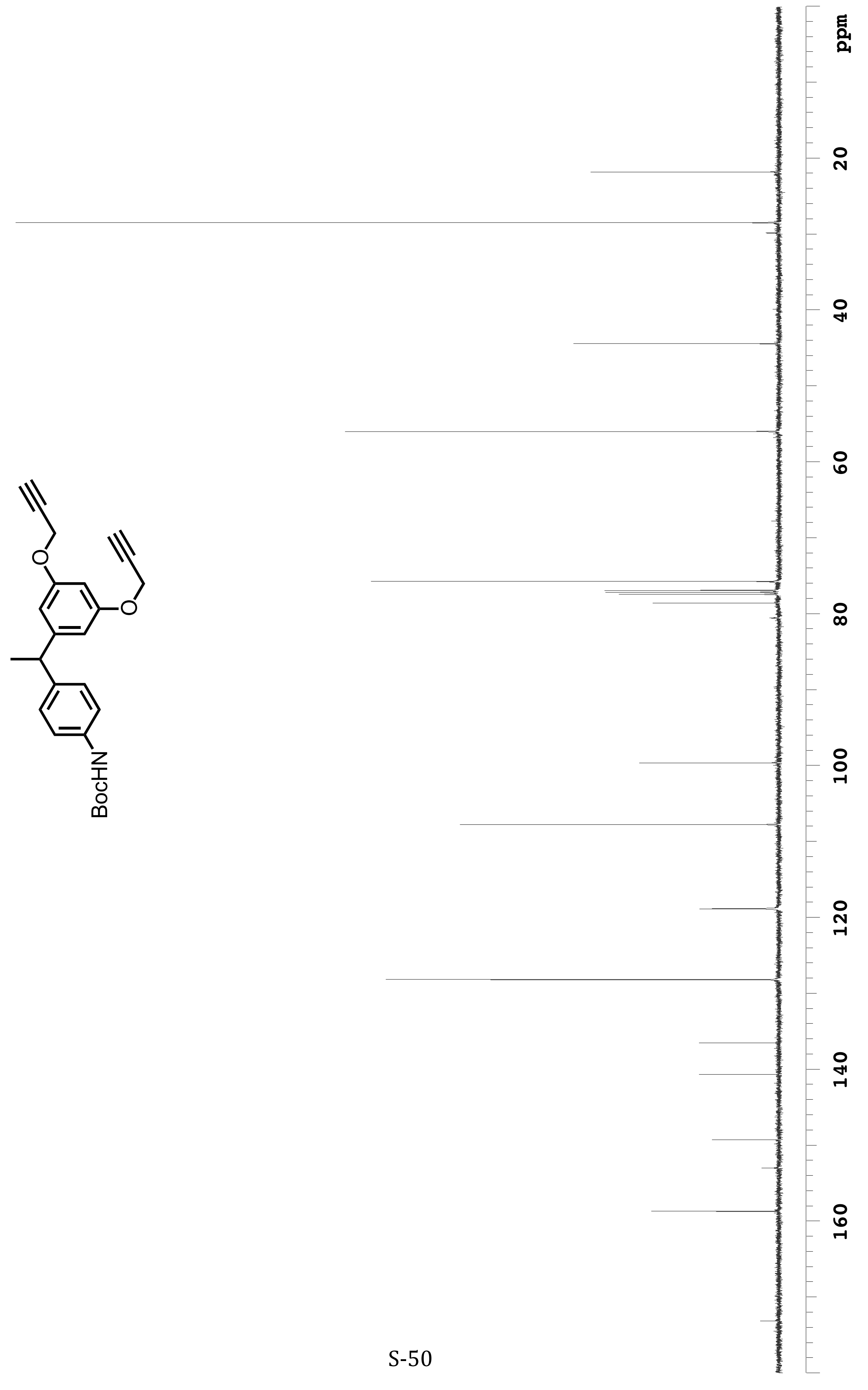
BocHN
O
O
ppm
20
40
60
80
100
120
140
160

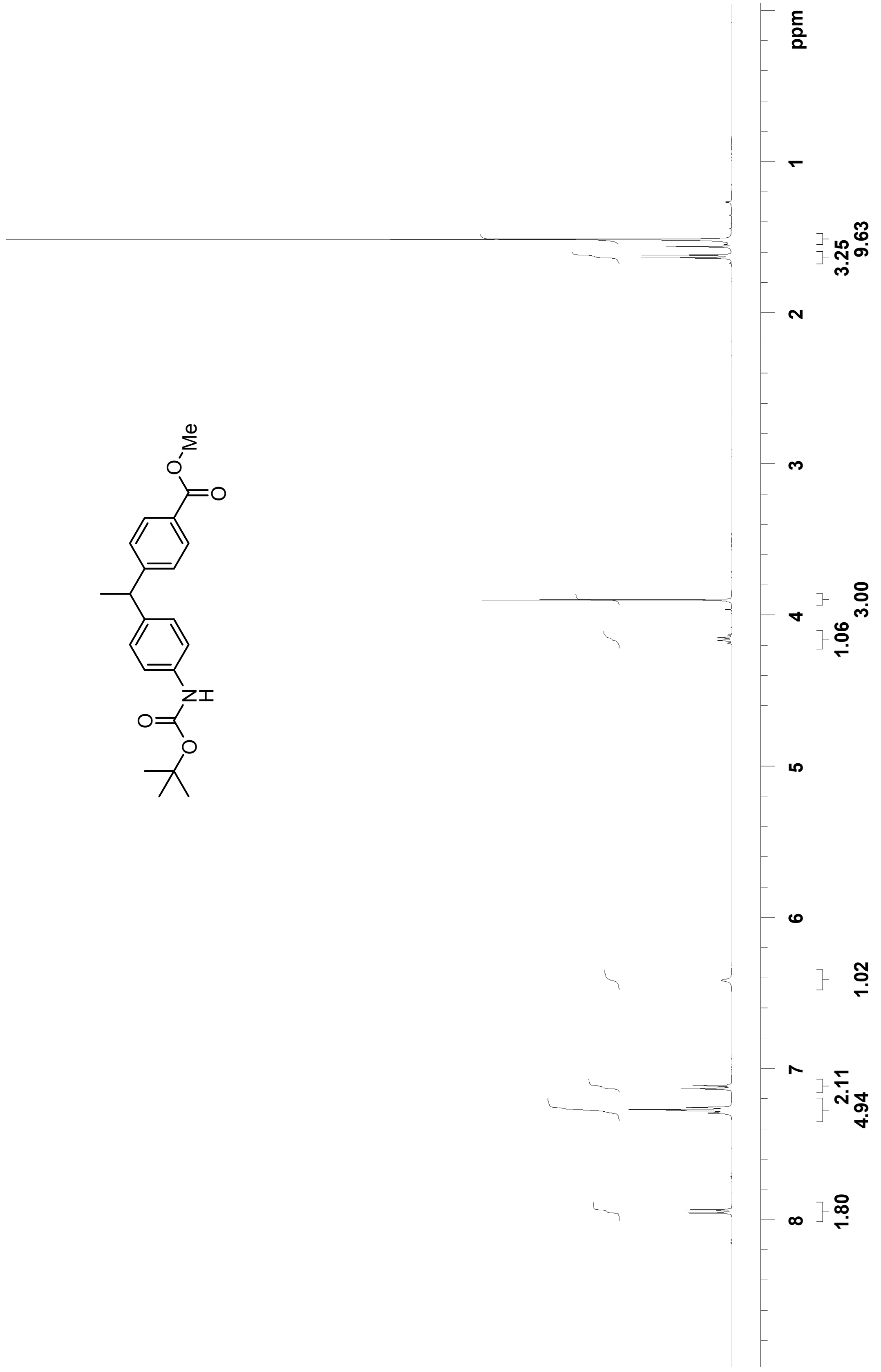

ppm
1
2
3
4
5
6
7
8
3.25
9.63
1.06
3.00
1.02
2.11
4.94
1.80
OMe
NH
O

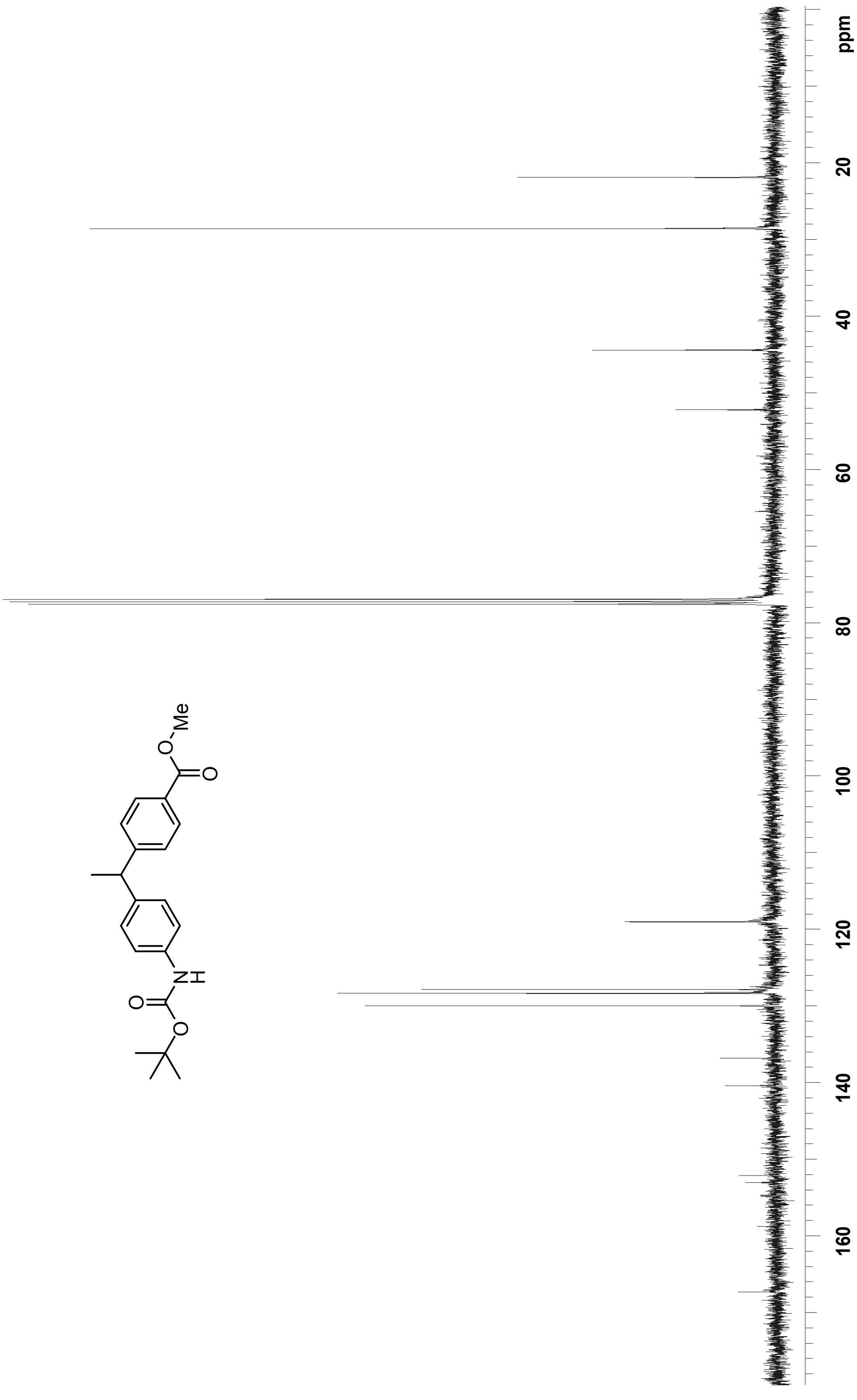
ppm
20
40
60
80
100
120
140
160
O
Me
O
N
H
O
O

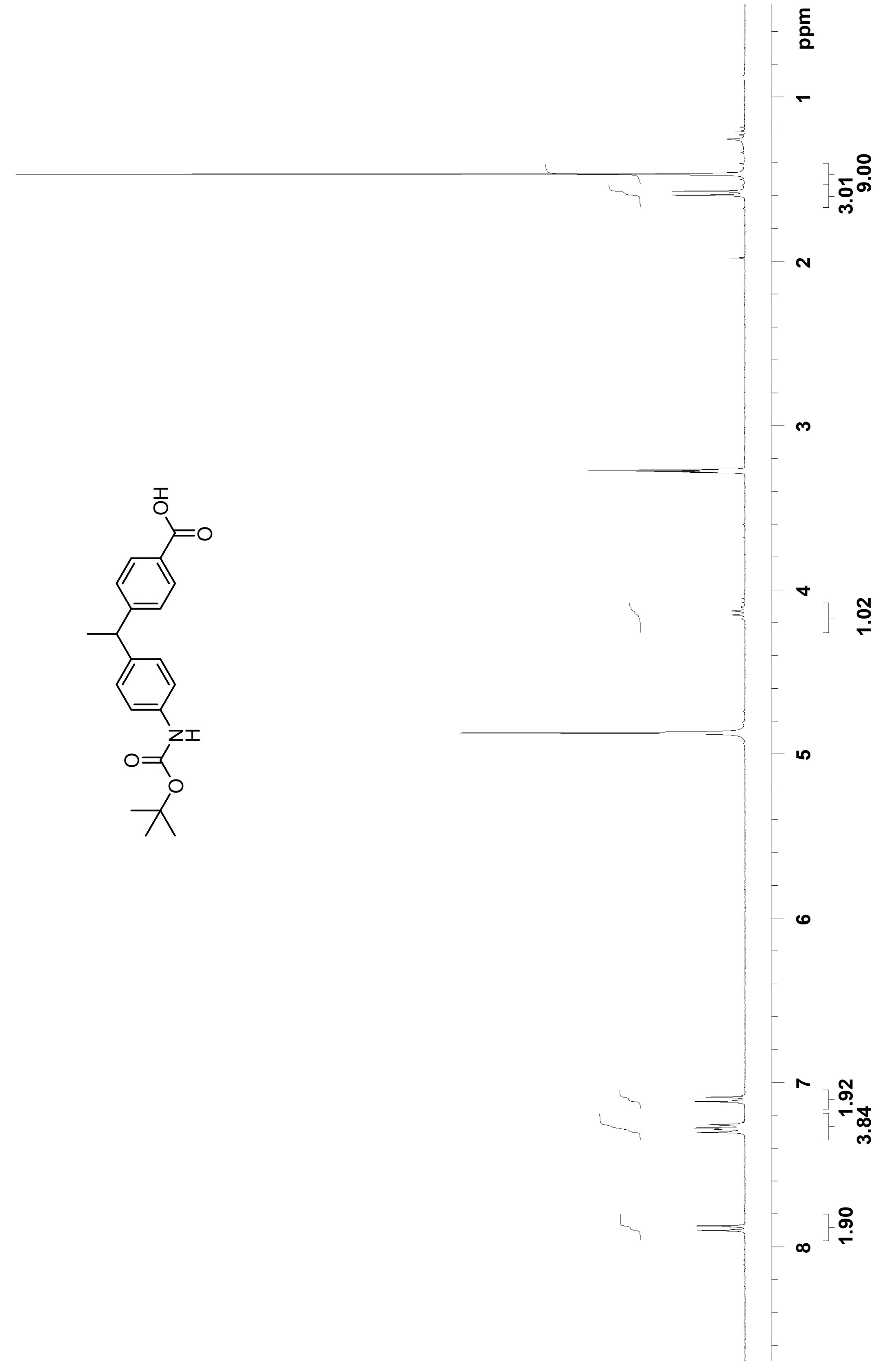

ppm
1
2
3
4
5
6
7
8
9.00
3.01
1.02
1.92
3.84
1.90
OH
O
NH
O
O

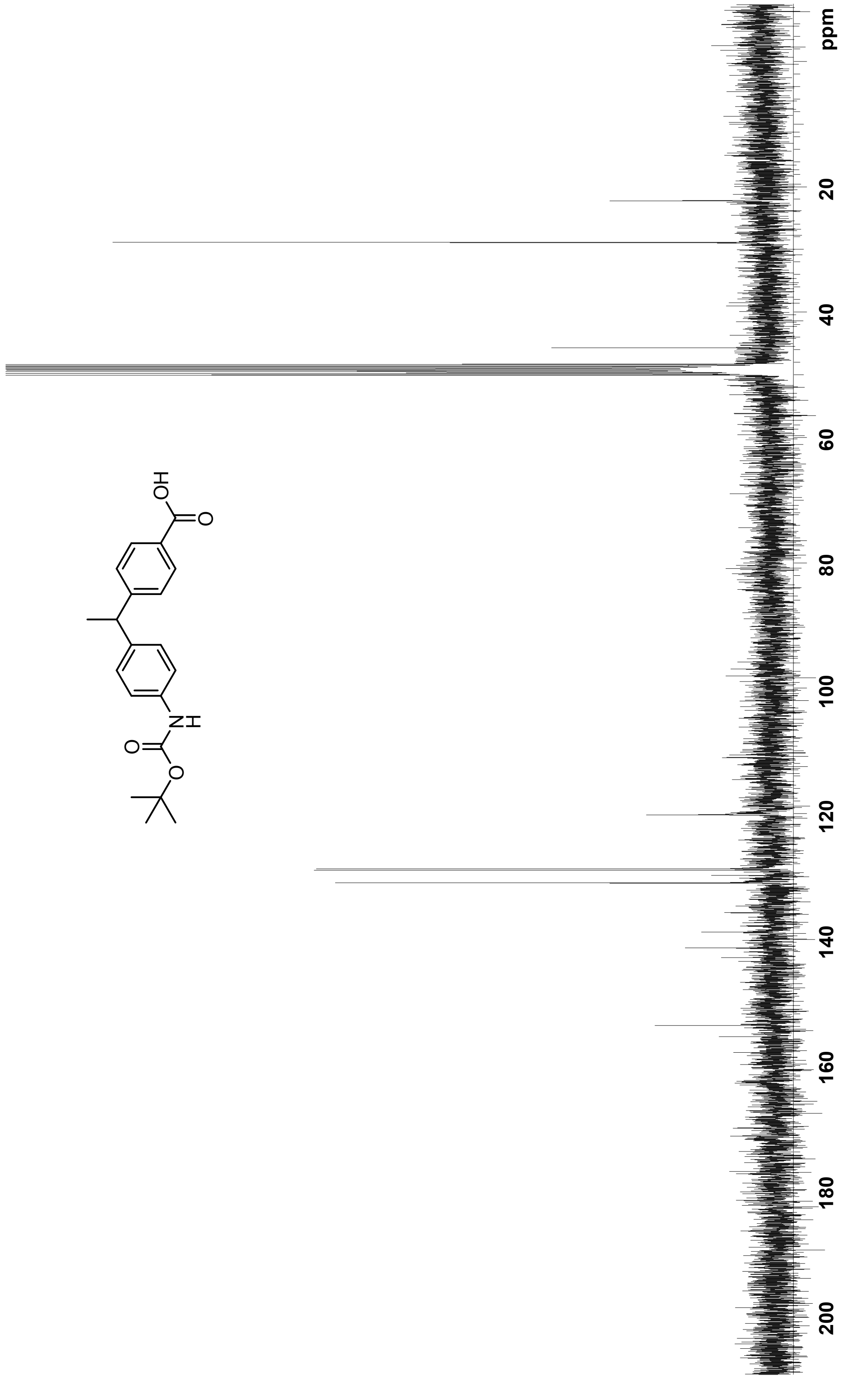
ppm
20
40
60
80
100
120
140
160
180
200

**References**


1. Mueller JA, Jensen DR, Sigman MS: **Dual Role of (−)-Sparteine in the Palladium-Catalyzed Aerobic Oxidative Kinetic Resolution of Secondary Alcohols**. *Journal of the American Chemical Society* 2002, **124**:8202-8203.
2. Mandal SK, Sigman MS: **Palladium-Catalyzed Aerobic Oxidative Kinetic Resolution of Alcohols with an Achiral Exogenous Base**. *The Journal of Organic Chemistry* 2003, **68**:7535-7537.
3. Navarro O, Kaur H, Mahjoor P, Nolan SP: **Cross-Coupling and Dehalogenation Reactions Catalyzed by (N-Heterocyclic carbene)Pd(allyl)Cl Complexes**. *The Journal of Organic Chemistry* 2004, **69**:3173-3180.
4. Li JJ, Li JJ, Li J, Trehan AK, Wong HS, Krishnananthan S, Kennedy LJ, Gao Q, Ng A, Robl JA, Balasubramanian B, Chen B-C: **A Synthesis of N-Bridged 5,6-Bicylic Pyridines via A Mild Cyclodehydration Using the Burgess Reagent and Discovery of A Novel Carbamylsulfonylation Reaction**. *Organic Letters* 2008, **10**:2897-2900.
5. Gligorich KM, Iwai Y, Cummings SA, Sigman MS: **A new approach to carbon–carbon bond formation: development of aerobic Pd-catalyzed reductive coupling reactions of organometallic reagents and styrenes**. *Tetrahedron* 2009, **65**:5074-5083.
6. Gligorich K, M.: **Development of Palladium-Catalyzed Alkene Functionalization Reactions with Applications to the Synthesis of Novel Anti-Cancer Compounds.** *Ph. D. Thesis.* Salt Lake City, UT: University of Utah; 2009.
7. Bhagwanth S, Waterson AG, Adjabeng GM, Hornberger KR: **Room-Temperature Pd-Catalyzed Amidation of Aryl Bromides Using tert-Butyl Carbamate**. *The Journal of Organic Chemistry* 2009, **74**:4634-4637.
8. Khlebnikov V, Patel K, Zhou X, Reddy MM, Su Z, Chiacchia FS, Hansen HC: **Synthesis of 2-aryl-4H-pyrano[2,3-b]pyridin-4-ones by a one-pot deprotection-cyclization reaction**. *Tetrahedron* 2009, **65**:6932-6940.
9. Azagarsamy MA, Sokkalingam P, Thayumanavan S: **Enzyme-Triggered Disassembly of Dendrimer-Based Amphiphilic Nanocontainers**. *Journal of the American Chemical Society* 2009, **131**:14184-14185.
10. Gligorich KM, Cummings SA, Sigman MS: **Palladium-Catalyzed Reductive Coupling of Styrenes and Organostannanes under Aerobic Conditions**. *Journal of the American Chemical Society* 2007, **129**:14193-14195.
11. Iwai Y, Gligorich KM, Sigman MS: **Aerobic Alcohol Oxidation Coupled to Palladium-Catalyzed Alkene Hydroarylation with Boronic Esters**. *Angewandte Chemie International Edition* 2008, **47**:3219-3222.
