# Supplementary figures and images for: The small molecule C-6 is selectively cytotoxic against breast cancer cells and its biological action is characterized by mitochondrial defects and endoplasmic reticulum stress
Source: Breast Cancer Res. 2014 Nov 26;16:472. doi: 10.1186/s13058-014-0472-0 (PMC4303206; doi:10.1186/s13058-014-0472-0)

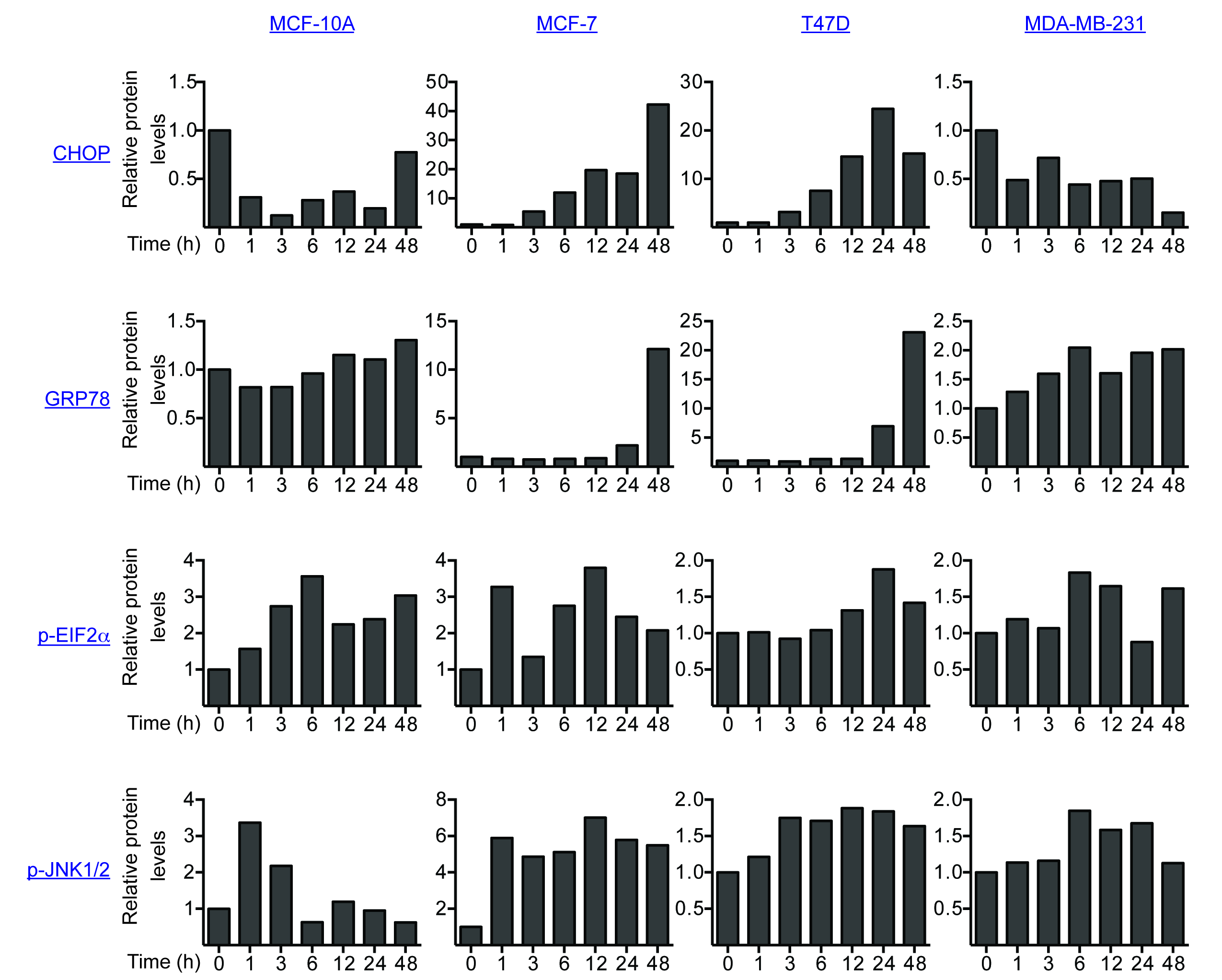

Supplement: Supplementary file 2 — Additional file 2: Densiometric analyses of western blots probing ER stress proteins. Densiometric plots were generated from the data presented in Figure 1D. The analysis was conducted by normalizing each protein band to its respective loading control. (TIFF 1 MB) [file 13058_2014_472_MOESM2_ESM.tiff]

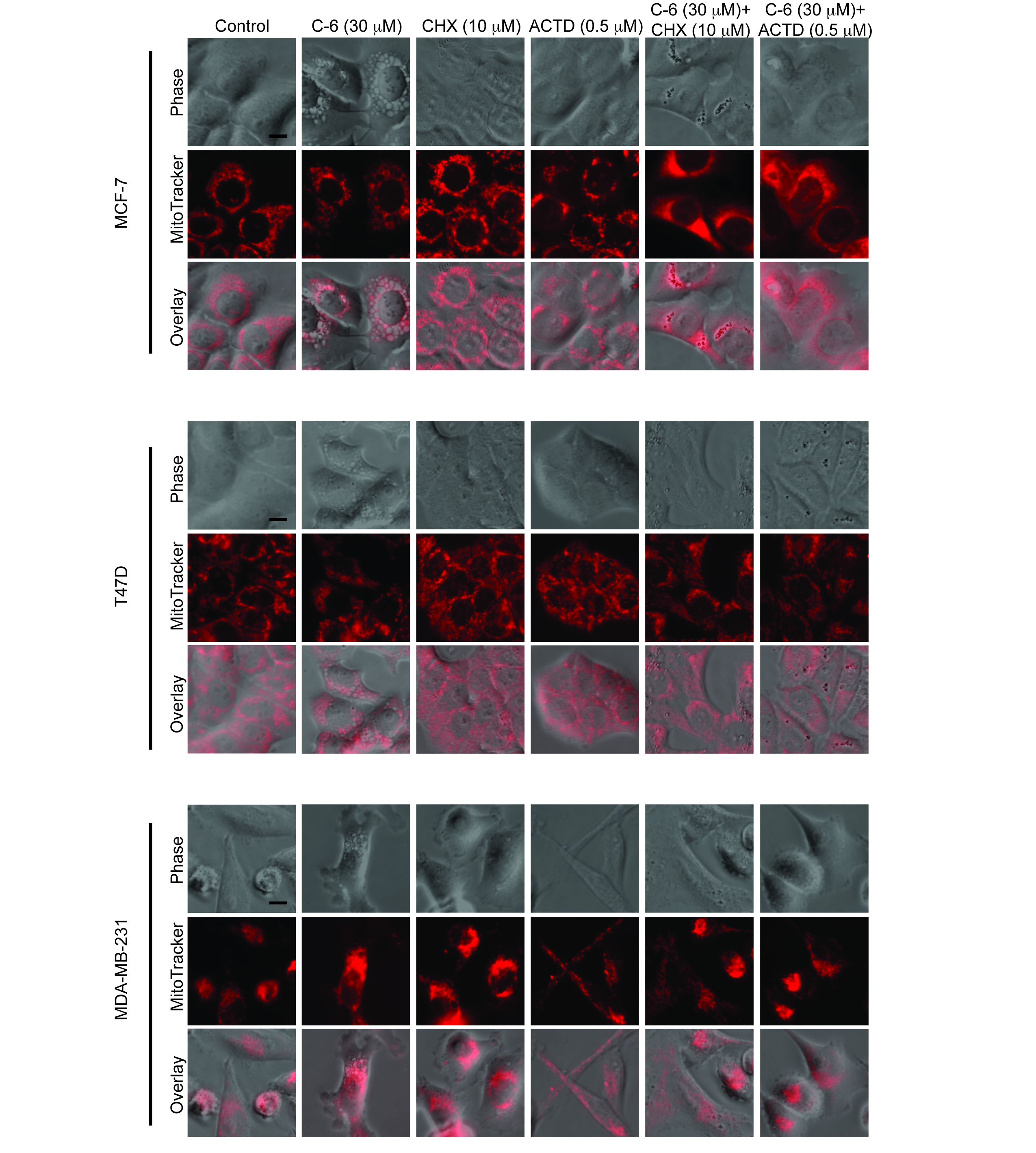

Supplement: Supplementary file 3 — Additional file 3: Inhibition of C-6-induced cytoplamic vacuolation by inhibitors of transcription and translation. MCF-7, T47D, and MDA-MB-231 cells were treated with C-6 (30 μM) for 24 hours with or without inhibitors of transcription (actinomycin D, ACTD) or translation (cycloheximide, CHX). Cells were then stained with MitoTracker and live imaging was conducted to assess mitochondrial morphology. (TIFF 11 MB) [file 13058_2014_472_MOESM3_ESM.tiff]

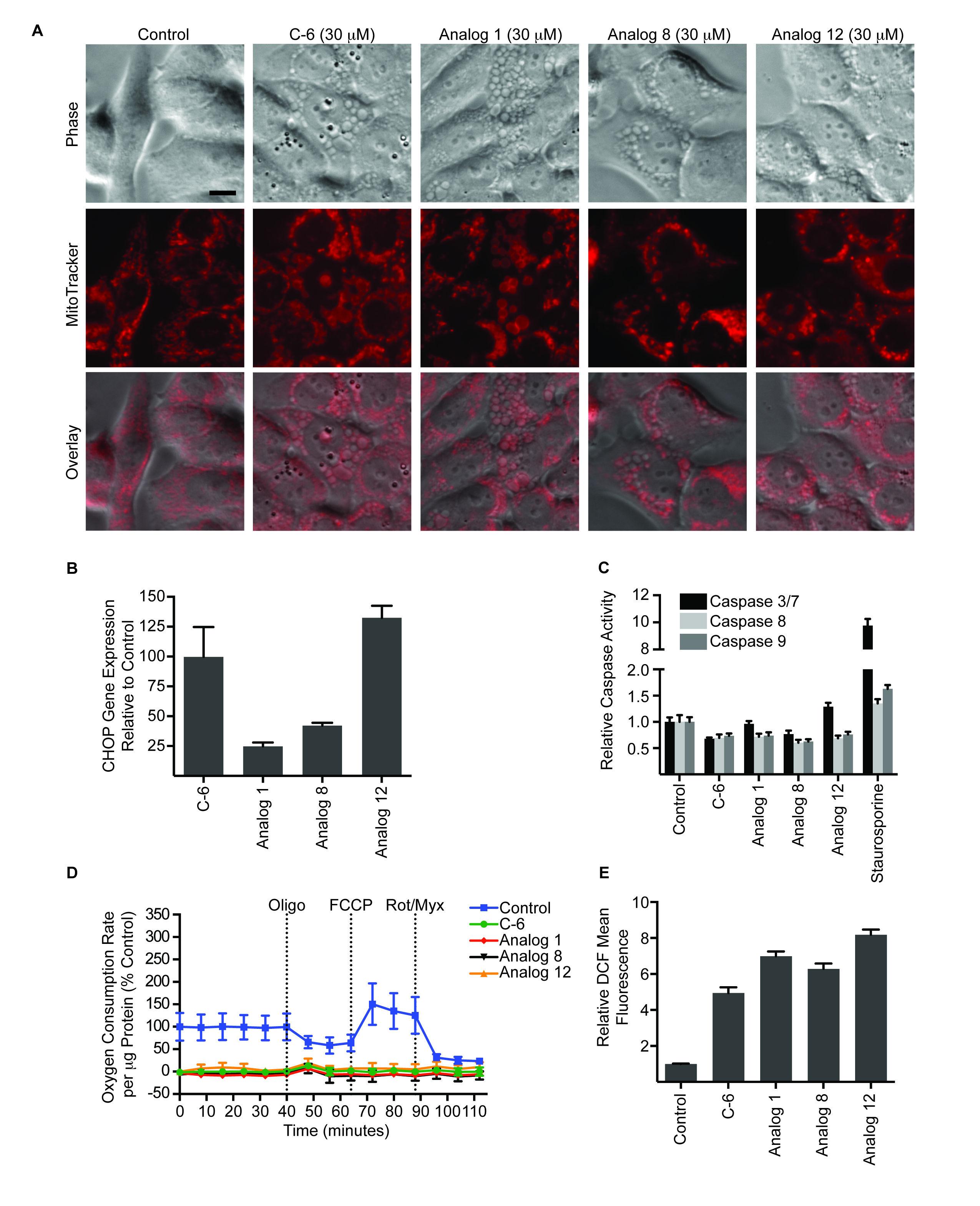

Supplement: Supplementary file 4 — Additional file 4: Mechanistic studies of C-6 analogs. (A) T47D cells were treated with C-6 (or analogs) for 24 hours, stained with MitoTracker, then imaged as live cells to assess mitochondrial morphology. (B) T47D cells were treated for 24 hours with either 30 μM C-6 or 30 μM of analog 1, 8, or 12 then CHOP gene expression was measured by real-time PCR. (C) Caspase 3/7, 8, and 9 activity was measured in T47D cells following 72 hours of small molecule treatment (each at 30 μM) or 18 hours of staurosporine (1 μM) treatment using the Promega Caspase-Glo assay system. (D) Measurement of oxygen consumption rates following pre-treatment of cells with either C-6 or analogs 1, 8, or 12 (each at 30 μM) for 24 hours. (E) Measurement of oxidative stress by DCF staining following 48 hours of treatment with 30 μM compound. (TIFF 9 MB) [file 13058_2014_472_MOESM4_ESM.tiff]

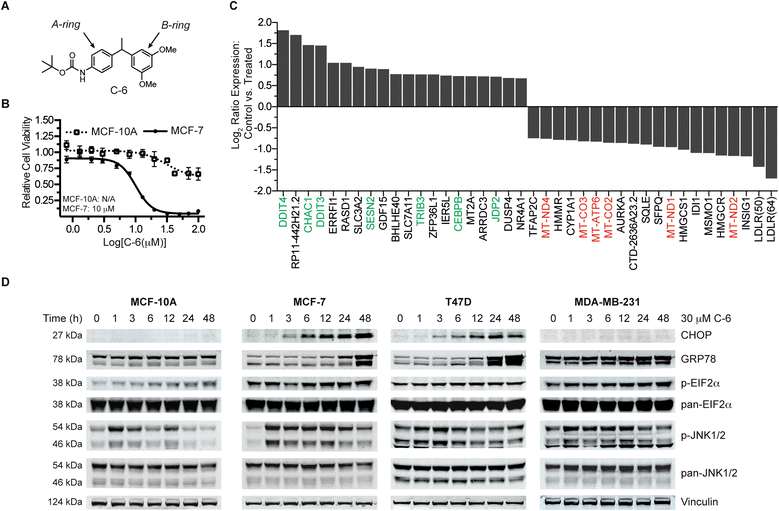

Supplement: Supplementary file 5 — Authors’ original file for figure 1 [file 13058_2014_472_MOESM5_ESM.gif]

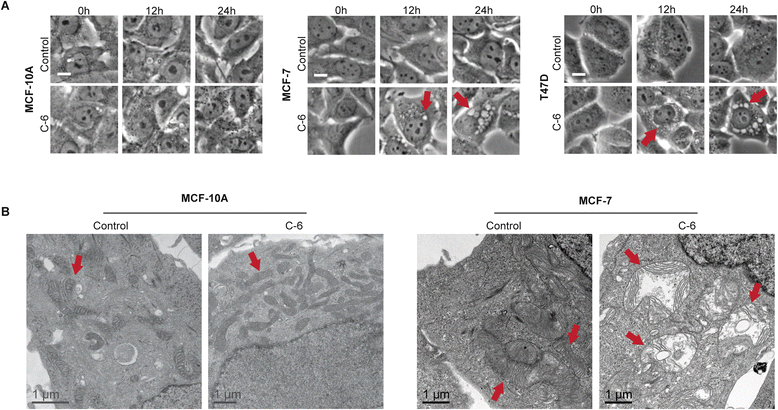

Supplement: Supplementary file 6 — Authors’ original file for figure 2 [file 13058_2014_472_MOESM6_ESM.gif]

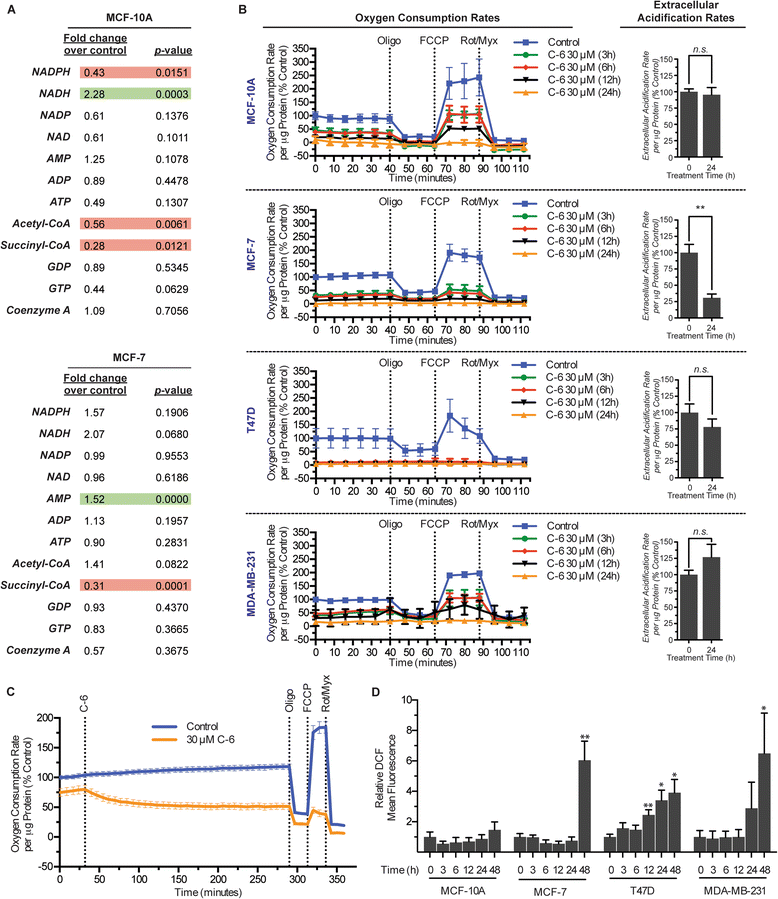

Supplement: Supplementary file 7 — Authors’ original file for figure 3 [file 13058_2014_472_MOESM7_ESM.gif]

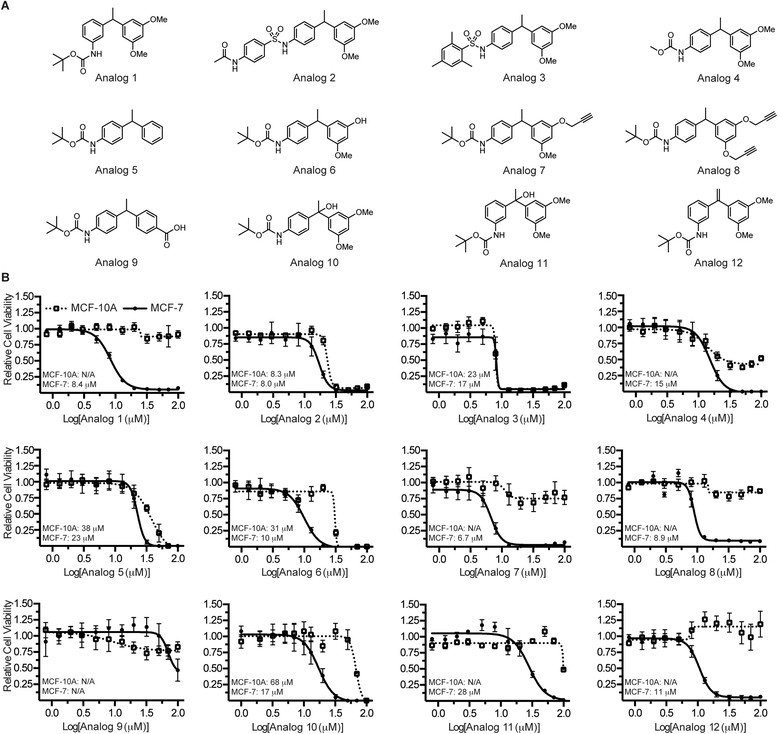

Supplement: Supplementary file 8 — Authors’ original file for figure 4 [file 13058_2014_472_MOESM8_ESM.gif]
